# Temporal Expression of Peripheral Blood Leukocyte Biomarkers in a *Macaca fascicularis* Infection Model of Tuberculosis; Comparison with Human Datasets and Analysis with Parametric/Non-parametric Tools for Improved Diagnostic Biomarker Identification

**DOI:** 10.1371/journal.pone.0154320

**Published:** 2016-05-26

**Authors:** Sajid Javed, Leanne Marsay, Alice Wareham, Kuiama S. Lewandowski, Ann Williams, Michael J. Dennis, Sally Sharpe, Richard Vipond, Nigel Silman, Graham Ball, Karen E. Kempsell

**Affiliations:** 1 Public Health England, Infection Services, Health Protection Agency Porton, Porton Down, Salisbury, Wiltshire, United Kingdom; 2 School of Science and Technology, Nottingham Trent University, Clifton Lane, Nottingham, United Kingdom; Public Health Research Institute at RBHS, UNITED STATES

## Abstract

A temporal study of gene expression in peripheral blood leukocytes (PBLs) from a *Mycobacterium tuberculosis* primary, pulmonary challenge model *Macaca fascicularis* has been conducted. PBL samples were taken prior to challenge and at one, two, four and six weeks post-challenge and labelled, purified RNAs hybridised to Operon Human Genome AROS V4.0 slides. Data analyses revealed a large number of differentially regulated gene entities, which exhibited temporal profiles of expression across the time course study. Further data refinements identified groups of key markers showing group-specific expression patterns, with a substantial reprogramming event evident at the four to six week interval. Selected statistically-significant gene entities from this study and other immune and apoptotic markers were validated using qPCR, which confirmed many of the results obtained using microarray hybridisation. These showed evidence of a step-change in gene expression from an ‘early’ FOS-associated response, to a ‘late’ predominantly type I interferon-driven response, with coincident reduction of expression of other markers. Loss of T-cell-associate marker expression was observed in responsive animals, with concordant elevation of markers which may be associated with a myeloid suppressor cell phenotype e.g. CD163. The animals in the study were of different lineages and these Chinese and Mauritian cynomolgous macaque lines showed clear evidence of differing susceptibilities to Tuberculosis challenge. We determined a number of key differences in response profiles between the groups, particularly in expression of T-cell and apoptotic makers, amongst others. These have provided interesting insights into innate susceptibility related to different host `phenotypes. Using a combination of parametric and non-parametric artificial neural network analyses we have identified key genes and regulatory pathways which may be important in early and adaptive responses to TB. Using comparisons between data outputs of each analytical pipeline and comparisons with previously published Human TB datasets, we have delineated a subset of gene entities which may be of use for biomarker diagnostic test development.

## Introduction

TB is a progressive, often fatal infectious disease, caused by the bacterium *Mycobacterium tuberculosis* and is a significant cause of morbidity and mortality worldwide. It is the seventh largest leading cause of death globally [1] and is second only to HIV as the largest cause of death due to an infectious disease. It is primarily a disease of poverty, particularly in developing countries [2]. Co-infection with HIV is common in low income countries and has a poor prognosis [3]. TB is a notifiable disease in the UK and is a prime concern for many governmental and other health bodies including the WHO, who have initiated control and treatment programmes like the Stop TB Partnership [4] and Stop TB Strategy [5]. Despite considerable investment in surveillance, control/treatment programmes and in research or development for new diagnostics and therapeutics, TB control and eradication has proved challenging to achieve in the UK and globally [1,6]. In high income countries this may be in part due to difficulties in diagnosis of affected individuals from areas of high endemic disease [7–10] at point of entry. Delays in diagnosis also contribute to poor patient management and outcomes and may contribute to disease transmission [11–13].

Methods used for TB diagnosis have not changed significantly in recent years in many routine diagnostic laboratories [14] and current tests are still somewhat inadequate. There is substantial evidence that TB diagnosis is subject to significant error, with up to 52% under-diagnosis reported in some studies using comparative indices between TB diagnosis methods as measured against autopsy observations [11]. Timely, accurate and sensitive diagnosis is imperative for prompt medical intervention and to limit ongoing transmission of TB infection. Ongoing surveillance is also a critical cornerstone for implementation of preventative measures for disease control. This is a key priority for many health and immigration authorities, particularly at ‘point of entry’ for developed countries where the majority of TB cases are imported [7–9,13,15,16]. Accurate and timely diagnosis presents challenges [1,17,18], particularly with early stage or latent infection [2,7,19,20], where symptoms may not be apparent and where detection of the pathogen by culture, specific PCR or other methods is not achieved. Thus, continued development of improved diagnostic methods is crucial to provide robust means for ongoing detection and management of TB.

There has been considerable interest in alternative methods for diagnosis of infectious diseases using systems biology approaches for host biomarker expression, including TB [21–24]. This approach has proved useful in a variety of diseases [25,26] including viral [27–29], bacterial [30] and other diseases [31,32]. A number of groups have published studies recently on host biomarker expression and discovery in human TB [32–45]. Most of these studies have focused on active and latent TB, compared to uninfected controls, but also in comparison to other diseases e.g. sarcoidosis and in TB- HIV co-infection. Many of these studies sought to identify TB-associated biomarkers of infection with a view to ongoing development of these entities as diagnostic targets. The Kaufmann group has trialled some of these markers in a clinical setting and shown good positive and negative predictive values for certain biomarker combinations [35,46,47]. To our knowledge similar studies have not been conducted for early, post-primary TB infection in humans, presumably due to inherent difficulties in identifying suitable patients for investigation.

For this purpose we have conducted a proof of concept, temporal differential gene expression study in peripheral blood leukocytes in aerosol-challenged non-human primate (NHP) pulmonary model of TB using Cynomolgus macaques (*Macaca fascicularis*). This was with a view to identification of host biomarkers associated with early exposure to *M*. *tuberculosis*. Microarray hybridisation analyses to human whole genome arrays revealed many substantial, temporal gene expression changes in peripheral blood leukocytes (PBL), in response to *M*. *tuberculosis* challenge. Using a similar model, studies have been conducted previously by members of this group to investigate disease processes and the role for interleukin-17, Th17 cells and iron homeostasis in protective immunity against TB [48–50]. Using systems biology approaches we have also identified a number of immunological pathways and interactions of importance in the response to TB infection in this model, which may demonstrate a bimodal post-primary immune response. The initial response appears to be associated with FOS expression, however as disease progresses this becomes predominantly type II interferon driven, with up-regulation of interferon-associated entities. However, there appears to be little expression of type I or type II interferons in these peripheral leukocytes. This may be due to a response driven by local expression at the site of infection, which is reflected in a distal response in circulating peripheral leukocytes, remote from an ongoing localised tissue/organ-based inflammatory response. Interestingly, we have also observed differences in the response profile in primates from different origin corresponding with innate TB susceptibility profiles, although there are features common to both.

Data analyses using both parametric and non-parametric (artificial neural network analysis (ANN)) bioinformatics analysis tools, have identified profiles of highly significant NHP biomarkers associated with ongoing inflammatory responses. Comparison with data from this and previously published human datasets has delineated a subset of markers of potential development as tools for diagnosis of active tuberculosis. A number of biomarker candidates have been validated using quantitative real-time PCR which show good potential during disease progression as diagnostic targets, which should exhibit improved utility across individuals from diverse ethnic origins. Ongoing progression and further development of these biomarker entities shared with human disease is being conducted with a view to development as diagnostic and prognostic markers of early and late TB infection.

## Material and Methods

### 2.1. Infection Model

#### 2.1.1. Primate Origin and Management

The samples analysed in this investigation were collected from six Cynomologus macaques (*Macaca fascicularis*) aged between 2–4 yrs in a pilot study set up to characterise and compare the outcome of high dose *M*. *tuberculosis* aerosol challenge, using previously described methods [50]. None of the animals had been used previously for experimental procedures. They were confirmed as naïve in terms of prior exposure to mycobacterial antigens (*M*. *tuberculosis* infection or environmental mycobacteria), by negative tuberculin test while in their original breeding colony and prior to the start of the study using the gamma interferon (IFN-γ)-based Primagam test kit (Biocor; CSL). The animals were of two distinct genotypes; Chinese (diverse) and Mauritian (restricted). Following aerosol exposure animals were monitored for 12 weeks for changes in behaviour and clinical parameters such as body weight, temperature, anaemia and inflammation. Disease progressed to meet endpoint criteria in all three Cynomolgus macaques of the Mauritian genotype six weeks after challenge. The macaques of Chinese genotype controlled disease for the remainder of the study period and were euthanized twelve weeks after the challenge.

The Cynomolgus macaques of Mauritian genotype were obtained from an established UK breeding colony, whilst the Cynomolgus macaques of Chinese genotype were obtained from a UK Home Office approved breeding facility in China. Absence of previous exposure to mycobacterial antigens was confirmed by a tuberculin skin test and screening using an ex-vivo IFNγ ELISPOT (MabTech, Nacka. Sweden) to measure responses to PPD (SSI, Copenhagen, Denmark), and pooled 15-mer peptides of ESAT6 and CFP10 (Peptide Protein Research LTD, Fareham, U.K.). The animals were housed in compatible social groups, in accordance with the Home Office (UK) Code of Practice for the Housing and Care of Animals Used in Scientific Procedures (1989), (now updated to Code of Practice for the housing and Care of Animals Bred, Supplied or Used for Scientific Purposes, December 2014, and the National Committee for Refinement, Reduction and Replacement (NC3Rs), Guidelines on Primate Accommodation, Care and Use, August 2006 (NC3Rs, 2006)). Animals were sedated by intramuscular (IM) injection with ketamine hydrochloride (Ketaset, 100mg/ml, Fort Dodge Animal Health Ltd, Southampton, UK; 10mg/kg) for procedures requiring removal from their housing. All animal procedures were approved by the Public Health England Ethical Review Committee, Porton Down, UK, and authorized under an appropriate UK Home Office project license.

The challenge was performed on sedated animals placed within a “head-out” plethysmography chamber (Buxco, Wilmington, NC), to enable the aerosol to be delivered simultaneously with the measurement of the respiration rate. This process has been shown previously to deliver a known number of viable *M*. *tuberculosis* bacilli in a target volume of inspired aerosol. Clinical assessment post-challenge including animal behaviour was observed daily throughout the study for contra-indicators such as depression, withdrawal from the group, aggression, food and water intake, changes in respiration rate, or cough. Animals were sedated every week to measure weight, body temperature, blood haemoglobin levels, and erythrocyte sedimentation rate (ESR) and to collect blood samples for immunology and RNA extraction.

### 2.2. Purification of Total RNA from Non-Human Primate Peripheral Blood

Whole heparinised blood was obtained at three independent time-points prior to challenge and at one, two, four and six weeks post *M*. *tuberculosis* challenge. Within one hour of collection, 1 ml of blood from each animal was mixed with 5 ml of Erythrocyte Lysis (EL) Buffer (Qiagen) followed by incubation on ice for 10–15 minutes. Peripheral blood leukocytes (PBLs) were recovered from erythrocyte-lysed blood by centrifugation at 400 x g for 10 minutes at 4°C and re-suspended in a further 2 ml of EL buffer. PBLs were again recovered by centrifugation as described above and processed for recovery of total RNA.

One ml of TRIzol was added to the PBL pellet, then total RNA was extracted from the lysed PBL pellet according to the manufacturer’s instructions (www.Invitrogen.com) using aqueous-phase separation with chloroform isoamyl alcohol and the precipitation using 2-isopropanol. Recovered, dried RNA pellets were re-suspended in 10 μl of diethylpyrocarbonate (DECP) water (Invitrogen), then concentration and purity (A_260_/A_280_ ratio ≥ 1.8) assessed by spectrophotometry using a NanoDrop ND-1000 spectrophotometer (Thermo Scientific). Genomic DNA was removed prior to its use in further procedures using the DNase I kit (Qiagen), according to the manufacturer’s instructions.

### 2.3. Amplification of Total Non-Human Primate Peripheral Blood RNA

Due to the small volumes of blood used in the study and consequently low yield of total RNA recovered, an enrichment step was then performed using the Genisphere SenseAmp RNA amplification kit according to manufacturer’s instructions (http://genisphere.com/). The resulting amplified mRNA was purified using RNeasy Min-Elute Cleanup kit (Qiagen), again according to the manufacturer’s protocol. The mRNA concentration and purity (A_260_/A_280_ ratio ≥ 1.8) was then assessed by spectrophotometry using a NanoDrop ND-1000 spectrophotometer.

### 2.4. Fluorescence Labelling of Non-Human Primate Amplified RNA and Hybridisation to Operon Whole Human Genome Microarrays

Total amplified primate PBL mRNAs from each time-point were labelled with Cy3-labelled dCTP as described previously [51,52] and hybridised to replicate Operon Human Genome AROS V4.0 slides (n = 3/ sample/time-point (http://www.microarrays.com/dna-arrays.php). This is a human oligonucleotide microarray comprising some 35,035 oligonucleotide probes, which represent approximately 25,100 unique genes and 39,600 transcripts. A subset of the total probe set (31,387 probes) is contained within the span of a single exon to provide the microarray detection precision at both the transcript and gene levels. Microarray slides were pre-hybridized for 30 minutes at 42°C in a hybridization solution containing 5 x standard saline citrate (SSC), 0.1% sodium dodecyl sulfate (SDS) and 4 x Denhardts solution, followed by a 1 minute wash in molecular reagent grade double distilled water then a brief rinse in isopropanol. The slides were then dried by centrifugation at 1500 rpm for 5 minutes. Prior to hybridization, 20 μg of Cy3-labelled mRNA was combined with 20 μg of Cot1 Human DNA (10 μg/μl) and 20 μg of polyA RNA (10 μg/μl) (Invitrogen) to a final volume of 40 μl in RNAase-free water and denatured at 95°C for 2 minutes to denature the mRNA target. The mixture was then mixed 1:1 with 2 x concentrate hybridization solution (10 x SSC, 0.2% SDS, 8 x Denhardts solution) pre-warmed to 65°C. The microarray slide was placed in the chamber of a Slidebooster microarray hybridisation platform (Olympus Advalytix, Germany) pre-heated to 42°C and a lifterslip covering the area of the array affixed in place (http://www.thermoscientific.com/content/tfs/en/ product/lifterslips-cover-slips-microarray-slides.html). The prepared sample was applied to the array and drawn under the lifterslip by capillary action. This was then hybridised at 42°C for 6 hours in the presence of proprietary formamide-free AdvaHum humidifying buffer (Olympus Advalytix, Germany) at maximum mixing power (M27). After completion of hybridisation, lifterslips were removed and the slides were washed in two separate wash solutions for two minutes each at 42°C—Buffer A (1x SSC 1% SDS) Buffer B1 (0.1x SSC 1% SDS), then a further wash in Buffer B2 (1% SSC) for two minutes at room temperature. The slides were air-dried and scanned using an Affymetrix 480 microarray scanner, at a gain of 65.

### 2.5. Data Analysis

#### 2.5.1. Feature Extraction and Quantification

Feature extraction was conducted using the microarray quantification package BlueFuse (BlueGnome; now a subsidiary of www.illumina.com). Raw data were exported and hybridisation fluorescence intensities quantified using default background subtraction and normalisation methods, to remove data generated from poor-quality spots and hybridisation artefacts. All raw data were then processed further using the microarray analysis package Genespring 12.5. All normalised and raw data are deposited in GEO under accession number GSE76703.

#### 2.5.2. Data normalisation and Parametric Statistical Analysis

Data output files from BlueFuse were imported into GeneSpring 12.5 (GX12.5) for differential gene expression and statistical analysis. Raw data were normalized to the 50^th^ percentile followed by median baseline transformed to each animal’s corresponding pre-bleed sample. This was conducted to normalise data across all time-points and assess differential gene expression of each gene entity, relative to a baseline i.e. pre-bleed level of expression prior to *M*. *tuberculosis* challenge. Mean values across three replicate sample slides were used for further ongoing analysis. Data were assessed for quality, then filtered on gene expression where entities in all samples and all conditions had normalised expression values within the cut-off -10.699 to 7.037. Statistically significant features were identified using one-way ANOVA analysis across all entities and time-points, using either the Benjamini-Hochberg False Discovery Rate (BH-FDR), or the more parsimonious Bonferroni Family-Wise Error Rate (B-FWER), with multiple testing corrections at a cut-off p ≤ 0.05. To identify temporally, differentially expressed entities between time-points post-infection, fold-change cut-off analyses were conducted using the default cut-off setting ≥ 2.0 all referenced against the pre-bleed condition, where the minimum number of pairs was equal to one out of the four condition pairs i.e. weeks one, two, four or six. These were further analysed and depicted graphically using the heat map, hierarchical cluster analysis and other functions in Genespring 12.5, using default settings.

#### 2.5.3. Microarray Data Analysis using Artificial Neural Networks

Normalised expression data were analysed using an Artificial Neural Network (ANN) based data mining approach [53]. This approach comprised a supervised learning approach where the data for each probe on the array were used singly to classify a sample defined into one of two treatment groups. The classifier consisted of a multi-layer perceptron ANN, where weights were updated by a back propagation algorithm [54]. The ANN architecture utilised a constrained architecture of 2 hidden nodes to reduce the risk of over-fitting. ANN training incorporated Monte Carlo Cross Validation (MCCV), wherein the data were randomly divided into three subsets; 60% for training the classifier, 20% for testing (to assess model performance on unseen data and initiate early stopping to reduce overfitting) and 20% for validation (to independently test the model on data completely blind to the model). This MCCV process was repeated 50 times to generate predictions and associated error values for each sample with respect to the validation (blind) data. Probes were ranked in ascending order based on predictive root mean squared (RMS) error for the test data set from MCCV.

#### 2.5.4. Network Inference and Pathway Analysis

The top 100 ranked genes based on RMS error were selected for further analysis using an ANN based Network Inference approach [55]. This algorithm determines a weight for all of the potential interactions in the defined set (9900 in 100 probes), so that the magnitude of a probe’s influence in the contextualised probe set (top 100) can be determined. In this process, 99 genes are used to predict a single target (output) probe with a back propagation MLP ANN as described above. This model is then parameterized based on the weights of the trained optimised ANN model and the strength of each probe’s influence on the target determined. The target (output) probe is then changed to the next probe in the set, the remaining 99 probes becoming inputs to this second model. This model is then parameterized as before. The target (output) probe changes and parameterization steps are then repeated until all of the 100 probes in the set have been used as outputs. The parameterisation generates a matrix of all interactions between the top probes in both directions (9900 interactions (100x100)-100). This interaction matrix is then ranked based on the magnitude of interaction to eliminate all but the strongest interactions (outlined in [56]). These strongest interactions (100) were visualized with Cytoscape, creating a map showing the nature of the interactions between genes, the most connected probes were defined as hubs.

#### 2.5.5. Analysis of Previously Published Human Microarray Datasets and Comparison with NHP Data

Previously published human TB datasets were imported from the National Centre for Biotechnology Information Geo database (http://www.ncbi.nlm.nih.gov/gds/). Data from two independent human TB studies GSE19439 and GSE28623 were imported into GeneSpring 12.5 for analysis and comparison with NHP data from this study. Raw data were imported and normalized to the 75^th^ percentile followed by baseline transformation to the median of all samples. Data were assessed for quality, then filtered on gene expression where entities in all samples and all conditions had normalised expression values within the default cut-off for that dataset. Statistically significant features were identified using one-way ANOVA analysis using the Benjamini-Hochberg False Discovery rate (BH-FDR) multiple testing correction at a cut-off of p ≤ 0.05. The more parsimonious B-FWER multiple testing correction was not used on these data sets due to the low numbers of features remaining after analysis using this method on the two human datasets.

### 2.6. Quantitative real-time PCR (qPCR) assays

#### 2.6.1 qPCR Design

Quantitative PCR assays for Macaque genes of interest were designed as follows. Candidate genes were identified and qPCR primer/probe sets selected using the Roche Universal Probe Library Assay Design Center ProbeFinder V 2.49 (https://qpcr.probefinder.com/ organism.jsp) to human homologues (design options for from *M*. *fascicularis* gene sequences are not an available option using this tool). These were compared to the *M*. *mulatta* genome sequence using the BLAST algorithm ([57] http://blast.ncbi.nlm.nih.gov/Blast.cgi). Identified mismatches in primer sequences between human and *M*. *mulatta* gene homologues were then corrected to the Macaque sequence. If a suitable assay could not be generated from the human gene sequence then the *M*. *mulatta* gene sequence was used directly using the raw sequence input option. All qPCR primer sequences were confirmed for Macaque-specificity against database sequences using the BLAST comparison tool, prior to use. Oligonucleotide primers were synthesised by SigmaAldrich (http://www.sigmaaldrich.com) and resuspended in RNAase free water at a concentration of 100 μM prior to use. A total 342 genes of interest (GOI) were selected for further validation (significant gene entity features from microarray analyses are given in Table A S1 File– termedvalidation set (VS)), which comprise (a) 234 genes showing significant changes in expression compared to prebleed controls from microarray analyses (T234), (b) 113 additional genes of immune significance (T113) (c) 3 housekeeping genes for use in data normalisation, PGK1, RPL32 and RPL13A.

#### 2.6.2. Amplification, Cloning and sequencing of Non-human Primate House Keeping Gene Controls

A PCR product for the *M*. *fasicularis* phosphoglycerate kinase 1 gene (PGK1) was synthesised by endpoint PCR in a final volume of 25 μl containing: 5 μl 5x Green GoTaq Reaction Buffer (with MgCl_2_ at 1.5 mM); 2.5 μl dNTPs (dATP, dGTP, dCTP and dTTP each at 2 mM), 0.25 μl GoTaq DNA Polymerase (5 U/μl), 2 μl total upstream plus downstream primer mix (10 μM each primer) and 2 μl unlabelled *M*. *fasicularis* cDNA, using the following PCR protocol: preheat for one cycle at 95°C for 5 min; amplification for 40 cycles: 95°C for 10 seconds, 60°C for 30 seconds, 72°C for 30 seconds and elongation for one cycle at 72°C. Gel electrophoresis was used to assess whether products of the correct size were generated. These were then purified using the Qiagen MinElute PCR Purification Kit, followed by cloning into pGEM-T easy vector and transformation into One Shot TOP10 Chemically Competent *Escherichia coli* according to the manufacturer’s protocols. Blue-white colony screening was carried out where pure white colonies were selected for Mini- or Maxipreps (Qiagen) which was also performed according to the manufacturer’s protocols. Gene inserts were sequenced using BigDye Terminator v3.1 Cycle Sequencing Kit (applied biosciences) and purified using the DyeEx 2.0 Spin Kit (Qiagen) as instructed by the manufacturer. The sequencing reaction products were analysed on ABI PRISM 3130xl DNA Sequencer and the sequence confirmed by BLAST analysis against the *M*. *mulatta* genome.

#### 2.6.3. cDNA synthesis

Five μg of mRNA was mixed with 4 μg of random hexanucleotides and incubated at 65°C for 10 minutes, followed by the addition of 14.6 μl reaction mix, consisting of 6 μl 5x First strand buffer, 3 μl 0.1 M dithiothreitol, 0.6 μl dNTPs (25 mM dATP, dGTP and dTTP and dCTP, Amersham, Buckinghamshire, UK) and 2 μl Superscript II (200 U/μl). The reaction mix was incubated at 42°C for a further 60 minutes, after which an additional aliquot of 1 μl Superscript II (200 U/μl) was added and incubation continued at 42°C for 60 minutes. Any remaining mRNA was degraded by the addition of 15 μl 0.1M NaOH at 70°C for 10 minutes, followed by neutralization with 15 μl of 0.1M HCl. Once the labelling was completed each reaction was purified using the Qiagen MinElute PCR Purification Kit and eluted into 20 μl of nuclease-free water. The mRNA target concentration and specific activity was then determined by spectrophotometry using a NanoDrop ND-1000 spectrophotometer.

#### 2.6.4. Real-time PCR assays using the Roche Lightcycler 480

Real-time PCR assays for each target gene of interest (given in Table A S1 File) were performed in duplicate in 384 well plate format, using the Roche Lightcycler 480 (LC480). Each reaction contained 10 μl Roche Probe mix 1 μl of primer mix (10 μM each primer), 0.5 μl and 3 μl (5 ng/μl) mRNA in a final volume of 20 μl. The following cycling conditions were used; preheat for 1 cycle at 95°C for 10 minutes; amplification for 45 cycles: 95°C for 10 seconds, 60°C for 30 seconds, 72°C for 1 second; and final cooling to 40°C. All the assays were grouped to on to a 384 well plate as singlet reactions and each sample was assayed in triplicate. The PGK1 pGEM-T easy vector clone was used for precise quantification. The plasmid was diluted to an appropriate concentration in nuclease-free water to span approximately 20 qPCR cycles, to create a standard curve which was then saved in the LC480 software. The middle dilution from this standard curve was used as a calibrator on each plate and allowed the software to refer back to the original standard curve dilution series.

#### 2.6.5. Real-time PCR assay Data Analysis using LinRegPCR RT-PCR Analysis Tool

In order to account for variability in PCR efficiencies, non-baseline corrected data were imported into the LinRegPCR program for the analysis of quantitative RT-PCR data ([58,59] http://www.hartfaalcentrum.nl/index.php?main=files&fileName=LinRegPCR.zip&description=LinRegPCR:%20qPCR%20data%20analysis&sub=LinRegPCR). LinRegPCR estimates baseline fluorescence by reconstructing the log-linear phase downward from the early plateau phase of a PCR reaction. PCR efficiency values were calculated per sample, by fitting a linear regression line to a subset of data points in the log-linear phase. Mean PCR efficiencies per amplicon group were used to calculate an estimate of sample starting concentrations. These data were normalised to the ratio of the mean expression values of the calibrator PGK1 and two housekeeping genes (60S ribosomal protein L32 (RPL32), and 60S ribosomal protein L13a (RPL13A), using Microsoft Excel.

#### 2.6.6. Visualisation of qPCR Data Outputs using GeneSpring 12.5

Normalised data were imported into GeneSpring 12.5 (GX 12.5), using baseline transformation to the global median of all samples prior to further statistical analysis and visualisation. All normalised qPCR and microarray data were assessed for quality, then filtered on gene expression on normalised expression using default cut offs. Statistically significant features were identified using one-way ANOVA analysis across all entities and time-points at a cut-off of p ≤ 0.05, with no multiple correction. Statistically significant, shared qPCR and microarray features were identified and analysed further using various functions in GX 12.5 i.e. heat map, Venn Diagram etc. using default settings.

## Results

### 3.1. Hybridisation of Non-Human Primate mRNA to Operon Human Genome AROS V4.0 Microarrays

Cy3-labelled *M*. *fascicularis* mRNAs were hybridised to human Operon Human Genome AROS V4.0 microarray slides and good fluorescence intensity of binding signals were observed. This demonstrated sufficient sequence similarity between *M*. *fascicularis* sequences and the human genes on the array. However to mitigate false reporting, all gene expression analyses for non-human primate mRNAs were conducted with reference to pre-bleed samples only i.e. no unreferenced, non-temporal assessment of gene expression was investigated.

#### 3.1.1. Analysis of Differential Gene Expression Profiles in Non-Human Primate Peripheral Blood Leukocytes in Response to Pulmonary Challenge with *M*. *tuberculosis* Bacilli; Identification of Statistically Significant Differentially Regulated Features

Hybridisation of Cy3-labelled mRNA targets from all NHP PBL samples, across all time-points when filtered on expression, generated a differential gene expression entity list containing all 35352 individual array features. Analysis of Variance (ANOVA) using BH-FDR multiple testing correction at a cut-off of p ≤ 0.05 revealed a large number of differentially regulated features (n = 24488, termed T24488 dataset) compared to baseline pre-bleed expression. This represents approximately 69.3% of all features. Analysis of Variance (ANOVA) using B-FWER multiple testing correction at a cut-off of p ≤ 0.05 revealed fewer differentially regulated features (n = 4506, termed T4509 dataset), representing approximately 12.5% of all features. Further fold-change analysis on these select statistically-significant features (ANOVA B-FWER, Fold Change ≥ 2.0) compared with the pre-bleed condition, revealed 478 features (approximately 1.35% of all features, given in order of expression by week given in Table B S1 File—termedT478 dataset). This represents 472 discrete gene entities.

#### 3.1.2. Cluster Analysis of Statistically Significant Differentially Regulated Features

**Fig 1:**
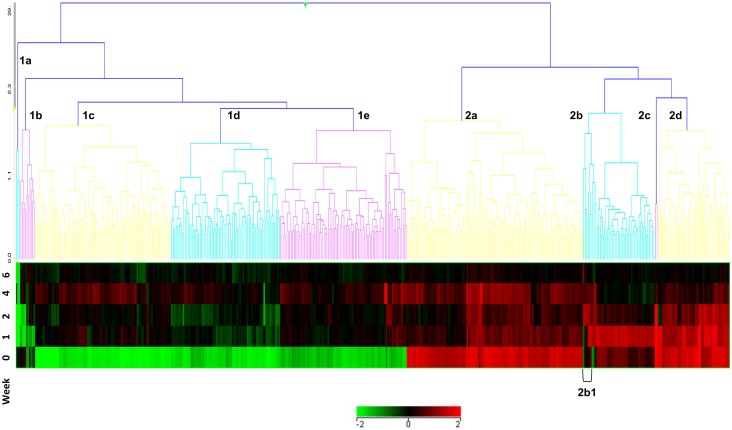
Cluster analysis of temporally expressed entities in peripheral blood leukocytes of Cynomolgus Macaques (all animals) pre-(week 0) or post (weeks 1–6) aerosol-challenge with *M*. *tuberculosis*. These exhibit patterns of up- (cluster 2) or down-regulation (cluster 1) across the six week time course of the experiment. Cluster 2b1 (highlighted) contains co-expressed entities, FOS, IL8 and KLF2.

Unsupervised Euclidean cluster analysis was conducted on the T478 feature set (on entities using default settings and using averaged data across all animals at each time-point). This showed nine clusters of temporally expressed entities (Fig 1 and given in cluster expression order in Table C S1 File), broadly separated into two major clusters, based on down- (five clusters 1a-1e) or up- regulation (four clusters; 2a-2d) with respect to the pre-bleed control. These analyses indicate that changes in gene expression can be detected in circulating peripheral blood leukocytes, distal to the primary site of infection, subsequent to pulmonary challenge with *M*. *tuberculosis*. The entities exhibit patterns of up (cluster 2) or down-regulation (cluster 1) across the six week time course of the experiment.

**Fig 2:**
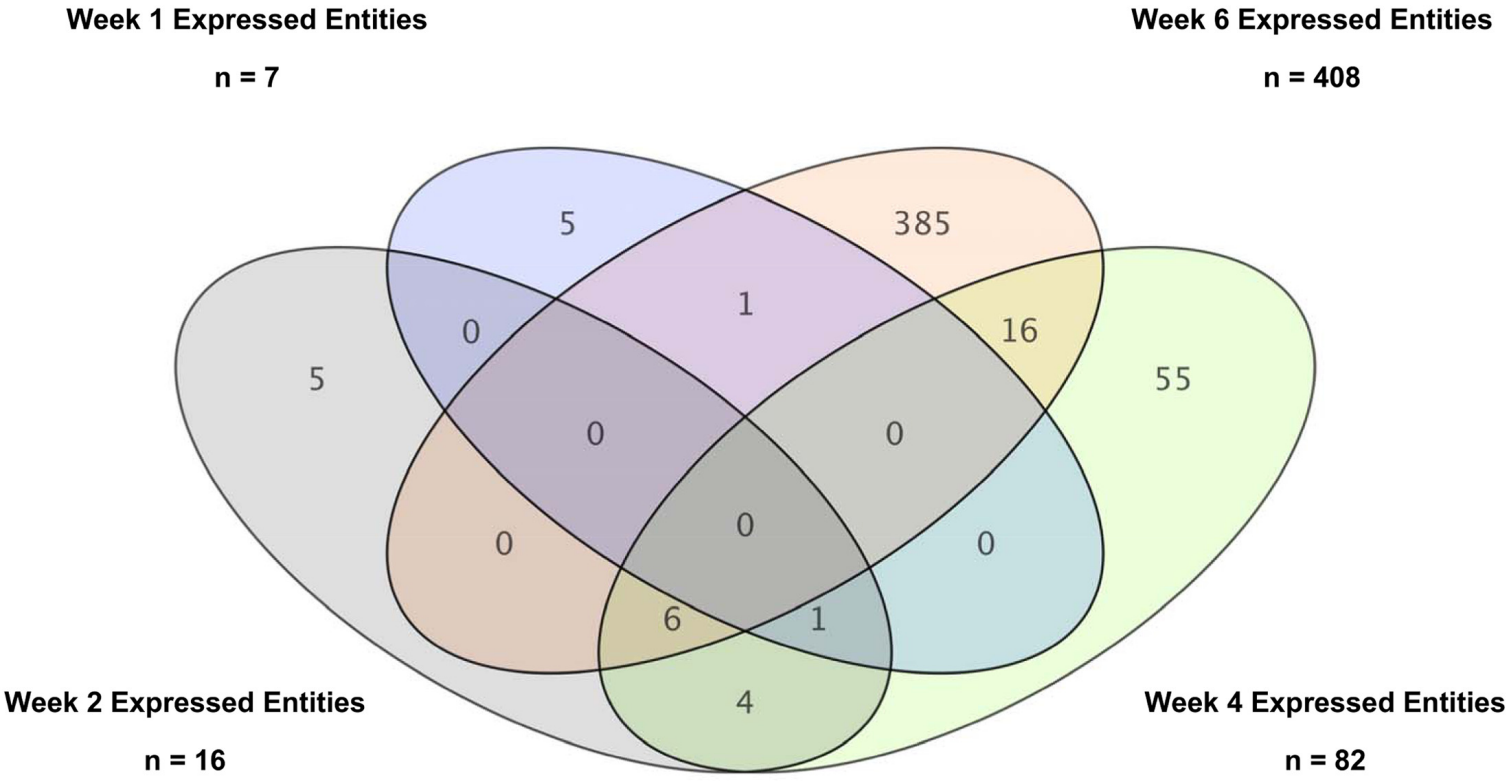
Venn Diagram Comparison between Entities Temporarily Expressed at Different Weekly Timepoints from the T478 feature set.

A relatively small number of features were found to be differentially regulated at weeks one (eight up-regulated) and two (13 up-regulated and six down-regulated). However, the number of differentially regulated genes increased significantly at weeks four (70 up-regulated and 12.5 down-regulated) and six (162 up-regulated and 249 down-regulated) of the study. Venn diagram comparison analysis of the differentially regulated entities across weekly time-points revealed no common entities; however some features are shared between one or more time-point (see Fig 2). All entities exhibited shifting temporal patterns of regulation as the time-course of the infection progressed. These correlate with increasing symptoms and signs of clinical disease, including fever, weight loss and other adverse indicators. The results show that there is a moderate response in PBLs to pulmonary challenge with live tubercle bacilli at the early week time-points one and two. However, a more pronounced response is observed from around four to six weeks post-infection, which correlates with an increase in the number of differentially expressed entities. These include genes involved in normal cellular biochemical processes and also immune inflammatory mediators, among others.

#### 3.1.3. Pathway and Similar Entity Analysis of Statistically Significant Differentially Regulated Features and Identification of Temporal Profile Expression Profiles of Immune Inflammatory Markers

Temporal differential regulation of gene features appeared to be correlated with immune activation. To investigate this further, pathway analyses were conducted on each cluster to identify significant features. Statistically significant pathways identified for each cluster were prepared and pathways with a p-value of significance below ≤ 0.05 were selected (given in Tables A–I S2 File). No identified pathway exhibited a complete gene entity set, most contained around one or two entity matches. Many entities were shared between the listed statistically significant pathways, however there appeared to be significant cluster-specific enrichment of entities e.g. type II IFN signalling in clusters 2c and 2d. The preeminent, identified statistically-significant pathways were (1a) (1b) (1c) (1d) (1e) (2a) (2b) IL3 signalling and (2c/2d) type II interferon signalling. The genes associated with this latter pathway include IRF1, IFNGR1, JAK2 and GBP1.

Using the similar entities function of GX 12.5 (above a correlation co-efficient similarity threshold cut-off of 0.9) (1) IRF1 expression correlated with seven gene features, which included PSMB9, LGALS3BP, RNASE6, CD93, IFI44 and CARD6, (2) IFNGR1 with two gene features SERPINB1 and CREG1, (3) JAK2 with seven gene features RP4-681N20.1, GABARAP, PSTPIP2, SLC40A1, RNF24, SH3GLB1 and CFLAR and (4) GBP1 did not associate with any other markers above this cut-off threshold, but associated with PLAC8 and JAK2 at a lower cut-off of 0.7. CD93 is mainly a myeloid cell marker; therefore the IRF1 associated response may be associated with cells of myeloid origin. However, the other genes associated differently, which may perhaps indicate expression in alternate cell populations.

**Fig 3:**
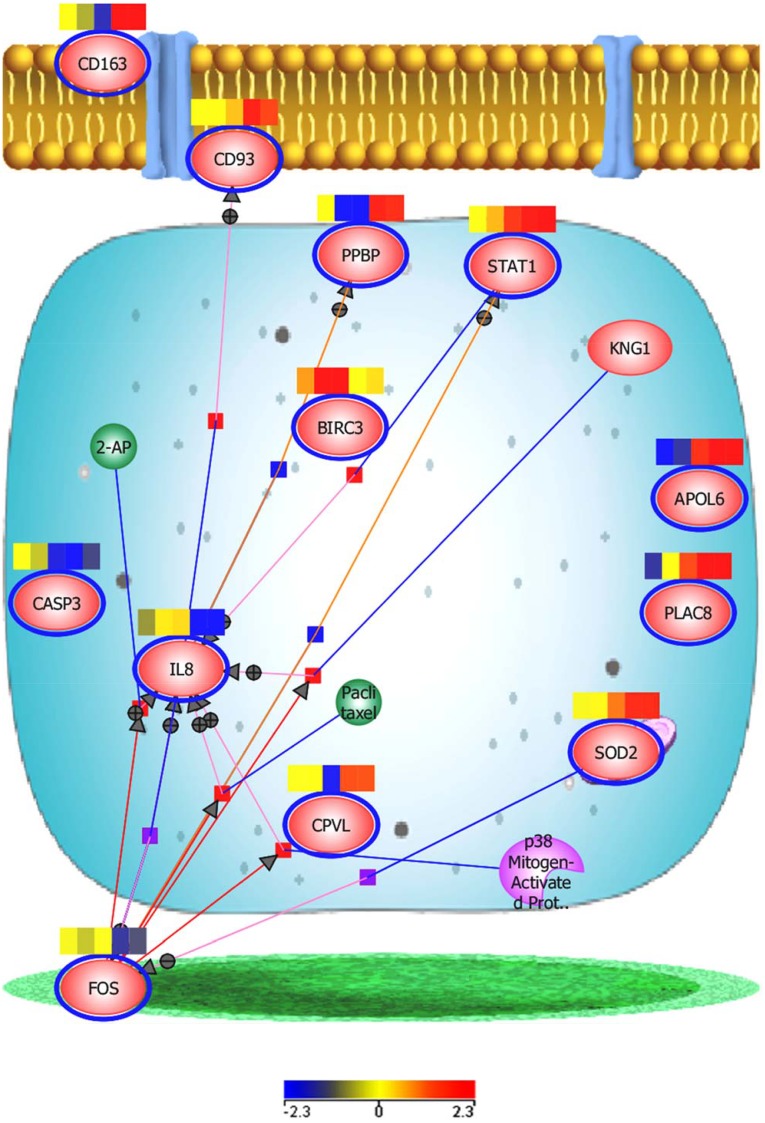
Cellular pathway map of key regulatory entities at the two to four week time-points. Heatmaps for each entities showing expression of key entities from microarray analyses across all animals in the study.

FOS and IL8 appear differentially up-regulated until around the week two time-point in all six animals and were then down-regulated. Using the similar entities function of (GX12.5), FOS expression appeared to be intimately associated with that of another entity, KLF2 (significance value 0.904) and more weakly with IL8 (significance value 0.732). These are part of a unique cluster of genes in cluster 2b, highlighted as 2b1 (shown in Fig 1 and given in Table C S1 File) which appear to be down-regulated as Type II interferon-type responses become more evident at weeks two to four. There may be an inverse correlation between an early FOS-directed response, with that of an interferon-driven response between weeks two to four onward (see Fig 3). FOS-related entity expression may be a marker of very early post-exposure immune responses, whereas transition to an IFN-related response may be indicative of progression to more active disease.

Data from this analysis were compared with that from a previously published study by Gautam et al [60]. A genelist of differentially expressed entities between Rh-BMDMs infected with Mtb-H or Mtb-A identified by DNA microarray was imported into GX 12.5 and compared with the T4509 dataset. Four hundred and eight gene entities were found to be shared between the features identified in this prior publication and our T4509 dataset (given in Tables D–F S1 File). This includes interestingly, many of the entities listed above e.g. FOS, IL8, GBP1, IRF1, KLF2, and CREG1, among others. However, due to differences in the composition of the different cell populations between the studies i.e. Rh-BMDMs in the Gautam study or the more heterogeneous and complex total PBLs used in this study, it would perhaps be expected that the shared gene entity list would comprise features more specifically expressed in monocyte/ macrophage lineage cells only. This does not preclude their expression in other cell types as some entities are expressed in other cell lineages also e.g. GBP1. However this comparative analysis may reveal that the observed step-change in total gene expression in our study could be underpinned primarily by an altered monocyte/macrophage-associated response.

Differentially-regulated entities were selected from these analyses for further investigation, e.g. CASP3, CRP, FOS, IL8, and SOD2, including those previously identified for type II interferon signalling. Other immunologically relevant features were also included and with entities considered biologically relevant and worth further detailed investigation e.g. apoptosis related genes.

#### 3.1.4. Further Analysis of Differentially Regulated Features Associated with Type II Interferon Responses

NHPs from the two separate breeding colonies i.e. of Mauritian (MN) or Chinese (CN) origin, from established United Kingdom (Mauritian lineage animals) or Chinese breeding facilities used in this study were found to have differing susceptibilities to infection with *M*. *tuberculosis*. Animals of MN origin were innately more susceptible to infection, had lost greater than 10% body weight by week six and had to be humanely euthanized earlier in the study than the CN animals. Animals from the CN breeding colony were more resistant and survived to week 12 of the study, after which they too succumbed to severe infection and were humanely euthanized. These differences may reflect innate differences in response between the two groups. Gene entities indicative of developing Type II interferon responses were highlighted as highly statistically significant in the previous section (3.1.3) and these overall showed common profiles in both groups, with some temporal expression differences.

Type I and II Interferon responses have also been highlighted in previous human studies as being particularly significant in the ongoing immunological response to Tuberculosis infection (reviewed in [61]). We therefore sought to further analyse the responses of individual animals in the study to ascertain whether entities in these pathways were differentially regulated across time-points, but also by origin and whether these could be correlated with susceptibility, development of symptoms and disease progression. An entity list for the Type II Interferon pathway was imported into Genespring using the ‘Import Entity list from file’ function (http://www.wikipathways.org/index.php/Pathway:WP619). Two-way ANOVA analyses (BH-FDR multiple testing correction, p ≤ 0.05) and fold -change analysis (≥ 1.5), of these selected entities across all animals and time-points revealed 33 statistically significant features (given in Table G S1 File). These include in order of significance, SOCS3, JAK2, IFNGR1, EIF2AK2, IRF2, OAS1, STAT1, IRF4, IRF1 and IFNβ1.

**Fig 4:**
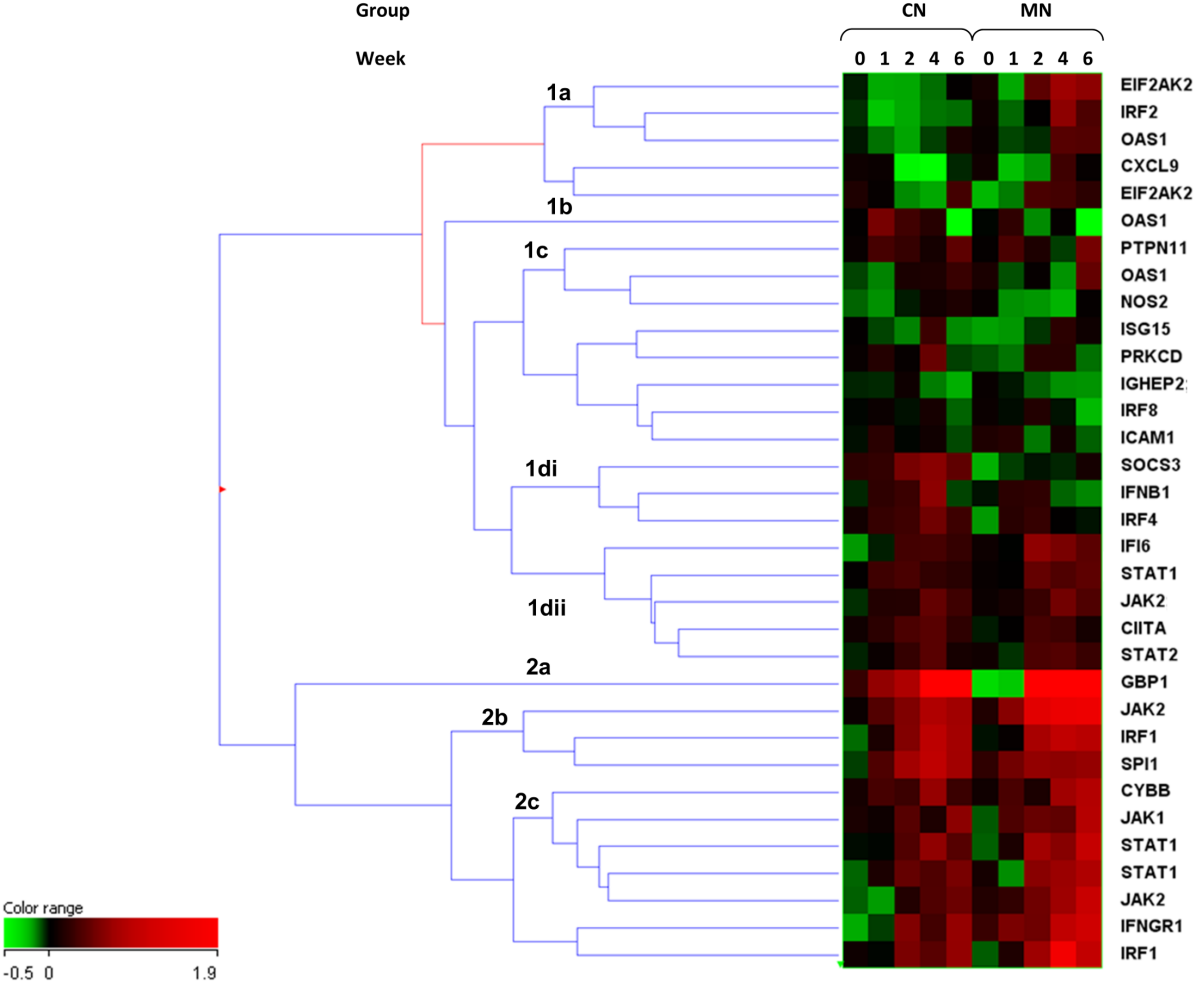
Cluster analysis of Type II Interferon-related entities in NHPs of Chinese or Mauritian origin.

Unsupervised Euclidean hierarchical cluster analysis across all animals and time-points (averaged group data, segregated and analysed by origin) on this latter dataset is shown in Fig 4. The data segregate into two main clusters, cluster 1 differentiates into four broad sub-clusters and cluster 2 into three sub-clusters. Many of the features represented in cluster 2 and 1dii exhibited broadly similar expression patterns across all animals and time-points. However there are some differences in temporal response, particularly with gene features in clusters 1a-c and 1di. These appear to correspond to the origin of the animals in the study (which exhibit highly differing innate susceptibilities to infection with Tubercle bacilli). The animals of Chinese origin express a higher level of SOCS3, IFNB1 and IRF4 among other features out to the six week time-point. The animals of Mauritian origin exhibit a similar degree of expression of IFNβ1 and IRF4 at weeks one and two, these are then down-regulated at weeks four and six, while SOCS3 expression was reduced at every time-point in this group. This appeared to correspond with increased overt symptoms. The MN animals also expressed a higher level of expression of EIF2AK2 from the week two time-point then the CN animals and `IRF2 and OAS1 at weeks four and six, which again corresponded with increasing overt signs of disease. Down-regulation of SOCS3, IRF4 and IFNβ1 appeared to correlate with increasing signs of disease, then up-regulation of IRF2 with disease severity in these animals prior to euthanasia.

### 3.2. Validation of Differentially Regulated and Other Immunologically Relevant Entities using qPCR

Of the statistically significant hits from ANOVA analyses (section 3.1.1), 347 entities were selected for further analysis (all entities given in Table A S1 File –termed validation set (VS)); these comprosed 234 entities (termed T234 entity list) from the microarray ANOVA analyses (T24488) and 113 immunologically-relevant select entities from other sources (termed T113 entity list). Validation of these and a selection of other relevant entities were conducted using the Roche LightCycler 480 real-time PCR system as described above. qPCR raw data outputs were normalised to the average of control and calibrator gene signals prior to importation (using no further normalisation transformation), using the Baseline transformation function of GX12.5. Further analyses and visualisation were then conducted using various other functions of this software package. Fold-change analysis on averaged data across on all 347 entities, group and week (FC ≥ 1.5) revealed 223 differentially regulated entities across all time-points compared with the pre-bleed. All data are given in Table H S1 File (ranked on p value of the difference between the animals of MN or CN origin in the pre-bleed control data column).

#### 3.2.1. Validation of Statistically-Significant Entities from Microarray ANOVA Analyses

Fold-change analysis was performed on the T234 entity list qPCR data, using the cut-off ≥ 1.5 (settings; averaged data, grouped on week and animal origin and compared with the pre-bleed control), 153 entities were detected (65.39%). ANOVA analyses (p ≤ 0.05, no multiple testing correction on datasets, grouped on week and group) revealed 51 statistically significant entities (21.8%) the most highly significant being CD163, GBP6, GBP1, FOS, BIRC3, FAS, IL11, FZD2, CD7, TNFSF10 and FAM96B. This is in contrast to the results obtained for microarray hybridisation analysis, where all T234 entities were found to be highly statistically significant (p ≤ 0.05). These entities again showed clear temporal expression profiles over the course of the study from week zero (pre-bleed) to week six, with a number of entities appearing to be particularly differentially regulated at the four and six week time-points. FOS is again of particular interest as the validation data confirmed the microarray observations that this transcriptional regulator is up-regulated until week two, after which it is down-regulated at weeks four and six. This is coincident with up-regulation of interferon-regulated and other entities e.g. CD163, GBP6, GBP1 and others e.g. IL8, IL7R, CD3E, BIRC3, PMAIP1, CD74 (CLIP), CD40 Ligand, TNFRSF10A, CCR9, CXCR4, CCR7. An increase in expression of interferon-regulated entities again became increasingly apparent over the four and six week time-points of the study, with increased expression of IRF1, IRF3 and STAT1 among others.

#### 3.2.2. Validation of Other Immunologically-Relevant Entities not from Microarray Derived Entity Lists

Fold-change analysis was performed on the T113 entity list qPCR data, using the cut off ≥ 1.5 (settings; averaged data,) grouped on week and group and compared with the pre-bleed, detecting 70 entities (61.95%). These entities also showed clear temporal expression profiles over the course of the study from week zero (pre-bleed) to week six, although they were not identified as statistically significant entities in the previous microarray hybridisation analyses. ANOVA analyses (p ≤ 0.05, no multiple testing correction on datasets grouped on week and group) revealed 21 statistically significant entities (18.58%), the most highly significant being FCGR1B, IL18R, IFIT3, CASP4, APOL6, JUN, CASP9, CLEC4E, CD2, MIF, CD8α and CD8β. These are important entities in development of the adaptive immune response; therefore validation of these entities provides valuable additional information with regard to the immune pathways involved in temporal disease development.

The most statistically-significant, differentially regulated features across all animals and time-points are given in Table 1. These combined results provide evidence of a step shift between the innate and adaptive immune responses, i.e. suppression of select gene expression elements in key cellular immune response pathways with concurrent up-regulation of other responses. There is evidence of two phases of infection from an ‘early’ FOS-linked response to a ‘late’ type II IFN-linked response. However, it is inferred that an increase or decrease in transcript abundance is due to differential transcriptional regulation. However, the results could equally be interpreted as a reflection of cell death/loss i.e. apoptosis/necrosis of cells or egress of key cell types from the periphery, perhaps to the primary site of infection.

**Table 1:** Fold change values of the most highly statistically-significant, differentially regulated qPCR validated entities.

Gene Symbol	Gene Name	FC W1 vs W0	Reg	FC W2 vs W0	Reg	FC W4 vs W0	Reg	FC W6 vs W0	Reg
FOS	FBJ murine osteosarcoma viral oncogene homolog	-1.0178504	**down**	1.5105207	**up**	-5.998902	**down**	1.175655	**up**
IL7R	interleukin 7 receptor	1.5602038	**up**	1.1026541	**up**	-4.4693823	**down**	-1.5704274	**down**
FCGR1B	Fc fragment of IgG, high affinity Ib, receptor (CD64)	-1	**down**	1.2304243	**up**	8.440779	**up**	24.315327	**up**
IFIT3	interferon-induced protein with tetratricopeptide repeats 3	1.193859	**up**	6.577363	**up**	13.944085	**up**	2.7974696	**up**
GBP6	guanylate binding protein family, member 6	1.2704407	**up**	5.644048	**up**	8.7505665	**up**	8.209202	**up**
GBP1	guanylate binding protein 1, interferon-inducible	-1.1683992	**down**	3.7988372	**up**	4.3289824	**up**	11.418418	**up**
APOL6	apolipoprotein L, 6	-1.1741112	**down**	4.3224673	**up**	5.7248235	**up**	10.907694	**up**
CASP4	caspase 4, apoptosis-related cysteine peptidase	1.0721039	**up**	1.0027341	**up**	5.7921696	**up**	5.4235997	**up**
CD163	CD163 molecule	-1.6392189	**down**	-2.2243066	**down**	8.829087	**up**	4.6299896	**up**
TNFSF10	tumor necrosis factor (ligand) superfamily, member 10	-1.2342447	**down**	-1.0099833	**down**	2.474039	**up**	1.8384712	**up**
CCL23	chemokine (C-C motif) ligand 23	-1	**down**	-1	**down**	1.3849256	**up**	1.4014934	**up**
PLAC8	placenta-specific 8	2.1797173	**up**	2.3677406	**up**	5.0824566	**up**	9.323483	**up**
FAS	Fas (TNF receptor superfamily, member 6)	2.3143773	**up**	3.0103607	**up**	3.2973375	**up**	6.2040267	**up**

#### 3.2.3. Comparison of anti-Tuberculosis Immune Responses in Macaques from Different Lineages

Further analysis of the 72 statistically significant entities from sections 3.2.1 and 3.2.2 across all combined time-points and animals using non-averaged data was conducted. This revealed clear differences in expression across time-points but also identified some differences between individual animals. Due to the observed differences in innate sensitivity/resistance between the two groups of animals of different lineages used in the study i.e. MN and CN, ongoing analyses were conducted using data separated into the two groups based on origin. Investigation of inherent differences in response between the two groups was further explored using T-test analysis (unpaired T-test, unequal variance, p ≤ 0.05, fold change cut off ≥ 1.5 on non-averaged data, no multiple testing correction, individuals grouped according to origin) on the 72 statistically significant hits from sections 3.2.1 and 3.2.2 (given in Table I S1 File).

**Fig 5:**
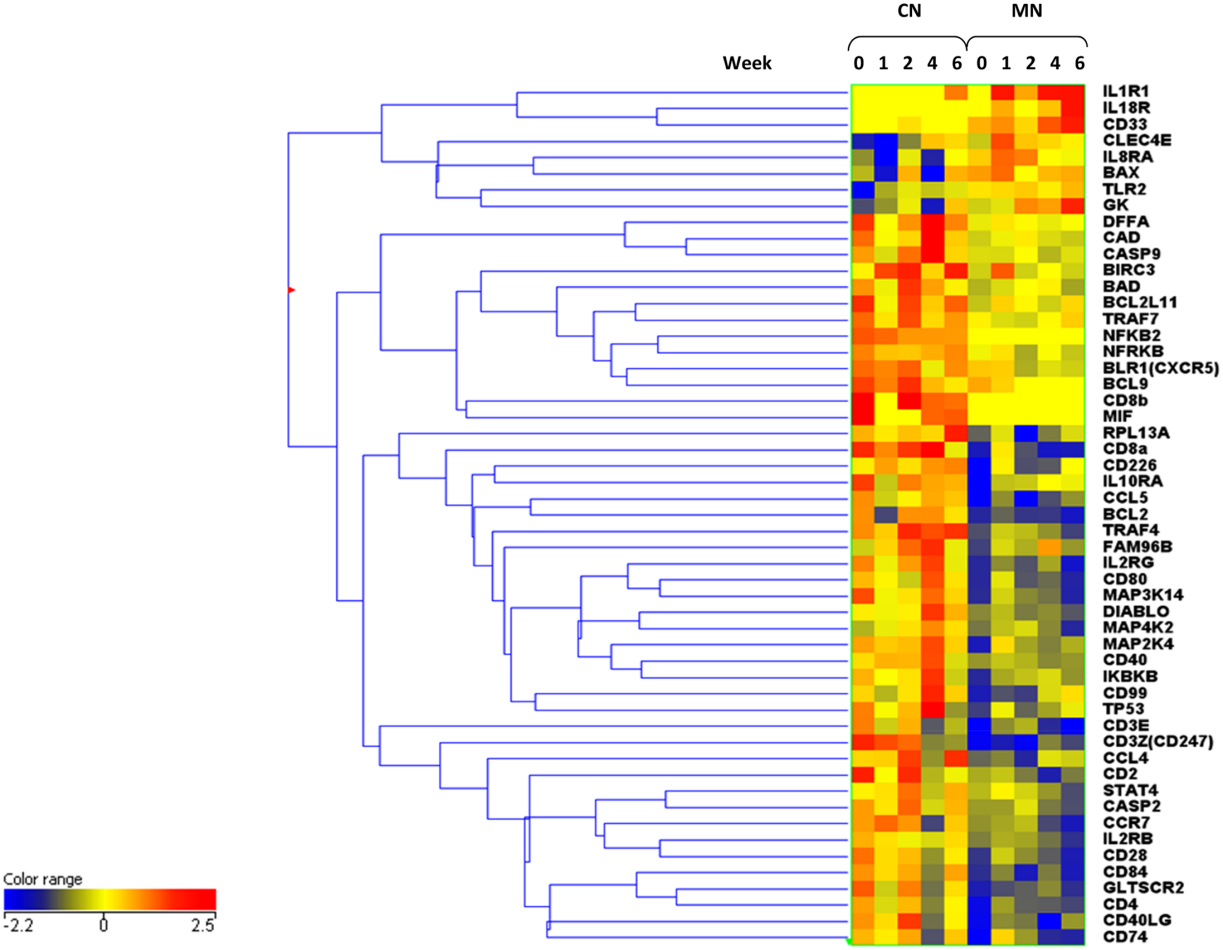
Cluster analysis of statistically significant, validated entities in qPCR datasets; segregated Chinese and Mauritian Cynomolgus Macaque groups.

Fifty-three entities were found to be differentially expressed between the two groups. Eight were found to be up-regulated in the MN compared with the CN lineage animals and 45 up-regulated in the CN compared with the MN lineage animals (Fig 5). Many of these markers again show temporal expression patterns across the time-course of the study. These is clear lineage specific expression of key markers, particularly with regard to T-cell specific markers CD8α and CD8β, CD4, IL2R and also macrophage markers i.e. MIF (macrophage migration inhibitory factor). The Mauritian lineage animals also exhibit high expression of IL1R1, il18Ra and the myeloid marker CD33 across all time-points; this was not seen in the CN lineage animals. Markers associated with T-cell responses appear up-regulated at week four and then down-regulated in the CN animals at week six. CD2, CD4, and IL2RB appear partially restored at week six, but not CD8α, CD3ε and CD3ς and others, which are still down-regulated at week six.

### 3.3. Identification of Significant Entities using Parametric and Non-Parametric Analyses and Comparisons of the Non-Human Primate and Human Datasets

Further analysis of NHP microarray data sets was conducted using artificial neural network algorithms and the network inference approach described above in section 2.5.3. Ranked order lists were produced of NHP data outputs on average test error. The top 100 (T100ANN) and 1000 (T1000ANN) performing features for all entities in the microarray dataset and the top 50 (T50ANN VS) for the validation set were selected for further comparative analysis.

#### 3.3.1. Network Analysis of Statistically Significant Entities from Non-Parametric Analyses of the NHP Tuberculosis Data Set

To determine some of the regulatory networks underpinning the peripheral immune responses in this NHP TB model, the T100ANN data set was analysed using network inference interaction analysis tools. This generated an alternative, parallel view of the underlying host response processes ongoing during infection, in addition to those revealed using parametric analysis tools. The analysis of combined and separated group-specific data for the T100ANN hits across all animals and time-points are given in Figures A–C S3 File. All data outputs were visualised using Cytoscape.

Three high degree, common major nodes (hubs) were identified (Figure A S3 File) across both NHP groups, centralised around genes CDSN, KLHDC3 (negative influence) and HIST1H2BE (positive influence), with four minor nodes centralised around genes POLR2J3, calcium binding protein 22/ calcineurin homologous protein (AC012.565_1), IL15 (all positive influence) and CLK1 (negative influence). All these markers were moderately up-regulated from the week 1 time-point onwards in both NHP groups. It is unclear how some of these markers exert their regulatory effects e.g. KLHDC3 (kelch domain containing 3) and CDSN (corneodesmosin), however HIST1H2BE and Il15 are of interest as the former is involved in the innate response to gram-positive bacteria and the latter regulation of T and NK cell activation and proliferation and may increase the expression of apoptosis inhibitor BCL2L1/BCL-x(L), possibly through the transcription activation activity of STAT6, and thus prevent apoptosis. NHP group-specific pathway interaction maps are given in Figures B and C S3 File. These show that although there are some commonalities in response shared between the groups, each of the groups show a unique profile when the data are analysed separately.

**Fig 6:**
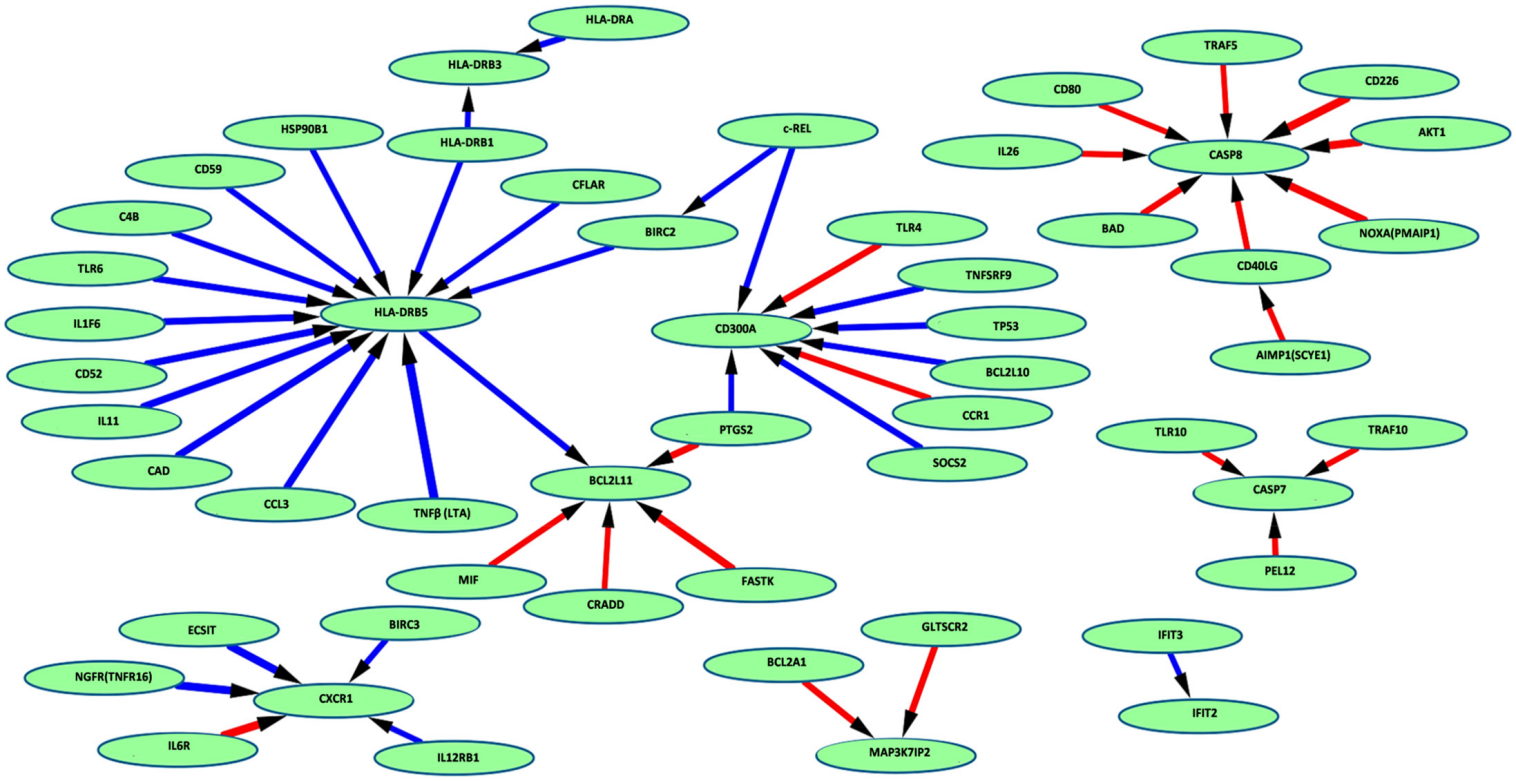
Network inference map results from the T50 VS dataset across both CN and MN NHP groups, visualised using Cytoscape. Blue arrows indicate negative influence effects and red arrows positive regulatory effects of increasing intensity represented by the thickness of the line.

Further detailed analysis was conducted using the T50ANN VS biomarker entity set to extend our understanding of some of the less overt gene common interactions at play (Fig 6) and also between the NHP groups of different origins (Figures D & E S3 File). Complex profiles of interactions were observed for all entities across all animals and specific profiles of animals segregated according to origin. However, common features across all animals were positive influence of HLA-class II molecules HLA-DRB5, DRB1, DRB3 and DRA, negative influence on the pro-apoptotic markers BCL2A1, BCL2L11, CASP8 and CASP7. The CN animals exhibited evidence of negative influence on pro-apoptotic markers e.g. BAD, BIK and BCL2L10. The MN animals appeared to negatively regulate anti-apoptotic markers BCL2L2 and BCL2A1, but also pro-apoptotic markers APOL6 and BAX. They also appeared to exhibit a TLR4-driven response. This was not apparent in the MN animals, which appear to favour a more typical anti-mycobacterial TLR2 and TLR6-type response.

These analyses delineate markers perhaps not identified using parametric tools, which may represent some of the subtler responses at play in this infection model. These combined analyses have revealed an overall view of profound positive influences on pro-inflammatory events. This supports the data presented in the previous sections to suggest that overall up-regulation of pro-inflammatory events may be a feature of ongoing TB infection in peripheral cells. Variable regulation of anti/pro-apoptotic entities, specific according to group origin was also observed. However, as discussed in previous sections, this is not productive as part of an anti-mycobacterial response, due to perhaps concurrent dysregulation of T-cell responses.

#### 3.3.2. Comparison of Statistically Significant Entities from Parametric and Non-Parametric Analyses of the NHP Tuberculosis Data Set

Data from ANOVA using BH-FDR multiple testing correction at a cut-off of p ≤ 0.05 were ranked according to p value from lowest to highest and the top performing features (T1000ANOVA) selected for further comparative analysis with those from the T1000ANN dataset.

The T1000ANOVA and T1000ANN entity lists were compared using the Venn diagram comparison function of GeneSpring v 12.5. Shared features were identified from these analyses (n = 222, corresponding to 218 discrete gene entities, Figure A S4 File). Cluster analysis of these entities revealed segregation of these entities into two asymmetrical clusters (Figure B and listed in cluster order in Table A S4 File), down-regulated entities (n = 10) and up-regulated entities (n = 212). There is therefore significant enrichment for features which exhibit up-regulation, using this comparative analysis method with the data in this study. These results show that analyses using different parametric and non-parametric methods generate different profiles, as only 22.2% are shared in the top ranked 1000 between the datasets. Comparing the datasets provides valuable information of consensus entities, which may be of improved value for further development.

#### 3.3.3. Identification of Statistically Significant Entities from Comparison of NHP and Human Tuberculosis Data Sets

To further assist in delineation of PBL-derived disease-relevant entities in both primate and human Tuberculosis infection, statistically significant entity lists from ANOVA analysis of the NHP expression data and from two human previously published human data sets were compared. Statistically significant entities from this NHP-TB study (n = 24488) and from human data sets GSE19439 (n = 2585) and GSE28623 (n = 112.520), were identified using ANOVA (using BH-FDR p ≤ 0.05). These human entity lists were then imported into GX 12.5, and compared with the NHP entity list the using the Venn diagram comparison function tool. Shared disease-relevant features were identified (n = 1148), corresponding to 843 discrete gene entities which were selected for further comparative analyses.

#### 3.3.4. Identification of Biomarker Candidates from Combined NHP parametric and non-parametric and Human Gene Lists

Gene entity lists from the above NHP parametric and non-parametric comparison dataset analyses (n = 222) and from comparison with NHP and human parametric ANOVA analyses (n = 1148) were further compared using the Venn diagram comparison function of GeneSpring v 12.5. Thirty-one features corresponding to 30 discrete gene entities were found to be shared between the two data sets (Table 2). These are ranked on composite corrected p value across all three studies, from lowest to highest p value as a measure of overall significance. All 30 biomarkers were found to be associated with the active TB group in both human studies (Figs A and B S5 File) and are up-regulated in all datasets, compared with controls. This comparison method may be useful for selection of preferred, minimal biomarker sub-sets.

**Table 2:** Gene entities from comparison of NHP and human parametric ANOVA analyses. Thirty-one features corresponding to 30 discrete gene entities were found to be shared between Cynomolgus Macaque and two human data sets. These are ranked from lowest to highest using composite p value.

			NHP-TB		GSE19439		GSE28623		
GENE SYMBOL	GENE NAME	ENTREZ ID	p value corrected (BH-FDR)	p value	p value corrected (BH-FDR)	p value	p value corrected (BH-FDR)	p value	Composite Corrected p value
GBP1	Interferon-induced guanylate-binding protein 1	2633	2.46E-21	1.59E-22	6.11E-10	1.19E-10	2.49E-11	1.95E-12	2.12E-10
SAMD9L	Sterile alpha motif domain-containing protein 9-like	219285	9.30E-12	4.80E-12	3.49E-10	1.94E-11	2.09E-09	4.83E-10	8.16E-10
JAK2	Tyrosine-protein kinase JAK2	3717	1.41E-11	9.88E-12	6.59E-10	1.73E-10	4.13E-09	1.19E-09	1.60E-09
IRF1	Interferon regulatory factor 1	3659	6.22E-22	2.00E-23	3.46E-10	8.24E-12	8.36E-09	2.73E-09	2.90E-09
GBP2	Interferon-induced guanylate-binding protein 2	2634	5.24E-11	4.58E-11	5.09E-09	1.57E-09	1.02E-07	4.32E-08	3.58E-08
LAP3	Cytosol aminopeptidase	51056	8.05E-10	8.05E-10	6.11E-10	1.39E-10	1.73E-07	7.65E-08	5.81E-08
STAT1	Signal transducer and activator of transcription 1-alpha/beta	6772	2.02E-10	1.89E-10	6.11E-10	1.22E-10	6.68E-07	3.43E-07	2.23E-07
PARP9	Poly [ADP-ribose] polymerase 9	83666	2.98E-14	5.78E-15	1.88E-06	8.06E-07	1.44E-09	2.76E-10	6.27E-07
TRIM25	Tripartite motif-containing protein 25	7706	4.79E-11	4.02E-11	1.97E-06	8.90E-07	3.84E-09	1.03E-09	6.57E-07
BST1	ADP-ribosyl cyclase 2 Precursor	683	2.98E-11	2.32E-11	7.01E-06	3.84E-06	1.05E-09	1.62E-10	2.34E-06
CALCOCO2	Calcium-binding and coiled-coil domain-containing protein 2	10241	1.29E-11	7.93E-12	2.98E-05	1.92E-05	2.86E-07	1.32E-07	1.00E-05
BAZ1A	Bromodomain adjacent to zinc finger domain protein 1A	11177	1.22E-11	7.09E-12	3.44E-05	2.29E-05	8.01E-09	2.46E-09	1.15E-05
EIF4E3	Eukaryotic translation initiation factor 4E type 3	317649	2.56E-15	4.12E-16	2.51E-05	1.55E-05	1.53E-05	9.39E-06	1.34E-05
DMXL2	DmX-like protein 2	23312	2.71E-13	8.74E-14	2.98E-09	8.52E-10	9.44E-05	6.36E-05	3.15E-05
LYN	Tyrosine-protein kinase Lyn	4067	2.71E-13	7.98E-14	6.72E-05	5.12E-05	7.60E-05	4.97E-05	4.77E-05
SEMA4A	Semaphorin-4A Precursor	64218	1.35E-11	8.70E-12	9.32E-06	5.33E-06	3.94E-04	2.96E-04	1.35E-04
SNX10	Sorting nexin-10	29887	5.72E-14	1.29E-14	4.95E-04	4.12E-04	5.16E-08	1.99E-08	1.65E-04
CREG1	Protein CREG1 Precursor	8804	1.67E-13	4.30E-14	7.25E-04	6.21E-04	2.73E-10	3.15E-11	2.42E-04
MVP	Major vault protein	9961	2.76E-13	9.78E-14	1.50E-06	5.73E-07	9.73E-04	8.05E-04	3.25E-04
RNF24	RING finger protein 24	11237	2.98E-11	2.41E-11	1.19E-03	1.08E-03	6.47E-07	3.11E-07	3.97E-04
SERPINB1	Leukocyte elastase inhibitor	1992	6.12E-17	5.93E-18	1.20E-06	4.30E-07	2.39E-03	2.02E-03	7.98E-04
FCN1	Ficolin-1 Precursor	2219	1.41E-11	1.00E-11	1.10E-04	8.63E-05	2.53E-03	2.19E-03	8.81E-04
CYBB	Cytochrome b-245 heavy chain	1536	4.75E-12	2.30E-12	2.37E-05	1.41E-05	2.95E-03	2.61E-03	9.90E-04
WAC	WW domain-containing adapter protein with coiled-coil	51322	9.46E-12	5.19E-12	2.60E-03	2.48E-03	3.86E-04	2.82E-04	9.96E-04
PLAC8	Placenta-specific gene 8 protein	51316	1.09E-15	1.40E-16	3.49E-05	2.49E-05	3.69E-03	3.33E-03	1.24E-03
CSAD	Cysteine sulfinic acid decarboxylase	51380	4.62E-13	1.79E-13	5.54E-03	5.41E-03	4.18E-04	3.22E-04	1.99E-03
FYB	FYN-binding protein	2533	4.09E-12	1.71E-12	1.67E-03	1.55E-03	7.81E-03	7.20E-03	3.16E-03
ALPK1	Alpha-protein kinase 1	80216	3.71E-10	3.59E-10	3.92E-06	1.96E-06	1.64E-02	1.58E-02	5.48E-03
KPNB1	Importin subunit beta-1	3837	5.24E-11	4.74E-11	1.77E-06	7.15E-07	2.41E-02	2.37E-02	8.05E-03
ST3GAL4	CMP-N-acetylneuraminate-beta-galactosamide-alpha-2,3-sialyltransferase	6484	2.98E-11	2.21E-11	9.74E-04	8.58E-04	2.63E-02	2.63E-02	9.09E-03

Further investigation using Multiomic pathway analysis using averaged NHP-TB array data and GSE19439, revealed a number of highly significant pathways (p ≤ 0.005, given in Table J S1 File). A number of these share previously identified pathway entities as outlined in Table 2 (i.e. IRF1, JAK2 and FYB) and are important components of the adaptive immune response. As these and other biomarkers from Table 2, are found to be significant across all datasets, i.e. across primate species, they may be particularly useful as diagnostic biomarkers for downstream assay development. A number of these highly significant entities have been selected for further investigation as diagnostic biomarkers of Tuberculosis (UK Patent number 1408100.4).

## Discussion

Differential gene expression profiles were investigated in a non-human primate model of pulmonary Tuberculosis using Operon AROS Human genome whole genome arrays. This heterologous microarray hybridisation approach has been used successfully by previous groups in Rhesus Macaque models of infection [29,31]. Differentially regulated biomarker profiles were referenced to unchallenged pre-bleed samples and biomarkers validated using quantitative real-time PCR where possible to eliminate any technical issues associated with expression profiling. Biomarker profiles were also compared with those identified in a number of different Human studies to establish commonality in the immune response to TB challenge in this model.

A very large number of biomarkers were found to be differentially regulated over the six week course of the study, in comparison to pre-bleed, unchallenged control samples. However, at this present time, it is not known whether these changes are indicative of a) gene expression/ regulatory changes, b) through egress/exodus of cells expressing these markers from the periphery (through recruitment to the site of infection for example), c) cell death through apoptosis or d) necrosis or cell expansion/recruitment. The terms differential gene expression or regulation are thus used in this study to embrace all these possible options, as it is impossible as yet to ascertain which of these is responsible for the observed profile changes. However, there is some indication that this observed effect may in part be explained by depletion of key transcript-expressing cells from the periphery, although this may not be the only underpinning mechanism evident. We also observed differential up-regulation of markers associated with apoptosis, particularly at the four week time-point, prior to a substantial loss of transcripts between this and the six week time-point. This would suggest that after a peak in expression at the four week time-point, cell death through apoptosis could also play a significant part in transcript abundance changes. This may be supported by the observed increase in CD93 receptor abundance, thought to be involved in scavenging of apoptotic cells.

Few statistically significant gene expression changes are observed between the pre-bleed and week one samples. Eight are the most significant (FC ≥ 2.0) UBN1, CLK1, RPL13A, PBX1, EN2, ANPEP and CDH20 (given in Table B in S1 File). Expression of these biomarkers may reflect indicators of the very early responses to infection. All these entities are up-regulated at the week one time-point compared with the control; however the role of some of these e.g. UBN1, CDH20 and RPL13 in disease pathogenesis is unclear, but the cell biology processes of the former two may be linked [62,63] and could be involved in autophagy [64]. CLK1 encodes a member of the CDC2-like family of dual specificity protein kinases involved in pre-mRNA processing and may play an indirect role in governing splice site selection [65]. This may indicate a step-change in production of alternatively spliced gene products; however its role in disease pathogenesis is unclear. Interestingly a novel splice isoform, Pbx1-d, of the PBX1 gene (regulatory locus Sle1a.1-associated gene) has been implicated in the production of activated and auto-reactive CD4 positive T cells in a mouse model of lupus, through a defective response of CD4(+) T cells to retinoic acid-directed expansion of TGFβ-induced regulatory T cells [34,66]. This results in an overall reduction in peripheral T-regulatory cells. Pbx1-d over-expression is sufficient to induce an activated/inflammatory phenotype in Jurkat T cells and to decrease their apoptotic response to retinoic acid. The connection with retinoic acid-regulated responses is as yet unclear, however the role of retinoic acid in tuberculosis severity has been established in a rat model of disease [67]. EN2 (engrailed homeobox 2) has been recently identified as a regulator of T-cell differentiation [63] and ANPEP (aminopeptidase N or CD13) associated with phagocytosis regulation in myelomonocytic lineage cells: monocytes, macrophages, and dendritic cells [68]. EN2 is up-regulated until the four week time-point after which expression returns to pre-challenge levels. Expression of these may provide evidence of early initiation of monocyte activity and induction of a commensurate T-cell response.

Again, few statistically significant gene expression changes were observed between the pre-bleed and week two samples. Sixteen differentially regulated, statistically-significant entities were observed, FOS, KLF2, BEST3, IFIT2, B2M, ALS2CR11, IFI44, IFIT3, AC073517.6, GBP1, SOD2 (FAM177B), LGALS3BP, RP4-644F6.3(GBP1P1) and JAK2. All of these were up-regulated with respect to the previous week one time-point, which again also includes EN2. These provide the first evidence of onset of expression of interferon regulated entities. However, little direct evidence of expression of type I and II Interferons were observed at these time-points, apart from some low-level expression of IFNβ1 and IFNα21 in some of the animals of Chinese origin. Increasing expression of IFNGR1 was also seen in all animals from week one in the larger T4509 dataset, but no apparent IFNγ or IFNα1. It is inferred perhaps therefore that peripheral cell responses at these early time-points may occur in response to local tissue expression of Type I or II Interferons and other cytokines in response to events at a location distal to the periphery i.e. at the primary site of infection. IFIT2 and IFIT3 have been variably associated with onset or suppression of apoptosis [69].

FOS (c-FOS) is a leucine zipper protein that can dimerise with proteins of the JUN family, thereby forming the transcription factor complex AP-1. These have been implicated as regulators of cell proliferation, differentiation, and transformation, play a major role in cellular processes in a number of cell types and are a key immune regulator [70]. KLF2 is a Kruppel-like factor from a family of zinc finger transcription factors which regulates T-cell trafficking by promoting expression of the lipid-binding receptor S1P1 and selectin CD62L and may be involved in these initial T-cell associated processes [71]. However, it is also implicated in modulation of the activity of M2 (suppressor)-type macrophages [72] and development of B-cells [73]. It is not known as yet in which cell type expression of these factors is evident. LGALS3BP is also implicated in the immune response associated with natural killer and lymphokine-activated killer cell cytotoxicity [74] and may be involved in immunoregulation through interaction with CD33-like siglecs [75]. These may be an important factor in development of a suppressor-type cell response.

A larger number of statistically significant gene expression changes were observed between the pre-bleed and week four samples. Statistical analyses revealed 69 differentially regulated entities. These include IRF1, the scavenging receptor CD163 (M2 suppressor cell-associated marker [76]), E3 ubiquitin ligases SYVN1 and LNX2, the latter of which may be involved in CD8α-chain regulation, IL8 and IL12RB, among others. Again there is strong evidence of Interferon regulated entities as seen at week two and emergence of other entities which may be associated with anti-inflammatory immune functions i.e., CD163. The apparent up-regulation/abundance of CD163 would suggest substantial proliferation or infiltration of regulatory M2 cells of monocyte origin at this time-point, although there is little evidence of a similar degree of upregulation of the general myeloid marker CD33. This may be perhaps interpreted as upregulation of CD163 on existing peripheral myeloid cells, rather than an infiltration of newly recruited cells. Myeloid-derived suppressor cells have been detected in human tuberculosis and may be negatively correlated with CD4 and CD8 activation and function [77]. IL12RB expression was also now evident, although again there is no evidence of IL12 expression in these cells.

c-FOS, KLF2 and IL8 are also strongly down-regulated at this time-point concomitant with a significant fall in IL7R levels (p = 3.14 x 10^−7^). Expression profiles of c-FOS, IL7R and IL8 were confirmed by qPCR. IL8 down-regulation may be indicative of modulated neutrophil activity. IL7R is involved in T and B-cell development and survival and functions in T-cells by blocking apoptosis [78,79]. These observations would suggest that down-regulation of this receptor may play a key role in onset of apoptotic processes. This has been found to be down-regulated in active Tuberculosis in other studies and useful diagnostically as part of a multi-biomarker panel [43]. KLF2 regulates T-cell trafficking among other functions by promoting expression of the selectin CD62L and the lipid-binding receptor S1P1, whose expression is critical for T cell egress from the thymus, homing to the lymph nodes and then circulation in the periphery [80,81]. This may be associated with an early T-cell response. However, subsequent down-regulation of all these markers could reflect either transcriptional down-regulation of gene transcripts, or loss of cells from the periphery by other mechanisms i.e. egress, apoptosis or necrosis. Increased expression of c-FOS prior to loss of other markers and the observed stage specific expression of apoptotic markers may in part again suggest loss of cells through apoptotic processes, particularly in the CN group. Apoptosis, non-responsiveness or exhaustion of T-cells have been implicated in Tuberculosis disease progression, perhaps through the action of programmed cell death protein 1 [82–85]. However other mechanisms may also be involved as reported in this study. The cell–type specific expression associated with some of these markers e.g. c-FOS is unclear, except in cases of clear cell-type associated specificity e.g. CD163. These observations require further investigation to delineate the cell types associated with expression of these entities, through cell type-specific transcript mapping.

A very large number of statistically significant gene expression changes were observed between the pre-bleed and week six samples. Statistical analyses revealed 385 differentially regulated entities. Many of these entities have already exhibited substantial differential regulation at previous time-points, which remains largely unchanged e.g. GBP1 and RP4-644F6.3 (GBP1P1), CD163, PLAC8, SOD2 and CLIC1, which may be mononuclear/ macrophage-cell derived, VMP1 (TMEM49) and PLAC8 associated with autophagy/apoptosis. Other entities which exhibit a substantial difference in expression at this time-point are SAMD9L, FYB and SAG (up-regulation), NCR1 and MAPK6 and the major histocompatibility complex (MHC) class I-related gene RAET1G. These combined observations again provide evidence of a step-change in transcript expression/abundance between weeks four and six.

In a similar study, Kauschal [86] investigated mRNA expression in lung granulomas in a temporal Rhesus Macaque pulmonary TB study and found significant reprogramming of gene expression between unchallenged baseline controls and between the four and thirteen week time-points. This would support some of our observations of a significant immune reprogramming event around the four week time interval. In addition, these authors provided detailed temporal transcription information on key immune-associated entities, including IRF1, GBP1, IFNγ and many of the other markers identified in this study. Interestingly, only twenty-one of 136 immune gene entities highlighted as statistically significant and temporally expressed in their study were shared with our T4509 ANOVA dataset. These include CCL3, CCL18, CCRL2, SOCS7, IRF1, GBP1, IL7 and IFNγR1. They observed good temporal expression of IFNγ in NHP TB lung granulomas in addition to other cytokines and chemokines including IL1α, IL6 and IL7 among others. However expression of these entities appeared strongly down-regulated after the four week time-point. IFNγ expression was not observed in the peripheral cells in our study, at any time-point in any of the animals. IL12 a key cytokine in the protective response to TB [61,87] also did not appear to be expressed. This is not surprising as only faint signatures of IL12 are observed in TB and other infectious diseases [88]. In addition, although IFNγR1 was expressed in peripheral cells in our study, IFNγR2 expression was not apparent. This is interesting as both receptor chains appear to be expressed in granulomas in Kauschals study [86]. This would imply that either these peripheral cells are responding to a referred interferon signal produced at the site of infection with suppression of IFNγR2 expression. Or if these cells are re-circulating from a site of infection, that they are reprogrammed on egress, with concurrent down-regulation of some markers, chemokines and cytokines upon re-entry to the periphery e.g. IFNγR2. These observations warrant further investigation.

In another study by the same group, significant differences were observed between Rhesus Macaque derived bone macrophages (Rh-BMDMs) infected with hypoxia-adapted TB bacilli (Mtb-H) or organisms grown under conditions of normal oxygen tension (Mtb-A) [60]. Mtb-H were more susceptible to killing by Rh-BMDMs than Mtb-A and this was attributed primarily to expression of TNF (LTA) in Mtb-H infected Rh-BMDMs. This effect could be abrogated by transfection with anti-TNF siRNA in Mtb-A infected Rh-BMDMs. Other differences in gene expression were also observed in cross-comparative analyses between the Mtb-H and Mtb-A infected Rh-BMDMs, including interestingly FOS in the Mtb-H infected Rh-BMDMs, among others. A number of chemokines and receptors and cytokine induced genes were expressed in the Mtb-A infected compared with Mtb-H infected Rh-BMDMs, including GBP1 and other entities indicative of an IFNγ-induced signature e.g. IFI44, IFIT2 and IFIT3. This appears to be a common feature of these TB infection/immune cell-interaction studies. It may be inferred through this comparative analysis that pathogenic, non hypoxic-adapted TB bacilli may modify the host environment both *in vivo* and *in vitro* through similar mechanisms, i.e. modulation of expression of key immune regulators/factors e.g. FOS, TNF and interferon-regulated genes etc., presumably to gain a competitive survival advantage.

Other evidence which suggests emergence of an interferon-directed peripheral leukocyte response from week two onwards is provided by more detailed analysis of entities involved in type II interferon signalling and other interferon response factors (IRFs) which includes SPI1, GBP1, JAK2, IFNGR1 and IFI6. Sustained up-regulation of IRF1 and STAT1 was seen across all animals and time-points from week two onwards. Increased expression of CYBB was seen from week four onwards, particularly in the animals of CN origin. This corresponded with an increase in expression of IRF2 and OAS in this latter group. JAK1 expression was increased at Week 6 in both groups. STAT4 is progressively down-regulated in animals of MN origin, up-regulated to week 2 in the Chinese origin animals, then again down-regulated. This result was confirmed by qPCR and again perhaps indicates some repositioning of immune cellular responses from the two week time-point in the CN origin animals and from the week one time-point, post-infection in the animals of MN origin.

A number of markers were selected for further validation by qPCR to confirm their expression profile and to delineate markers suitable for ongoing study. Of the 347 validated features (342 select gene entities plus 5 gene and buffer controls) selected for further analysis, 237 exhibited a fold-change greater than 1.2 (68.3%), 223 a fold-change greater than 1.5 (64.27%) and 204 a fold-change greater than 2.0 (58.8%). Of these 72 were found to be statistically significant (p ≤ 0.05). No significant difference was discerned in the percent of entities successfully validated of those identified from microarray analyses or selected from other sources. This may reflect some inaccuracies on the part of the microarray hybridisation method used, perhaps due in part to the heterologous human/non-human primate array/mRNA hybridisation system. However, it does suggest that rigorous validation using an alternative method was required to confirm observations in this study.

qPCR validation analyses confirmed the differential regulation profiles of many entities including c-FOS, FAS, CD163, BIRC3 and the interferon regulated entities GBP1 and GBP6. Other entities found to be highly differentially regulated (FC ≥ 2.0) but not statistically significant included, IL8, IRF1, STAT1, PLAC8, CPVL, IFNGR1, SOCS3, SOD2 and ANPEP among others. c-FOS and IL7R were again found to be regulatory associated and both exhibit weak up-regulation to week 2, strong down-regulation at week 4, then some observed recovery at week 6 (given in Figures M & N S6 File). FAS and PLAC8 were incrementally up-regulated to week 6, and CD163 exhibited strong up-regulation at weeks 4 and 6 along with GBP1 and GBP6 and other interferon regulated entities (but again no apparent Type I or II interferons) from week 2 onwards. Some IFNβ1, IL10 and Il11 expression was observed in the CN animals at week four. These results again indicate a step change in immune regulation between weeks 2 and 4 and may suggest an apparent down-regulation of entities consistent with loss of key immune (perhaps T-cell) activities, with an increase in interferon regulated activities and an increase in entities associated with a more myeloid cell-driven response. This response is observed in animals from both lineages. There is little evidence of a T-cell response in the MN-derived animal’s pre- or post-challenge, however good evidence of T-cell activity (mainly CD8) is observed in the CN animals.

The results presented in this study showed evidence of a polarised immune response between the two NHP lineages, with a strong adaptive (albeit perhaps ultimately abortive) immune response evident in the CN lineage animals, with over-representation of T cell–derived markers e.g. CD2, CD8α and CD8β, CD4 and IL2R etc. to at least the 2 week time-point and an apparent lack of expression these markers in MN-derived animals at any time-point. The CN animals appeared to down-regulate T-cell markers at four weeks including CD4, CD3ε and CD3ς. While CD4 expression may be partially restored at the 6 week time-point, restoration of CD3ε and CD3ς expression was not evident. Down-regulation of CD3 T-cell receptor sub-components has been observed previously around the site of granulomas [89], although commensurate down-regulation of CD3ε was not observed. This may be co-incident with increasing expression of GBP1 which, in addition to other antimicrobial functions [90], may also function as a T-cell receptor regulator [91]. These animals do also show some evidence of IL10 and IL11 expression at the four week time-point by qPCR. In contrast, there is strong constitutive expression and thereafter a further increase of the myeloid marker CD33 in the MN-derived animals, commensurate with increasing up-regulation of cell associated inflammatory markers such as IL1R1 and IL18R. They appear to exhibit evidence of a defective T-cell response and this may be related to the innate predominance of a CD33 (Siglec-3)-expressing myeloid-type immune cell. As stated previously, CD33 has been associated previously with acute myeloid leukaemia in humans [92]. The siglecs are a family of anti-inflammatory immune-regulatory sialic acid-binding immunoglobulin-like lectins [93,94], perhaps through direct association with TLRs [95]. Other highly differentially regulated but not statistically significant markers in the MN animals include TLR2, CLEC4E (MINCLE), the anti-apoptotic decoy marker TNFRSF10D (CD8/NKT lymphocyte-specific receptor TRAIL-R4) and pro-apoptotic markers APAF1 and BAX, particularly at week 1. This is coincident with a transient expression of other markers e.g. TLR3 and TLR7. These are not seen in the animals of CN lineage. There appears to be a complete absence of expression of CD8β, MIF and NFRKB in the MN-derived animals and no expression of IL18R or IL1R1 in the CN lineage animals. These former animals exhibited greater innate sensitivity to infection with Tubercle bacilli than the CN animals and this may be reflected in these apparent differences in their immune response.

ANN analysis of the datasets revealed some interesting additional information with regard to key significant biomarkers, but also the regulatory networks at play in the ongoing response to TB challenge, not revealed using parametric analysis tools. These results revealed some interesting alternative biomarkers, not identified previously using the parametric analyses. Of particular interest is IL15. While not significant in the T4509 entity list, this cytokine was identified using these alternate methods. This is of particular interest due to the fact that IL15 and IL2 act synergistically to regulate NK and CD8 T-cell proliferation and activation [96]. There is little evidence of peripheral IL2 expression; however IL15 expression would again suggest involvement of NK or CD8 cells during the early response. The NHP groups of different origins exhibited different regulatory profiles with regard to programmed cell death markers, with the CN animals expressing a more pro-apoptotic profile. The MN animals exhibited a profile consistent with suppression of apoptosis through BCL2A1 and BCL2L2. This may play an important part in innate susceptibility, as apoptotic cell death of TB infected cells is considered important in eradication of the pathogen [97].

In addition to investigating the primary response to Tuberculosis in this primate model our aim was to utilise this information to identify biomarkers which may be of improved utility in diagnosing Tuberculosis in humans. Parametric and non-parametric (ANN) ranked data outputs were cross-compared and revealed 222 markers which exhibited greater consistency of expression across time-points in the primate infection data. A large number of up-regulated markers and a smaller number of down-regulated markers were identified. To further delineate markers which may be expressed in both NHPs and humans, we compared this refined dataset to a previously published human datasets [34, 35] using both the multiomic pathway and Venn diagram analysis functions of GX12.5. These revealed only thirty markers which are highly significant across all three data lists. These include a number of markers associated with immune function, including some previously highlighted in this study i.e. GBP1, JAK2, IRF1 and STAT1 and important entities in the type II interferon pathway e.g. FYB. The expression profiles of four of these could be confirmed using qPCR analysis, GBP1, IRF1, STAT1 and PLAC8.

All NHP and human entities as outlined in Table 2 may be useful for diagnosis of active TB in primates including humans and may show enhanced utility across disparate ethnic groups. GBP1 is highly up-regulated in active TB and down-regulated in latent TB and may be of particular significance as it has been recently identified as an IFNγ-regulated negative regulator of T-cell activation [91]. It may also be involved in the targeting of infected phagosomes for lysosomal degradation through mechanisms of autophagy in antigen presenting cells [98] and play a role in cytokine mediated anti-proliferative mechanisms in other cell types [99]. This is a highly statistically significant entity in all NHP and human datasets published and is found to be increasingly highly expressed even in individuals with HIV/TB co-infection (from reference [39]).

Expression of GBP1 and PSMB9 may be associated with expression of IRF2, which competitively inhibits the IRF1-mediated transcriptional activation of interferons alpha and beta and functions as a transcriptional activator of histone H4. This was found to be up-regulated from the four week time-point, particularly in the animals of MN lineage. There is evidence from experiments of sorted peripheral blood leukocytes of moderate up-regulation of GBP1, IRF1 and PSMB9 in CD4 and CD8 T-cells, but significantly higher expression of these entities in monocytes and neutrophils in human active TB (from [34]). IRF2 up-regulation appears confined to monocytes and neutrophils in this study. Further study is required to precisely delineate cell-specific expression of these markers, particularly in the CD163-expressing M2 myeloid cells, which may play an important role in immune regulation, through suppression of T-cell responses [100,101]. Expansion of these cell types may be driven by IFNγ and is regulated through JAK/STAT3 activation.

In summary, we have shown that non-human primates exhibit a substantial peripheral blood leukocyte response subsequent to pulmonary challenge with aerosolised Tubercle bacilli. Large numbers of gene entities exhibited shifting temporal expression patterns across weekly time-points, as the infection proceeded. There appeared to be a significant step-change in gene entity expression profiles between the 2 and 4 week time-points and a more substantial transcript differential expression change at the week 6 time point, which may show some evidence of a drive toward to a more Type II interferon-driven response. This may be additionally associated with an increase in activity of a myeloid suppressor cell phenotype. There is some evidence that in severe TB, excessive IFNγ production is not protective and may in fact lead to anergy [61]. This may be due to elevation of other IFN-regulated down-stream factors e.g. GBP1, which appear to have regulatory activity. Other regulators e.g. IL10, IL11 and IL15 may also contribute to T-cell adaptive immune response suppression.

A number of entities derived from microarray analysis and from other sources were investigated for validation purposes using qPCR. This revealed near equivalent numbers of significantly dysregulated features per group, indicating the necessity of follow-on validation of gene entities from microarray studies. The expression profiles of a number of our most significant gene entities were confirmed using this method. In order to select entities for ongoing analysis and diagnostic test development, ANN and parametric selection of the data outputs from this study and cross-comparison to two human data sets was conducted. This revealed a highly select numbers of markers which are currently in further development for diagnostic purposes.

## Supporting Information

S1 File Tables (A) to (J); statistically significant entity lists from all analyses outlined in above sections. (XLSX)

S2 File Tables (A) to (I); statistically significant pathways specific to individual clusters from analyses depicted graphically in Fig 1. (PDF)

S3 File Figures (A) to (E); network inference maps of Entities from the T100ANN and T50 VS Datasets from combined and MN or CN specific data analyses. (PDF)

S4 File Figures (A) and (B) and Table (A); entities shared between NHP-TB top 1000 ranked entities from artificial neural network analysis and top 1000 ranked entities from Analysis of Variance. (PDF)

S5 File Figures (A) and (B); Heat maps of Expression Patterns of the 30 Preferred Biomarker entities in Previously Published Human Datasets GEO IDs GSE19439 and GSE28623. (PDF)

S6 File Figures (A) to (V); Line graph depiction of qPCR analyse outputs of key differentially expressed entities from both CN and MN datasets. (PDF)
