# Correction: Temporal Expression of Peripheral Blood Leukocyte Biomarkers in a *Macaca fascicularis* Infection Model of Tuberculosis; Comparison with Human Datasets and Analysis with Parametric/Non-parametric Tools for Improved Diagnostic Biomarker Identification

**DOI:** 10.1371/journal.pone.0159783

**Published:** 2016-07-18

**Authors:** Sajid Javed, Leanne Marsay, Alice Wareham, Kuiama S. Lewandowski, Ann Williams, Michael J. Dennis, Sally Sharpe, Richard Vipond, Nigel Silman, Graham Ball, Karen E. Kempsell

The text in Fig 1 highlighting rows of expression data by week is incorrectly reversed. The authors have provided a corrected version here.

**Fig 1:**
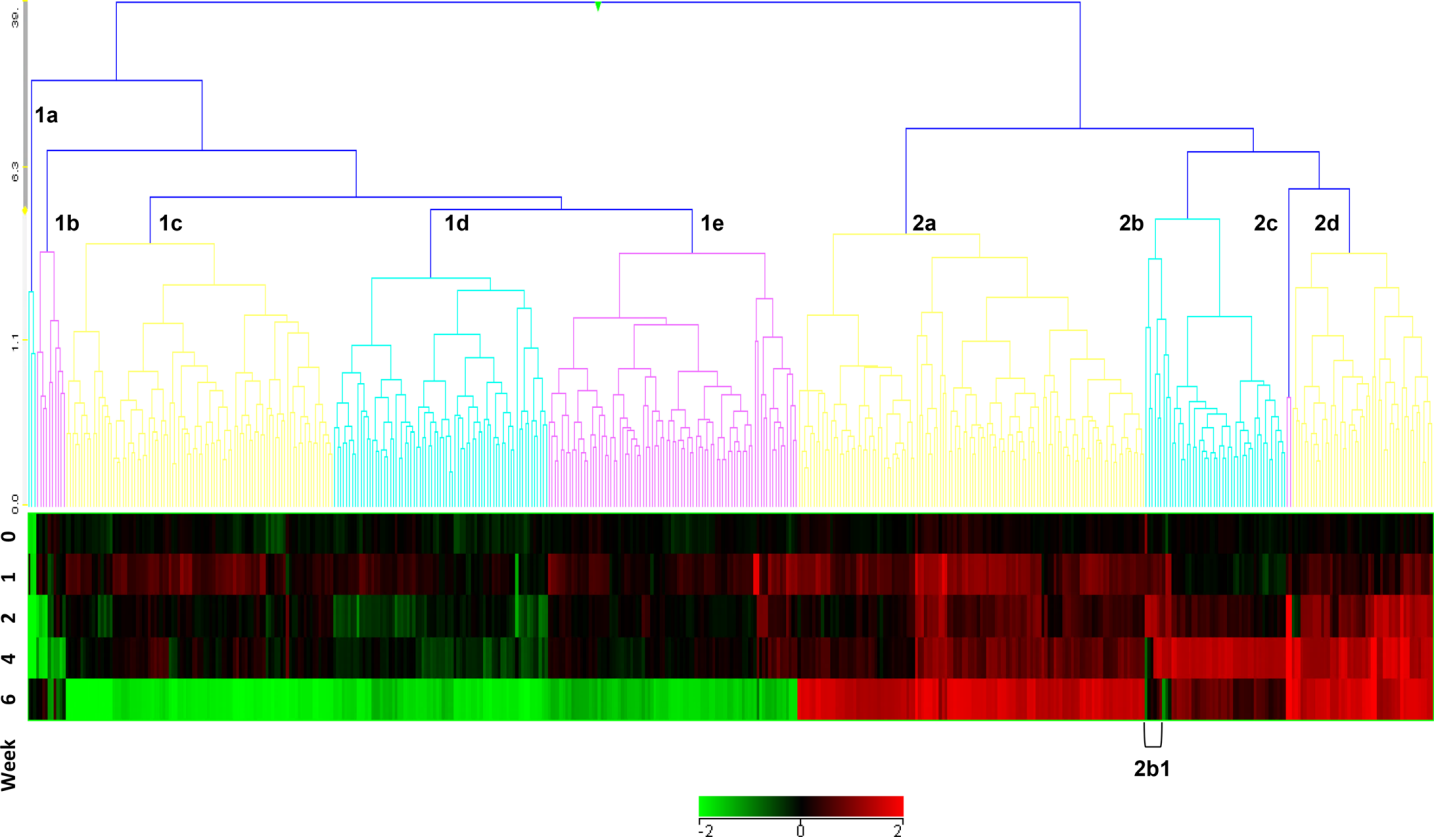
Cluster analysis of temporally expressed entities in peripheral blood leukocytes of Cynomolgus Macaques (all animals) pre-(week 0) or post (weeks 1–6) aerosol-challenge with *M*. *tuberculosis*. These exhibit patterns of up- (cluster 2) or down-regulation (cluster 1) across the six week time course of the experiment. Cluster 2b1 (highlighted) contains co-expressed entities, FOS, IL8 and KLF2.
