# Supplementary material for: Temporal Expression of Peripheral Blood Leukocyte Biomarkers in a Macaca fascicularis Infection Model of Tuberculosis; Comparison with Human Datasets and Analysis with Parametric/Non-parametric Tools for Improved Diagnostic Biomarker Identification
Source: PLoS One. 2016 May 26;11(5):e0154320. doi: 10.1371/journal.pone.0154320 (PMC4882019; doi:10.1371/journal.pone.0154320)
Supplement: S2 File — (PDF) [file pone.0154320.s002.pdf]

Supplementary Information S2

Table A

T478 all clusters – Statistically Significant Pathways p < 0.05	p-value	Matched Entities	Pathway Entities of Experiment Type
Hs_Type_II_interferon_signaling_(IFNG)_WP619_67733	0.000619	4	37
Hs_Type_II_interferon_signaling_(IFNG)_WP619_45027	0.000619	4	37
Hs_Eukaryotic_Translation_Elongation_WP1811_45249	0.001475	2	5
Hs_Oxidative_Stress_WP408_59210	0.004349	3	30
Hs_Oxidative_Stress_WP408_45296	0.004349	3	30
Hs_Vitamin_D_synthesis_WP1531_46487	0.006374	2	11
Hs_Interferon_gamma_signaling_WP1836_44865	0.007728	2	13
Hs_Prolactin_Signaling_Pathway_WP2037_46205	0.01043	4	75
Hs_Prolactin_Signaling_Pathway_WP2037_67606	0.010957	4	76
Hs_IL-6_signaling_pathway_WP364_44848	0.012193	3	42
Hs_IL-3_Signaling_Pathway_WP286_44845	0.014909	3	45
Hs_miRs_in_Muscle_Cell_Differentiation_WP2012_59102	0.018202	2	40
Hs_TCR_Signaling_Pathway_WP69_48452	0.022638	4	91
Hs_TCR_Signaling_Pathway_WP69_45093	0.022638	4	91
Hs_Kit_receptor_signaling_pathway_WP304_67427	0.023695	3	57
Hs_Kit_receptor_signaling_pathway_WP304_44883	0.023695	3	57
Hs_Bile_acid_and_bile_salt_metabolism_WP1788_44976	0.027214	2	24
Hs_Physiological_and_Pathological_Hypertrophy_of_the_Heart_WP1528_45248	0.029696	2	24
Hs_GPCRs,_Class_B_Secretin-like_WP334_45339	0.029696	2	23
Hs_TSH_signaling_pathway_WP2032_67727	0.033233	3	65
Hs_TSH_signaling_pathway_WP2032_44635	0.033233	3	65
Hs_EPO_Receptor_Signaling_WP581_41162	0.034916	2	26
Hs_Gap_junction_trafficking_and_regulation_WP1820_42043	0.036491	1	4
Hs_Folate_Metabolism_WP176_68034	0.037845	3	68
Hs_Folate_Metabolism_WP176_45271	0.037845	3	68
Hs_miRs_in_Muscle_Cell_Differentiation_WP2012_45377	0.040461	2	40
Hs_Factors_involved_in_megakaryocyte_development_and_platelet_production_WP1815_42038	0.040461	2	28
Hs_SIDS_Susceptibility_Pathways_WP706_45233	0.041091	3	66
Hs_Serotonin_Receptor_2_and_STAT3_Signaling_WP733_62393	0.048357	1	4
Hs_Serotonin_Receptor_2_and_STAT3_Signaling_WP733_45035	0.048357	1	4

Table B

T478 Cluster 1a entities – Statistically Significant Pathways $p < 0.05$	p-value	Matched Entities	Pathway Entities of Experiment Type
Hs_Glutathione_metabolism_WP100_45319	0.001693	1	20
Hs_Squamous_cell_TarBase_WP2006_44276	0.011074	1	124
Hs_Leukocyte_TarBase_WP2003_44886	0.011809	1	128
Hs_Muscle_cell_TarBase_WP2005_44926	3.19E-02	1	336
Hs_Lymphocyte_TarBase_WP2004_46264	0.039071	1	420

Table C

T478 Cluster 1b entities – Statistically Significant Pathways $p < 0.05$	p-value	Matched Entities	Pathway Entities of Experiment Type
None detected	NA	NA	NA

Table D

T478 Cluster 1c entities – Statistically Significant Pathways $p < 0.05$	p-value	Matched Entities	Pathway Entities of Experiment Type
Hs_SIDS_Susceptibility_Pathways_WP706_45233	0.006721	2	66
Hs_Synthesis_and_Degradation_of_Ketone_Bodies_WP311_67674	0.009841	1	5
Hs_Synthesis_and_Degradation_of_Ketone_Bodies_WP311_67674	0.009841	1	5
Hs_Synthesis_and_Degradation_of_Ketone_Bodies_WP311_45110	0.009841	1	5
Hs_SIDS_Susceptibility_Pathways_WP706_68050	0.010719	2	166
Hs_SIDS_Susceptibility_Pathways_WP706_68050	0.010719	2	166
Hs_Neurotransmitter_Release_Cycle_WP1871_42089	0.021526	1	15
Hs_Neurotransmitter_Release_Cycle_WP1871_42089	0.021526	1	15
Hs_Serotonin_Transporter_Activity_WP1455_46053	0.021526	1	11

Hs_Serotonin_Transporter_Activity_WP1455_46053	0.021526	1	11
Hs_Costimulation_by_the_CD28_family_WP1799_42022	0.02346	1	15
Hs_Interleukin-1_signaling_WP1839_44873	0.027317	1	15
Hs_Interleukin-1_signaling_WP1839_44873	0.027317	1	15
Hs_Biogenic_Amine_Synthesis_WP550_44978	0.02924	1	15
Hs_SREBF_and_miR33_in_cholesterol_and_lipid_homeostasis_WP2011_44729	0.031159	1	18
Hs_miRs_in_Muscle_Cell_Differentiation_WP2012_59102	0.033074	1	40
Hs_miRs_in_Muscle_Cell_Differentiation_WP2012_59102	0.033074	1	40
Hs_Insulin_Signaling_WP481_45380	0.034285	2	161
Hs_MAPK_signaling_pathway_WP382_44890	0.035571	2	161
Hs_MAPK_signaling_pathway_WP382_67443	0.036003	2	162
Hs_MAPK_signaling_pathway_WP382_67443	0.036003	2	162
Hs_Physiological_and_Pathological_Hypertrophy_of_the_Heart_WP1528_45248	0.042596	1	24
Hs_Physiological_and_Pathological_Hypertrophy_of_the_Heart_WP1528_45248	0.042596	1	24
Hs_GPCRs,_Class_B_Secretin-like_WP334_45339	0.0426	1	23
Hs_NGF_signalling_via_TRKA_from_the_plasma_membrane_WP1873_42091	0.048265	1	31
Hs_NGF_signalling_via_TRKA_from_the_plasma_membrane_WP1873_42091	0.0483	1	31

Table E

T478 Cluster 1d entities – Statistically Significant Pathways $p < 0.05$	p-value	Matched Entities	Pathway Entities of Experiment Type
Hs_Gap_junction_trafficking_and_regulation_WP1820_42043	0.005494	1	4
Hs_Eukaryotic_Translation_Elongation_WP1811_45249	0.009141	1	5
Hs_GPCRs,_Class_B_Secretin-like_WP334_45339	0.039612	1	23
Hs_ACE_Inhibitor_Pathway_WP554_44938	0.028964	1	17

Table F

T478 Cluster 1e entities – Statistically Significant Pathways $p < 0.05$	p-value	Matched Entities	Pathway Entities of Experiment Type
Hs_Integrated_Cancer_pathway_WP1971_44858	0.001427828	2	36
Hs_DNA_damage_response_WP707_45196	0.005087666	2	68
Hs_miRNA_regulation_of_DNA_Damage_Response_WP1530_45379	0.005603608	2	98
Hs_Peptide_GPCRs_WP24_45244	0.006327245	2	73
Hs_Peptide_GPCRs_WP24_45244	0.006327245	2	73
Hs_Fanconi_Anemia_pathway_WP1816_42159	0.008965871	1	6
Hs_Fanconi_Anemia_pathway_WP1816_42159	0.008965871	1	6
Hs_Androgen_receptor_signaling_pathway_WP138_44951	0.009618565	2	85
Hs_Eukaryotic_Translation_Initiation_WP1812_45250	0.010749565	1	9
Hs_Eukaryotic_Translation_Initiation_WP1812_45250	0.010749565	1	9
Hs_Regulation_of_Apoptosis_WP1896_45054	0.012530111	1	8
Hs_Metabolism_of_nucleotides_WP1851_44898	0.016081782	1	12
Hs_Metabolism_of_nucleotides_WP1851_44898	0.016081782	1	12
Hs_Steroid_Biosynthesis_WP496_59804	0.016081782	1	11
Hs_Steroid_Biosynthesis_WP496_59804	0.016081782	1	11
Hs_Steroid_Biosynthesis_WP496_45151	0.01785292	1	11
Hs_Interferon_gamma_signaling_WP1836_44865	0.019620929	1	13
Hs_Double-Strand_Break_Repair_WP1807_45201	0.0231	1	18
Hs_Double-Strand_Break_Repair_WP1807_45201	0.023147594	1	18
Hs_TP53_network_WP1742_59109	0.024906259	1	22
Hs_TP53_network_WP1742_59109	0.024906259	1	22
Hs_Integrated_Breast_Cancer_Pathway_WP1984_46412	0.029956723	2	164
Hs_Apoptosis_Modulation_by_HSP70_WP384_41236	0.030163651	1	18
Hs_TP53_network_WP1742_45074	0.03365313	1	22
Hs_Intrinsic_Pathway_for_Apoptosis_WP1841_44875	0.03539325	1	21
Hs_Bile_acid_and_bile_salt_metabolism_WP1788_44976	0.0371303	1	24
Hs_Bile_acid_and_bile_salt_metabolism_WP1788_44976	0.0371303	1	24
Hs_One_Carbon_Metabolism_WP241_45325	0.045769643	1	27

Table G

T478 Cluster 2a entities – Statistically Significant Pathways p < 0.05	p-value	Matched Entities	Pathway Entities of Experiment Type
Hs_Vitamin_D_synthesis_WP1531_46487	0.00502	2	11
Hs_Ganglio_Sphingolipid_Metabolism_WP1423_45306	0.020154	1	10
Hs_Adipocyte_TarBase_WP2001_44940	0.049634	1	17

Table H

T478 Cluster 2b entities– Statistically Significant Pathways p < 0.05	p-value	Matched Entities	Pathway Entities of Experiment Type
Hs_IL-3_Signaling_Pathway_WP286_44845	0.001534382	2	45
Hs_Endochondral_Ossification_WP474_45241	0.002711297	2	64
Hs_Eukaryotic_Translation_Elongation_WP1811_45249	0.0068	1	5
Hs_Toll-like_receptor_signaling_pathway_WP75_46210	0.007143688	2	102
Hs_Regulation_of_toll-like_receptor_signaling_pathway_WP1449_59207	0.007143688	2	150
Hs_MAPK_targets-_Nuclear_events_mediated_by_MAP_kinases_WP1845_44891	0.010957256	1	8
Hs_Regulation_of_toll-like_receptor_signaling_pathway_WP1449_45049	0.013248001	2	150
Hs_Post-translational_modification-_gamma_carboxylation_and_hypusine_formation_WP1886_42102	0.0137	1	11
Hs_Estrogen_signaling_pathway_WP712_45247	0.024489416	1	20
Hs_EBV_LMP1_signaling_WP262_41154	0.028513765	1	23
Hs_Physiological_and_Pathological_Hypertrophy_of_the_Heart_WP1528_45248	0.029851617	1	24
Hs_Matrix_Metalloproteinases_WP129_44892	0.032521937	1	31
Hs_Matrix_Metalloproteinases_WP129_67451	0.032521937	1	31
Hs_Oxidative_Stress_WP408_59210	0.036513995	1	30
Hs_Oxidative_Stress_WP408_45296	0.036513995	1	30
Hs_Serotonin_HTR1_Group_and_FOS_Pathway_WP722_49735	0.04049	1	34
Hs_Signaling_of_Hepatocyte_Growth_Factor_Receptor_WP313_45129	0.04049	1	34
Hs_Serotonin_HTR1_Group_and_FOS_Pathway_WP722_46093	0.04049	1	34
Hs_TSLP_Signaling_Pathway_WP2203_67728	0.04576648	1	38

Hs_Translation_Factors_WP107_45069	0.04708118	1	50
Hs_IL-5_signaling_pathway_WP127_45368	0.049705278	1	40

Table I

T478 Cluster 2c/2d entities – Statistically Significant Pathways p < 0.05	p-value	Matched Entities	Pathway Entities of Experiment Type
Hs_Type_II_interferon_signaling_(IFNG)_WP619_67733	2.11E-07	4	37
Hs_Factors_involved_in_megakaryocyte_development_and_platelet_production_WP1815_42038	8.17E-04	2	28
Hs_IL-6_signaling_pathway_WP364_44848	0.001838075	2	42
Hs_TSH_signaling_pathway_WP2032_67727	0.003886158	2	65
Hs_TSH_signaling_pathway_WP2032_44635	0.003886158	2	65
Hs_Selenium_Pathway_WP15_68043	0.005642252	2	85
Hs_Prolactin_Signaling_Pathway_WP2037_46205	0.005642252	2	75
Hs_Selenium_Pathway_WP15_68043	0.005642252	2	85
Hs_Selenium_Pathway_WP15_45021	0.005642252	2	85
Hs_Prolactin_Signaling_Pathway_WP2037_67606	0.005802231	2	76
Hs_Serotonin_Receptor_2_and_STAT3_Signaling_WP733_45035	0.006476914	1	4
Hs_Serotonin_Receptor_2_and_STAT3_Signaling_WP733_62393	0.006476914	1	4
Hs_Senescence_and_Autophagy_WP615_45024	0.009226647	2	98
Hs_Signal_regulatory_protein_(SIRP)_family_interactions_WP1909_45225	0.011307649	1	9
Hs_Interferon_gamma_signaling_WP1836_44865	0.017712902	1	13
Hs_RNA_Polymerase_II_Transcription_WP1906_45042	0.024077555	1	19
Hs_Nucleotide_Metabolism_WP404_45328	0.025662404	1	19
Hs_Type_II_diabetes_mellitus_WP1584_45036	0.027244736	1	21
Hs_Eicosanoid_Synthesis_WP167_45234	0.027244736	1	25
Hs_Interleukin-3,_5_and_GM-CSF_signaling_WP1840_44874	0.028824553	1	19
Hs_Nicotine_Activity_on_Dopaminergic_Neurons_WP1602_45361	0.031976663	1	21
Hs_Nicotine_Activity_on_Dopaminergic_Neurons_WP1602_47517	0.031976663	1	21
Hs_Triacylglyceride_Synthesis_WP325_45059	0.03354896	1	24
Hs_Triacylglyceride_Synthesis_WP325_48243	0.03354896	1	24
Hs_TCR_signaling_WP1927_45094	0.03354896	1	21
Hs_EPO_Receptor_Signaling_WP581_41162	0.038250882	1	26

Hs_Metabolism_of_amino_acids_and_derivatives_WP1847_52373	0.041373055	1	31
Hs_Metabolism_of_amino_acids_and_derivatives_WP1847_44894	0.041373055	1	31
Hs_Oxidative_Stress_WP408_59210	0.04293042	1	30
Hs_Oxidative_Stress_WP408_45296	0.04293042	1	30
Hs_Fatty_acid,_triacylglycerol,_and_ketone_body_metabolism_WP1817_42160	0.044485316	1	28
Hs_Fatty_Acid_Beta_Oxidation_WP143_59762	0.047587685	1	34
Hs_Fatty_Acid_Beta_Oxidation_WP143_45258	0.047587685	1	34
