# Supplementary material for: Temporal Expression of Peripheral Blood Leukocyte Biomarkers in a Macaca fascicularis Infection Model of Tuberculosis; Comparison with Human Datasets and Analysis with Parametric/Non-parametric Tools for Improved Diagnostic Biomarker Identification
Source: PLoS One. 2016 May 26;11(5):e0154320. doi: 10.1371/journal.pone.0154320 (PMC4882019; doi:10.1371/journal.pone.0154320)
Supplement: S3 File — (PDF) [file pone.0154320.s003.pdf]

Supplementary Information S3; Network inference maps of Entities from the T100ANN and T50 VS Datasets

(A) T100ANN all entities all animals (B) T100ANN CN animals (C) T100ANN MN animals (D) T50 VS all entities CN animals (E) T50 VS all entities MN animals

(A)

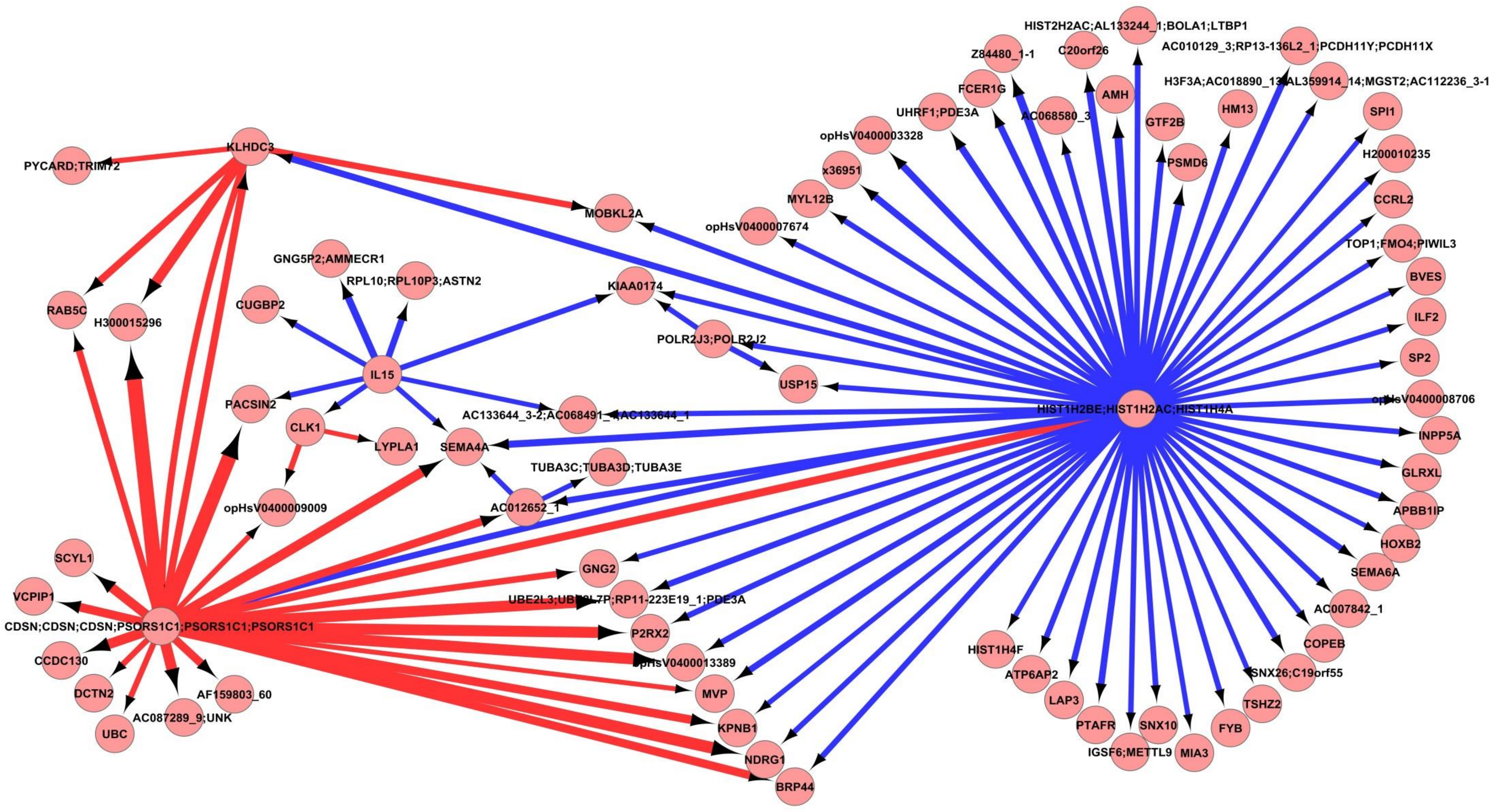

(B)

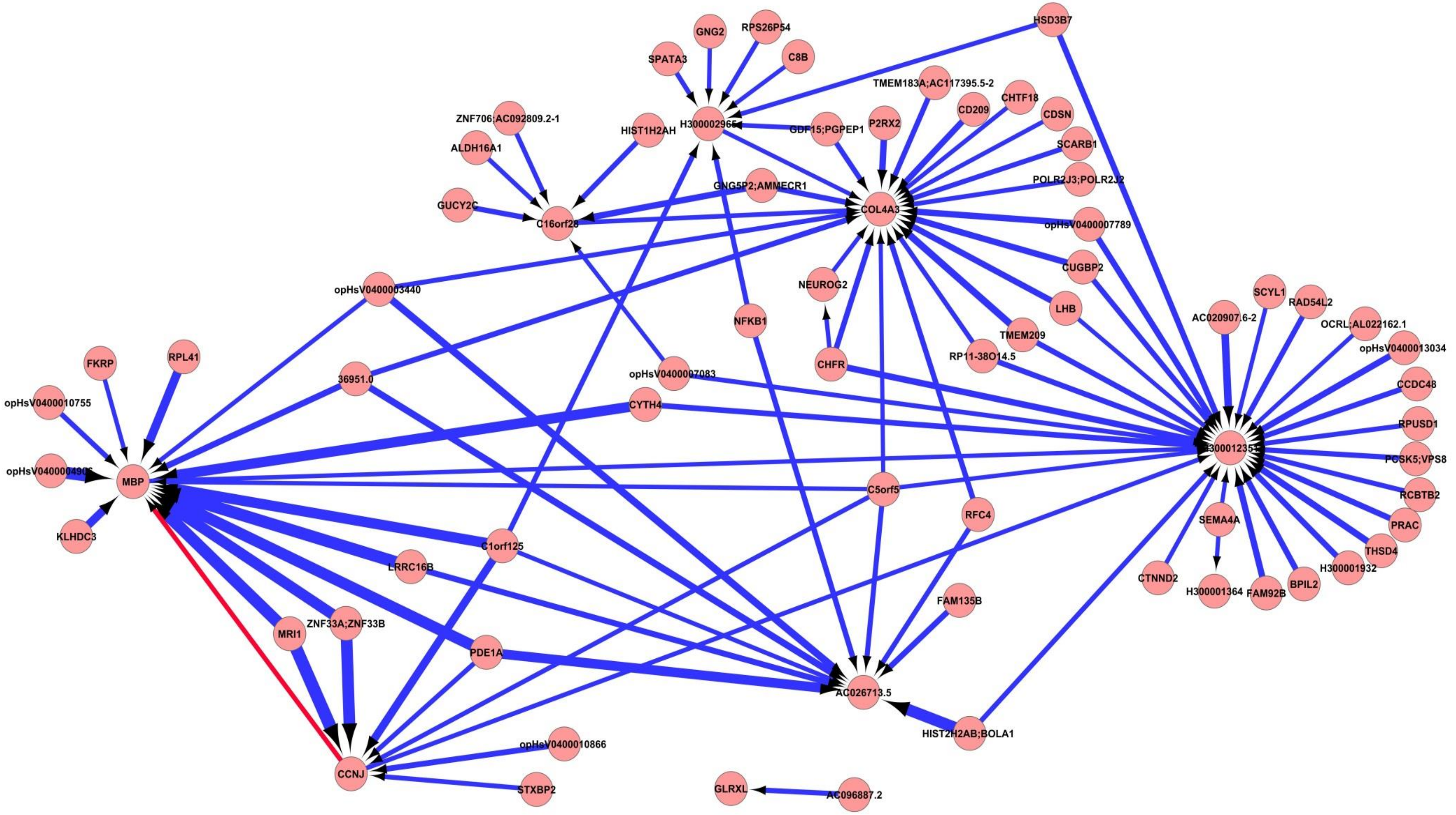
HSD3B7
GNG2
RPS26P54
SPATA3
C8B
TMEM183A;AC117395.5-2
CD209
CHTF18
CDSN
ZNF706;AC092809.2-1
HIST1H2AH
H300002965
GDF15;PGPEP1
P2RX2
SCARB1
ALDH16A1
POLR2J3;POLR2J2
GNG5P2;AMMECR1
GUCY2C
C16orf28
COL4A3
opHsV0400007789
CUGBP2
opHsV0400003440
NEUROG2
NFKB1
LHB
SCYL1
RAD54L2
AC020907.6-2
OCRL;AL022162.1
TMEM209
opHsV0400013034
RP11-38O14.5
CHFR
FKRP
RPL41
opHsV0400007083
36951.0
CCDC48
opHsV0400010755
CYTH4
RPUSD1
PCSK5;VPS8
MBP
C5orf5
RCBTB2
RFC4
SEMA4A
PRAC
KLHDC3
C1orf125
THSD4
LRRC16B
CTNND2
H300001932
H300001364
FAM92B
BPIL2
FAM135B
MRI1
ZNF33A;ZNF33B
PDE1A
AC026713.5
HIST2H2AB;BOLA1
opHsV0400010866
CCNJ
STXBP2
GLRXL
AC096887.2

(C)

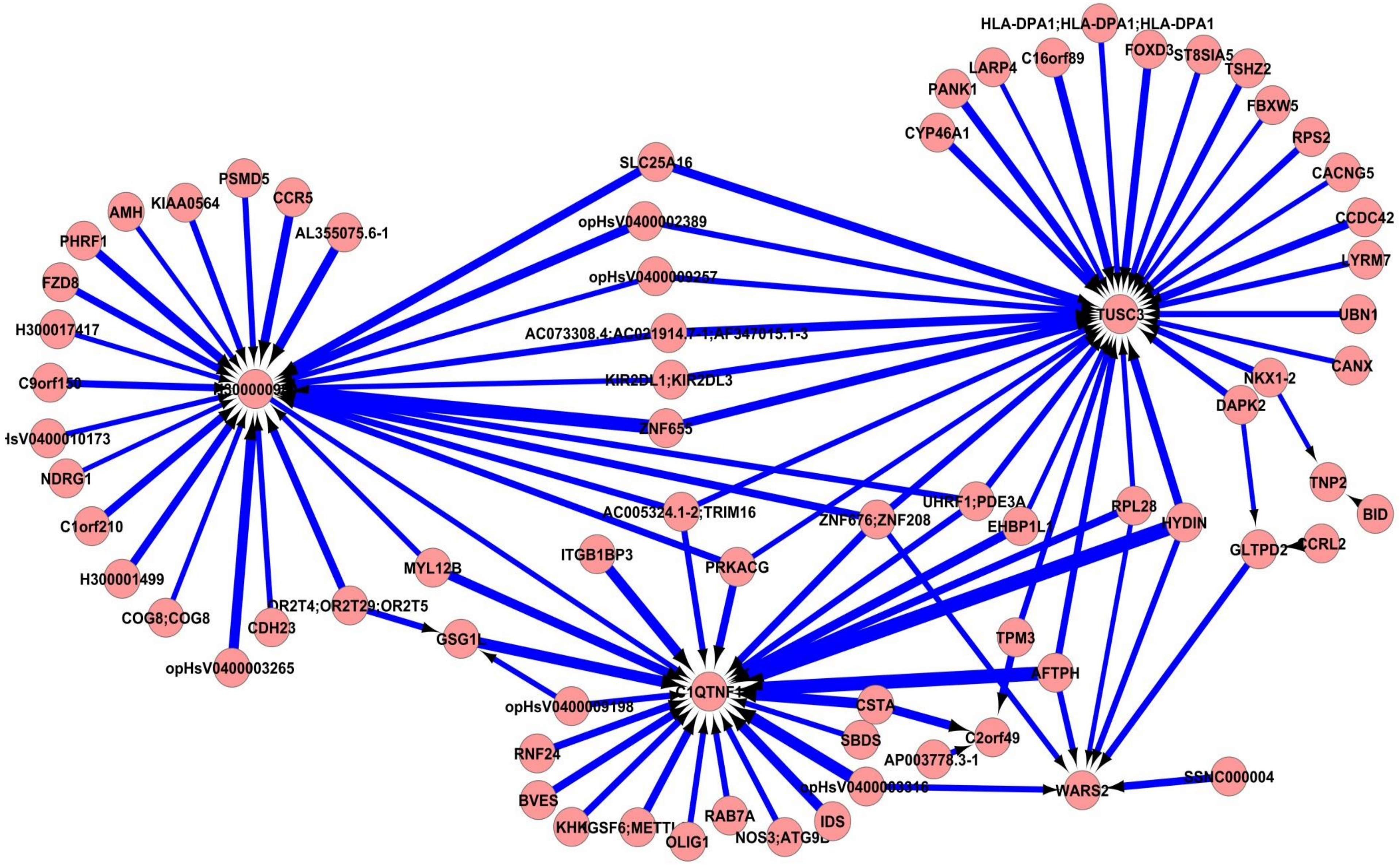

HLA-DPA1;HLA-DPA1;HLA-DPA1
FOXD3
ST8SIA5
C16orf89
LARP4
TSHZ2
PANK1
FBXW5
CYP46A1
RPS2
CACNG5
CCDC42
LYRM7
UBN1
TUSC3
CANX
NKX1-2
DAPK2
TNP2
BID
GLTPD2
CCRL2
SLC25A16
opHsV0400002389
KIR2DL1;KIR2DL3
ZNF655
PSMD5
CCR5
AMH
KIAA0564
AL355075.6-1
PHRF1
FZD8
H300017417
NDRG1
C1orf210
H300001499
COG8;COG8
CDH23
OR2T4;OR2T29;OR2T5
opHsV0400003265
MYL12B
ITGB1BP3
PRKACG
AC005324.1-2;TRIM16
ZNF676;ZNF208
UHRF1;PDE3A
EHBP1L1
RPL28
HYDIN
TPM3
AFTPH
GSG1L
CSTA
C2orf49
SBDS
AP003778.3-1
WARS2
SSNC000004
RNF24
BVES
OLIG1
RAB7A
IDS

(D)

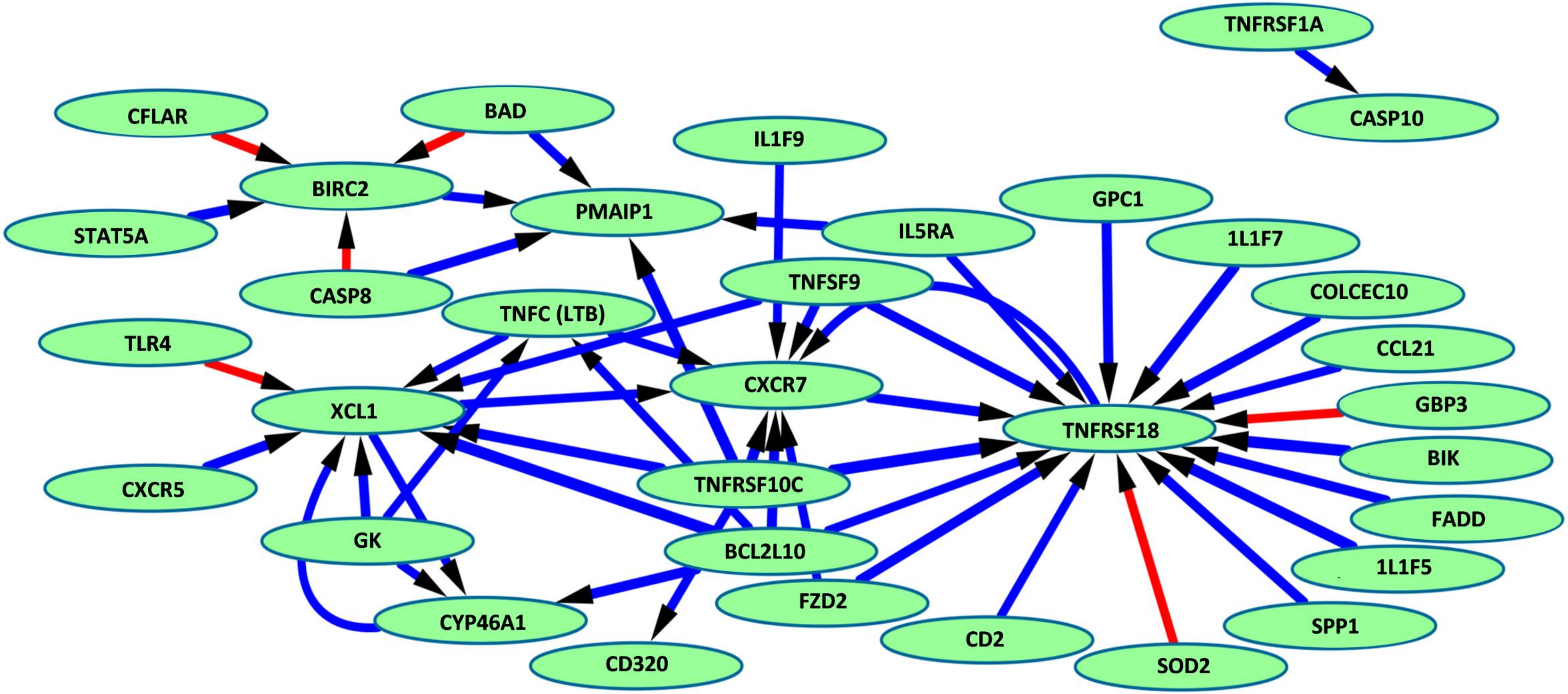
TNFRSF1A
CASP10
CFLAR
BAD
BIRC2
IL1F9
STAT5A
PMAIP1
GPC1
IL5RA
1L1F7
CASP8
TNFSF9
COLCEC10
TNFC (LTB)
TLR4
CCL21
CXCR7
GBP3
XCL1
TNFRSF18
BIK
TNFRSF10C
CXCR5
FADD
GK
BCL2L10
1L1F5
FZD2
CYP46A1
SPP1
CD2
SOD2
CD320

(E)

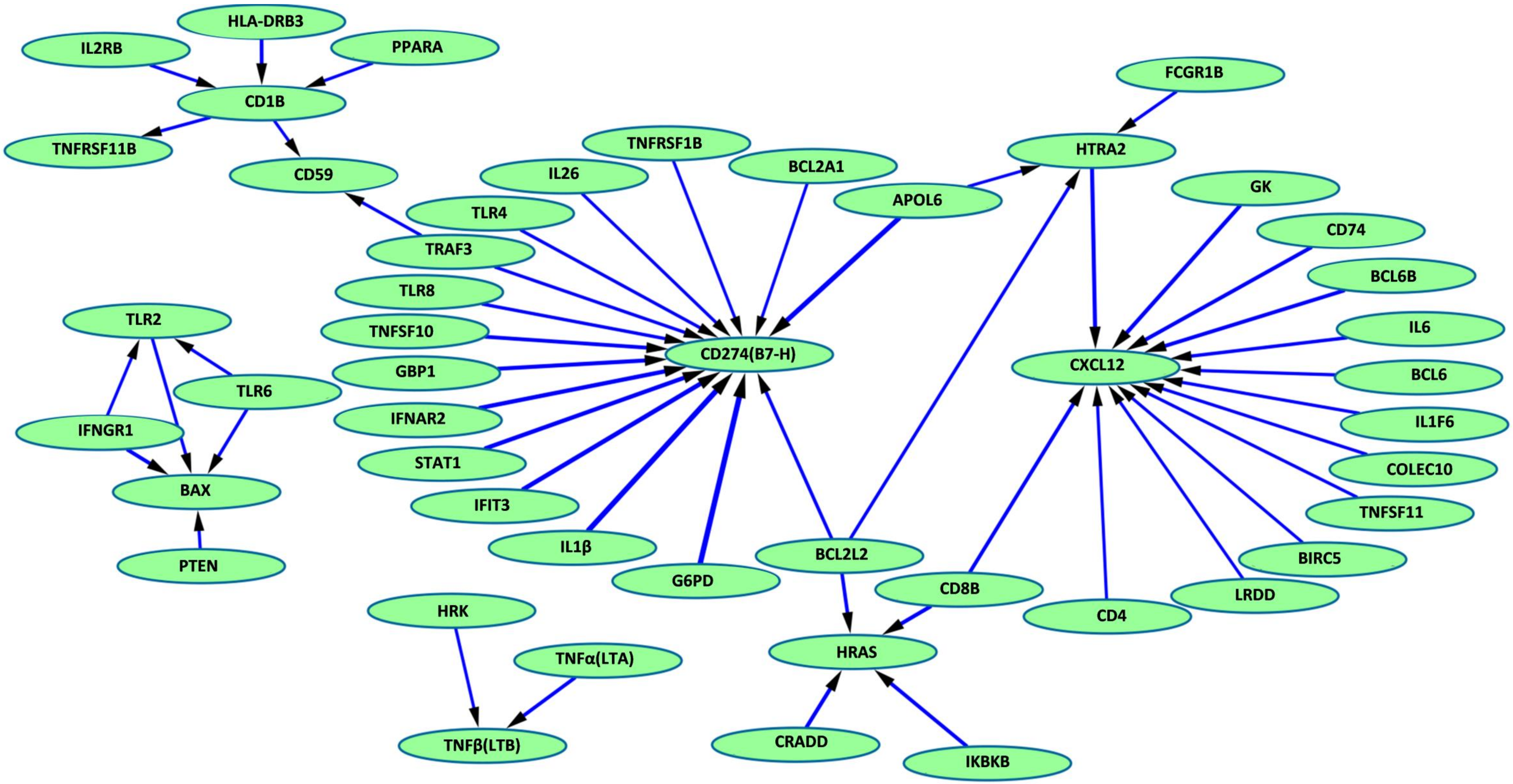
HLA-DRB3
IL2RB
PPARA
CD1B
TNFRSF11B
CD59
FCGR1B
TNFRSF1B
HTRA2
IL26
BCL2A1
APOL6
GK
TLR4
TRAF3
CD74
BCL6B
TLR8
TLR2
TNFSF10
CD274(B7-H)
IL6
CXCL12
GBP1
BCL6
TLR6
IFNGR1
IFNAR2
IL1F6
STAT1
COLEC10
BAX
IFIT3
TNFSF11
IL1β
BCL2L2
BIRC5
PTEN
G6PD
CD8B
LRDD
CD4
HRK
TNFα(LTA)
HRAS
TNFβ(LTB)
CRADD
IKBKB
