# Supplementary material for: Temporal Expression of Peripheral Blood Leukocyte Biomarkers in a Macaca fascicularis Infection Model of Tuberculosis; Comparison with Human Datasets and Analysis with Parametric/Non-parametric Tools for Improved Diagnostic Biomarker Identification
Source: PLoS One. 2016 May 26;11(5):e0154320. doi: 10.1371/journal.pone.0154320 (PMC4882019; doi:10.1371/journal.pone.0154320)
Supplement: S4 File — (PDF) [file pone.0154320.s004.pdf]

Supplementary Information S4 – Entities Shared Between NHP-TB Top 1000 Ranked Entities from Artificial Neural Network Analysis and Top 1000 Ranked Entities from Analysis of Variance (Benjamini-Hochberg False Discovery Rate Multiple testing Correction P <0.05)

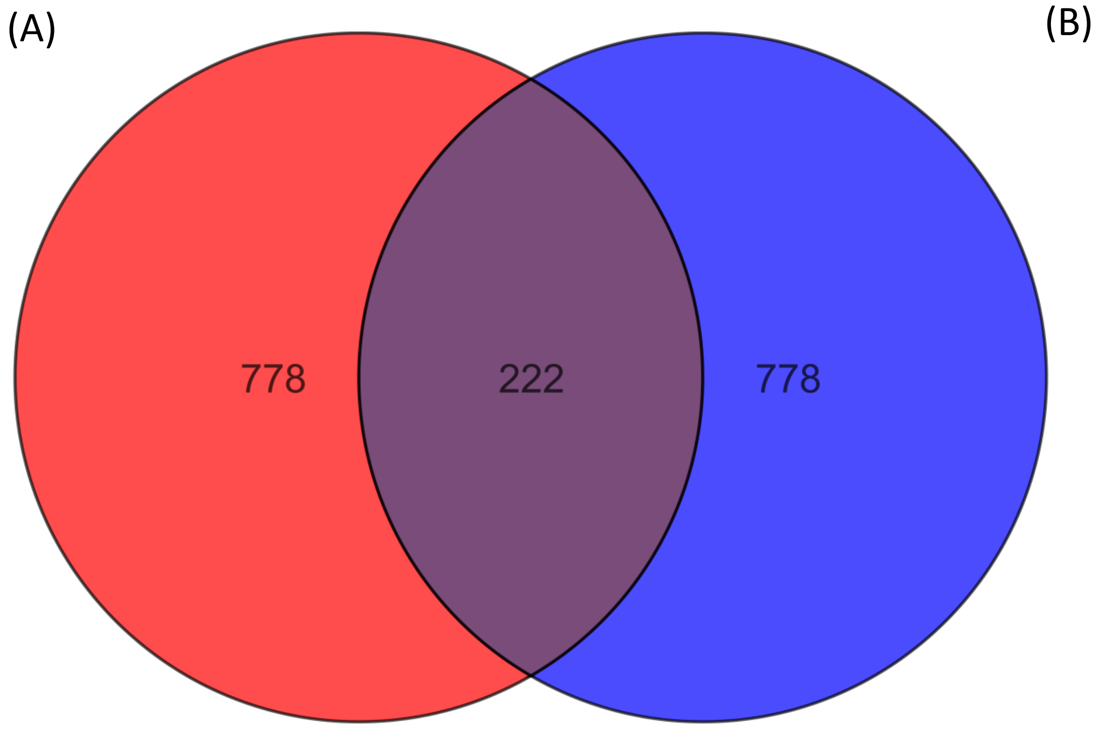

Figure A. **A –** Top 1000 ranked entities from ANN analysis, **B** - Top 1000 ranked entities from ANOVA BH-FDR analysis

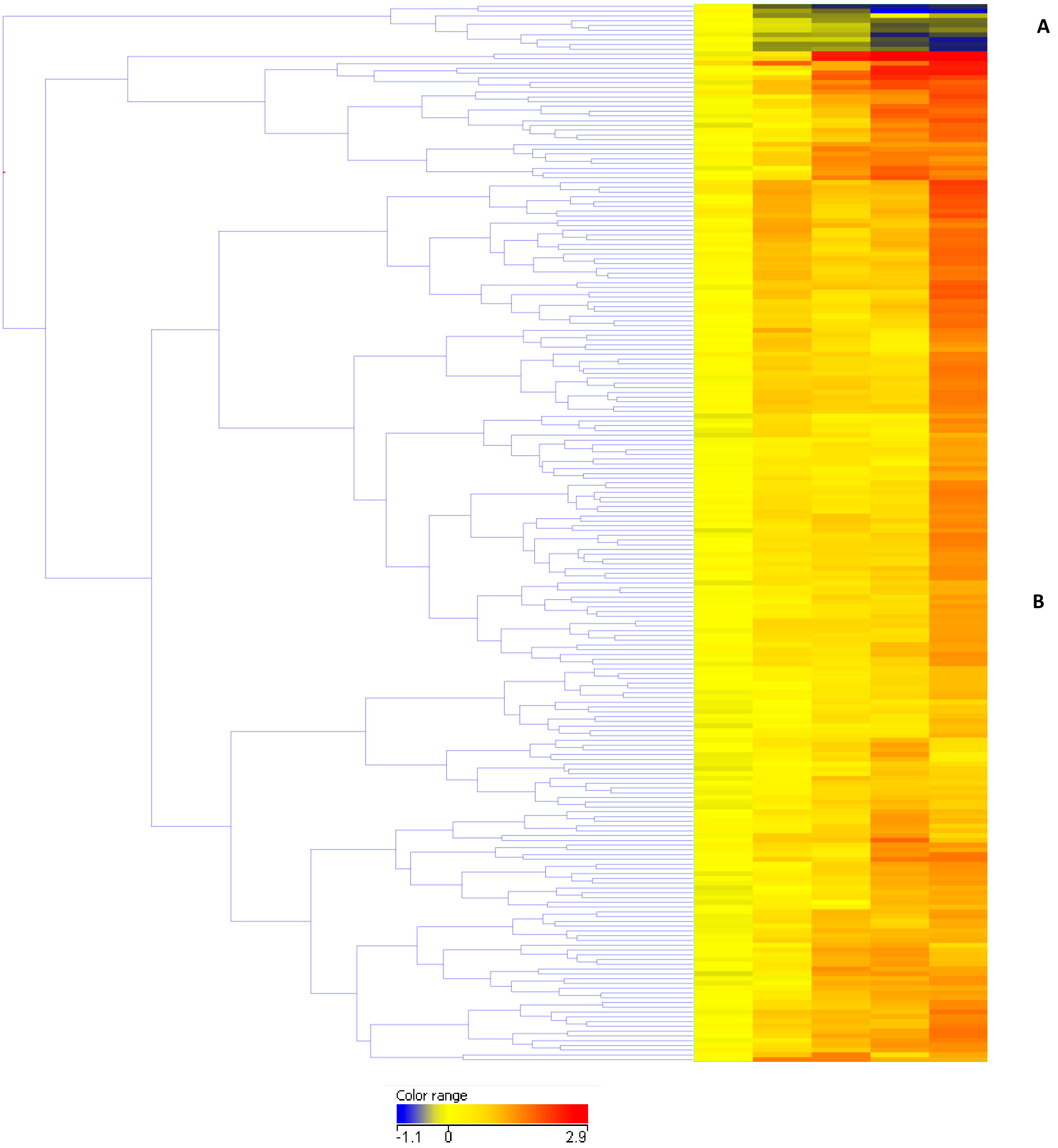

Figure B Hierarchical cluster analysis (clustering on entities) of shared entities between the T1000ANN and T1000ANOVA entity lists. A – Cluster of down-regulated entities, B – Cluster of up-regulated entities

Table A – Entities Listed in Cluster Order from Figure Above

OLIGO ID	p value Corrected BH-FDR	p value Uncorrected	GENE SYMBOL	ENTREZ ID
H300003222	3.14E-11	1.80E-11	AC008993.3-2;AL627309.15-1;FAM39B;FAM39B;AL449043.16-2	375690
H200003738	5.26E-15	4.50E-16	LNX2	222484
opHsV0400011984	7.09E-11	5.02E-11		
opHsV0400008501	3.81E-10	3.40E-10		
opHsV0400012739	9.38E-10	9.13E-10		
opHsV0400009684	6.65E-10	6.17E-10	GABRG3	2567
opHsV0400002669	6.07E-11	4.12E-11	LRRC19;IFT74	80173;64922
opHsV0400000870	1.30E-18	4.68E-20	HSPA14	51182
opHsV0400010910	3.63E-13	6.37E-14		
H300012835	6.02E-11	4.04E-11	FAM13C;PHYHIPL	220965;84457
H200005495	8.81E-21	1.59E-22	GBP1	2633
opHsV0400008241	1.23E-22	5.52E-25	RP4-644F6.3	
opHsV0400001796	9.69E-10	9.47E-10		
H300020304	2.01E-15	1.40E-16	PLAC8	51316
opHsV0400008242	7.06E-16	3.77E-17	GBP1;GBP3;RP4-644F6.3	2635;2633
H300012822	7.72E-12	3.14E-12	SOD2;FAM177B	6648;400823
H300020694	6.56E-16	2.95E-17	FCER1G	2207
H200011967	1.91E-11	9.88E-12	JAK2	3717
H300003686	3.18E-10	2.75E-10	AC092905.10	
H300000274	1.04E-11	4.80E-12	SAMD9L	219285
H300015777	4.88E-12	1.71E-12	FYB	2533
H300002527	6.48E-14	8.46E-15	GLRXL	100132510
H200003262	5.50E-15	4.95E-16	RNASE6	6039
H300015894	1.48E-21	1.60E-23	CARD16	114769
H200007677	7.06E-16	3.82E-17	TMEM49	81671
opHsV0400008767	1.48E-15	9.31E-17	CLIC1;AC078875.37;CLIC1;CLIC1	1192;1192;1192
H200001091	2.73E-13	4.30E-14	CREG1	8804
opHsV0400000601	5.08E-15	4.12E-16	EIF4E3	317649
H200008677	1.46E-16	5.93E-18	SERPINB1	1992
H300018513	3.53E-11	2.07E-11	COPEB	1316
H300008845	5.31E-13	1.15E-13	HLA-B;HLA-B;HLA-C;HLA-C;HLA-C	3106;3107
H300013862	4.72E-13	9.78E-14	MVP	9961
opHsV0400003325	1.14E-10	8.85E-11		
H200007265	1.15E-11	5.47E-12	SPI1	6688
H300008499	1.48E-21	2.00E-23	IRF1	3659
H300009187	7.16E-19	2.26E-20	PSMB9;PSMB9;PSMB9	5698;5698;5698
H300019720	1.66E-10	1.33E-10	LGALS3BP	100133842;3959
H300017417	1.06E-15	6.22E-17		
H300004895	1.08E-11	5.03E-12	UBE2N	7334

H200003625	2.73E-13	4.24E-14	INTS12	57117
H200001910	7.87E-12	3.26E-12	RHOBTB3	22836
H300008369	9.56E-12	4.22E-12		
H200006335	9.56E-12	4.20E-12	INA	9118
opHsV0400001663	8.83E-11	6.52E-11	ARSI	340075
opHsV0400007161	1.90E-12	5.44E-13		
opHsV0400012779	1.73E-11	8.86E-12	Z99774.1	100129936
H300007547	3.48E-10	3.04E-10	TSHZ2	128553
H300012451	1.30E-10	1.02E-10	DHPS	1725
H300022424	3.55E-10	3.13E-10	C9orf86	55684
H300004414	9.11E-13	2.18E-13	HIST2H2AC;AL133244.1;BOLA1;LTBP1	4052;51027;8338
opHsV0400001790	4.71E-13	9.55E-14	AL355075.6-1	100131227
opHsV0400007672	1.57E-12	4.18E-13	TBX20	57057
opHsV0400012428	1.63E-12	4.42E-13		
H200003225	2.78E-13	4.64E-14	ST8SIA5	29906
H200011551	5.40E-14	6.32E-15	UHRF1;PDE3A	29128;100133565;5139
H200012165	1.97E-10	1.61E-10	SENP3	26168
H300003763	3.29E-14	3.56E-15	NOLA3	55505
opHsV0400009404	5.74E-12	2.09E-12		
opHsV0400000644	2.11E-12	6.46E-13	PRKACG	5568
H200008415	7.16E-19	2.17E-20	MYL12B	103910;642076
opHsV0400006379	3.99E-11	2.49E-11	KIAA1143	57456
opHsV0400000205	2.76E-14	2.61E-15	SAG	6295
H200001879	8.89E-11	6.64E-11	SGK493	91461
H200008131	5.24E-13	1.11E-13	ELL2	22936
opHsV0400000132	4.61E-11	2.93E-11	AC000403.1	
H300009117	6.67E-12	2.52E-12	OSR2	116039
H300001033	1.85E-13	2.67E-14	LUZP2	338645
H300008068	3.99E-11	2.50E-11	TICAM2	353376;51014
opHsV0400010383	1.91E-12	5.67E-13	C2orf34	79823
opHsV0400002124	4.48E-12	1.55E-12	ZNF716	441234
H300015082	7.72E-12	3.16E-12	SLC25A16	8034
opHsV0400003262	5.17E-11	3.40E-11		
opHsV0400003632	1.06E-09	1.06E-09		
H300012970	3.81E-11	2.30E-11	ACIN1	22985
H300000980	6.46E-12	2.42E-12		
H200004368	8.89E-11	6.61E-11	STATH	6779
H300001262	1.61E-10	1.28E-10	PK4P;AC005042.2	8502;100129029
H300009289	1.34E-12	3.43E-13	TSSK4	283629
H300022819	1.65E-11	8.25E-12	CNPY3;AL353716.18	10695
H200016347	3.08E-10	2.65E-10	FOXD3	27022
H300021741	1.42E-11	6.89E-12	CDH23	64072
H200011284	1.96E-12	5.91E-13	ZNF81	347344
opHsV0400013404	2.78E-10	2.36E-10	AC073308.4;AC021914.7-1;AF347015.1-3	

H200006010	8.16E-13	1.87E-13	ILF2	3608
H200010400	2.93E-11	1.65E-11	CLCN5	1184
H300021767	3.85E-12	1.23E-12	SEMA6A	57556
opHsV0400002504	6.99E-12	2.74E-12	RPH3AL	9501
H300021066	1.14E-10	8.86E-11	CENPN	55839
opHsV0400000373	8.09E-13	1.79E-13	CSAD	51380
H300006623	2.78E-11	1.51E-11	XYLT1	64131
opHsV0400004693	7.39E-11	5.29E-11	AC011503.4	
H200012627	2.78E-11	1.52E-11	MTMR10	54893
H200004226	3.97E-12	1.31E-12	PPIE	10450
H300002254	4.62E-11	2.96E-11		
H300006575	6.91E-11	4.85E-11	SALL3	27164
H200010367	2.99E-14	2.96E-15	RYR1	6261
opHsV0400000802	7.34E-10	6.84E-10		
H300012464	3.89E-12	1.26E-12	LPHN2	23266
H200006161	3.89E-10	3.50E-10	PLCG2	5336
opHsV0400007061	1.02E-11	4.60E-12	RNPS1	10921;643446
opHsV0400003210	9.11E-13	2.22E-13	C19orf54	284325
H200005853	7.50E-12	2.97E-12	KIF20A	10112
H200011726	2.14E-11	1.13E-11	RRAGC	64121
H300004948	1.83E-12	5.03E-13	FAM21C;FAM21A;FAM21B;AL672187.12-2;PARG	55747;387680;253725;652697;653450;653450;8505
H200014587	1.89E-10	1.52E-10	PSMD5	5711
opHsV0400000937	5.63E-12	2.03E-12	TNP2	7142
H300019346	5.78E-14	7.29E-15	MICAL2	9645
opHsV0400013389	3.82E-10	3.42E-10		
opHsV0400000220	3.58E-10	3.18E-10	FTHL17	53940
H200004995	4.02E-13	7.24E-14	JAK1	3716
H300022550	1.11E-11	5.19E-12	WAC	51322
H200014520	2.78E-13	4.59E-14	C8orf39;RBM12B	389677
H300017529	1.91E-12	5.63E-13	KIR2DL1;KIR2DL3	3802;768329;3804
H300000014	4.19E-13	7.73E-14	AL662820.6;MYL8P;MYL8P	442204
H300013136	1.92E-11	1.00E-11	FCN1	2219
H300001614	6.68E-12	2.56E-12	VKORC1L1	154807
opHsV0400006468	1.02E-11	4.56E-12	FAM49A	81553
opHsV0400010145	1.05E-09	1.04E-09	PNMA5	114824
opHsV0400006219	6.97E-12	2.70E-12	LBX1	10660
H300011553	2.40E-11	1.28E-11	MRAP;URB1	9875;56246
H300004243	5.52E-10	5.10E-10	ZNF835	90485
opHsV0400010754	8.29E-12	3.51E-12	PSMD6	9861
opHsV0400004499	2.49E-10	2.09E-10	IBRDC2	255488
H300018539	1.08E-10	8.33E-11	CTSA	5476
H200001695	1.44E-11	7.09E-12	BAZ1A	11177
H200000400	3.42E-11	1.99E-11	ARHGAP25	9938

H300019233	3.81E-11	2.30E-11	POLDIP3	84271
H300019257	9.56E-11	7.27E-11	PAK4	10298
opHsV0400000082	3.28E-10	2.85E-10	MYL7	58498
H200001464	1.72E-11	8.70E-12	SEMA4A	64218
H300020700	5.02E-10	4.61E-10	FBXW5	54461
H200015518	1.73E-11	8.87E-12	NKX2-8	26257
opHsV0400003366	7.36E-10	6.90E-10	AC068580.3	
H300004594	4.24E-12	1.45E-12	UBE2L3;UBE2L7P;PDE3A	7332;5139;7332
H300006326	3.81E-11	2.28E-11	NLRP8	126205
opHsV0400008706	3.36E-12	1.06E-12		
opHsV0400005931	1.58E-10	1.25E-10		
opHsV0400005510	2.99E-13	5.12E-14	INPP5A	3632
H300009401	4.42E-10	4.04E-10		
H300003972	2.83E-11	1.57E-11	G6PD	2539
H300022660	4.44E-13	8.80E-14	LRRC20	55222
H300013172	9.82E-10	9.69E-10	REST	5978
H200001562	9.16E-12	3.96E-12	DMAP1	55929
opHsV0400006959	4.24E-12	1.45E-12	CASP12	120329
opHsV0400007011	5.79E-11	3.84E-11	SBDS	100133419;51119
opHsV0400009616	1.30E-11	6.24E-12		
opHsV0400010878	6.02E-11	4.02E-11	TRIM25	7706
H200003348	2.11E-10	1.74E-10	SAP18	10284
H300004772	1.90E-12	5.49E-13		
H300021172	8.16E-13	1.91E-13	HOP	84525
H300017182	2.62E-10	2.21E-10	CREB3L3	84699
H200007889	3.81E-11	2.32E-11	BST1	683
H200002021	8.40E-11	6.10E-11	ACSL5	51703
H300008569	9.55E-11	7.22E-11	UBE2L3;UBE2L7P;RP11-223E19.1;PDE3A	7332;5139;7332
opHsV0400003535	7.81E-10	7.39E-10	TUBA3C;TUBA3D;TUBA3E	113457;7278;112714;7278;113457
H200013772	1.05E-09	1.04E-09	HIST1H3H;HIST1H2BO	8348;8353;8354;8355;8356;8357;8350;8358;8351;8968;8352
opHsV0400003329	4.98E-11	3.21E-11	NAALADL1	10004
H300018835	1.94E-10	1.57E-10	PXN	5829
opHsV0400006851	1.13E-11	5.35E-12	CNN2	1265
H300018521	3.96E-10	3.59E-10	ALPK1	80216
opHsV0400012071	2.83E-11	1.57E-11	PHF21B	112885
opHsV0400004591	2.73E-13	4.05E-14	PLXNB2	23654
opHsV0400012806	3.12E-14	3.24E-15		
opHsV0400004828	2.87E-10	2.46E-10	APBB1IP	54518
H200011182	2.34E-10	1.95E-10	AYTL1	54947
opHsV0400001073	6.48E-11	4.44E-11	APBB1IP	54518
opHsV0400006519	3.50E-10	3.08E-10	AC133644.3-2;AC068491.4;AC133644.1	
H300021493	9.70E-10	9.53E-10	RHOG	391
H200006801	4.22E-13	7.98E-14	LYN	4067

H300002628	8.43E-10	8.05E-10	LAP3	51056
H300017159	2.20E-12	6.84E-13	S100A4	6275
H200013144	1.04E-11	4.79E-12	CST3	1471
H300010795	7.66E-10	7.21E-10	PLXNB1	5364
H200007999	6.64E-11	4.58E-11	GBP2	2634
H200000672	8.98E-10	8.70E-10	HOXB2	3212
H300007517	8.96E-10	8.64E-10	OR14K1	343170
opHsV0400002461	4.16E-12	1.39E-12	ANXA2;TRIM2	23321;302
H200010299	6.22E-12	2.30E-12	CYBB	1536
H200000549	2.53E-11	1.35E-11	PPBP	5473
H200000680	6.62E-14	8.95E-15	LDHA;MMS19	3939;64210
H200002726	7.16E-19	1.83E-20	TLN1	7094
H200013951	1.60E-11	7.93E-12	CALCOCO2	10241
H200004008	3.90E-11	2.41E-11	RNF24	11237
H200011661	7.90E-10	7.50E-10	UBL5	59286
H300005394	2.94E-11	1.67E-11	RP4-681N20.1	
H300013468	4.44E-13	8.74E-14	DMXL2	23312
H300008312	1.03E-10	7.85E-11	RPL41;RBM9;DGKI;HERC2;IL6ST	23543;8924;3572;9162;6171
opHsV0400000356	8.04E-12	3.37E-12	RHOA	387
H300016502	8.56E-11	6.28E-11	C6orf25;C6orf25;C6orf25	80739;80739;80739
H200018455	8.91E-10	8.55E-10		
opHsV0400006965	7.32E-11	5.21E-11	TOP1;FMO4;PIWIL3	2329;440822;7150
opHsV0400002597	9.14E-12	3.91E-12	ATP6AP2	10159
H300013028	6.07E-11	4.13E-11	CHD9	80205
H200002230	1.99E-10	1.63E-10	SH2B3	10019
opHsV0400000981	9.55E-11	7.23E-11	AC007842.1	54458
H200000014	7.81E-11	5.63E-11	PTAFR	5724
opHsV0400003327	2.84E-10	2.42E-10	MARCH1	55016
H200012133	7.66E-12	3.07E-12	OASL	8638
H300018826	3.75E-11	2.21E-11	ST3GAL4	6484
H300010861	8.16E-13	1.90E-13	KIAA1618	57714
H200018030	5.78E-14	7.15E-15	CCRL2	9034
H300018648	1.90E-12	5.45E-13	HNRNPC	3183
H200004856	5.13E-14	5.78E-15	PARP9	83666
H300004907	3.10E-15	2.37E-16	IFITM1;IFITM2;IFITM3	10410;10581;8519
H300013722	2.28E-10	1.89E-10	STAT1	6772
H300015308	5.63E-12	2.02E-12	STAT1	6772
opHsV0400008258	6.71E-11	4.65E-11	DAZAP2	9802
H300004068	3.85E-11	2.36E-11	RPL41;RBM9;DGKI;HERC2;IL6ST	23543;8924;3572;9162;6171
H200010375	1.53E-12	4.00E-13	TAF10	6881
H300001620	3.35E-11	1.93E-11	HLA-DRA;HLA-DRA	3122
H200011822	8.53E-11	6.22E-11	AMH	268
H200003480	9.81E-13	2.43E-13	CYP46A1	10858
H300008419	2.85E-11	1.59E-11	AC087289.9;UNK	85451;100129946

H200008434	6.78E-11	4.74E-11	KPNB1	3837
H300011509	1.30E-10	1.02E-10	C20orf26	26074
H200013157	2.01E-15	1.45E-16	IGSF6;METTL9	10261;51108
H200016456	5.13E-11	3.35E-11	GTF2B	2959
H200011433	9.26E-14	1.29E-14	SNX10	29887
H300001429	4.99E-11	3.24E-11	S100A11;DPY19L2;AC007683.5;AC005400.1	729659;730278;6282;283417
H200008517	4.00E-10	3.64E-10	ATF4;AC015801.2;RNF157	468;643159;114804
H300020334	4.19E-11	2.64E-11	TAGAP	117289
H300018424	1.01E-12	2.55E-13	CLK1	1195
