# Supplementary material for: Temporal Expression of Peripheral Blood Leukocyte Biomarkers in a Macaca fascicularis Infection Model of Tuberculosis; Comparison with Human Datasets and Analysis with Parametric/Non-parametric Tools for Improved Diagnostic Biomarker Identification
Source: PLoS One. 2016 May 26;11(5):e0154320. doi: 10.1371/journal.pone.0154320 (PMC4882019; doi:10.1371/journal.pone.0154320)
Supplement: S5 File — (PDF) [file pone.0154320.s005.pdf]

Supplementary Information S5 – Heat maps of Expression Patterns of the 30 Preferred Biomarker entities in Previously Published Human Datasets; Figure (A) GSE19439 and Figure (B) GSE28623

(A)

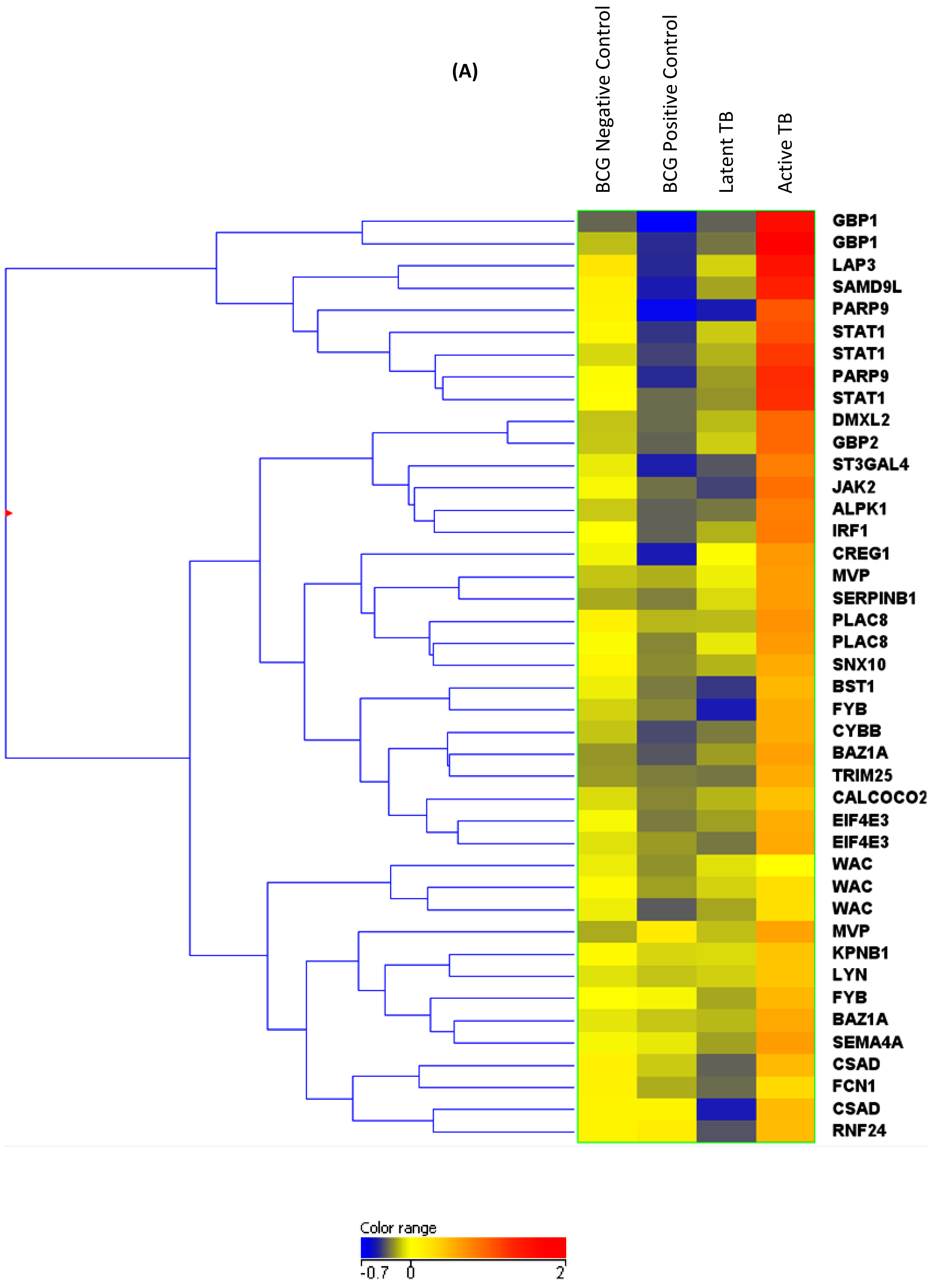

(B)

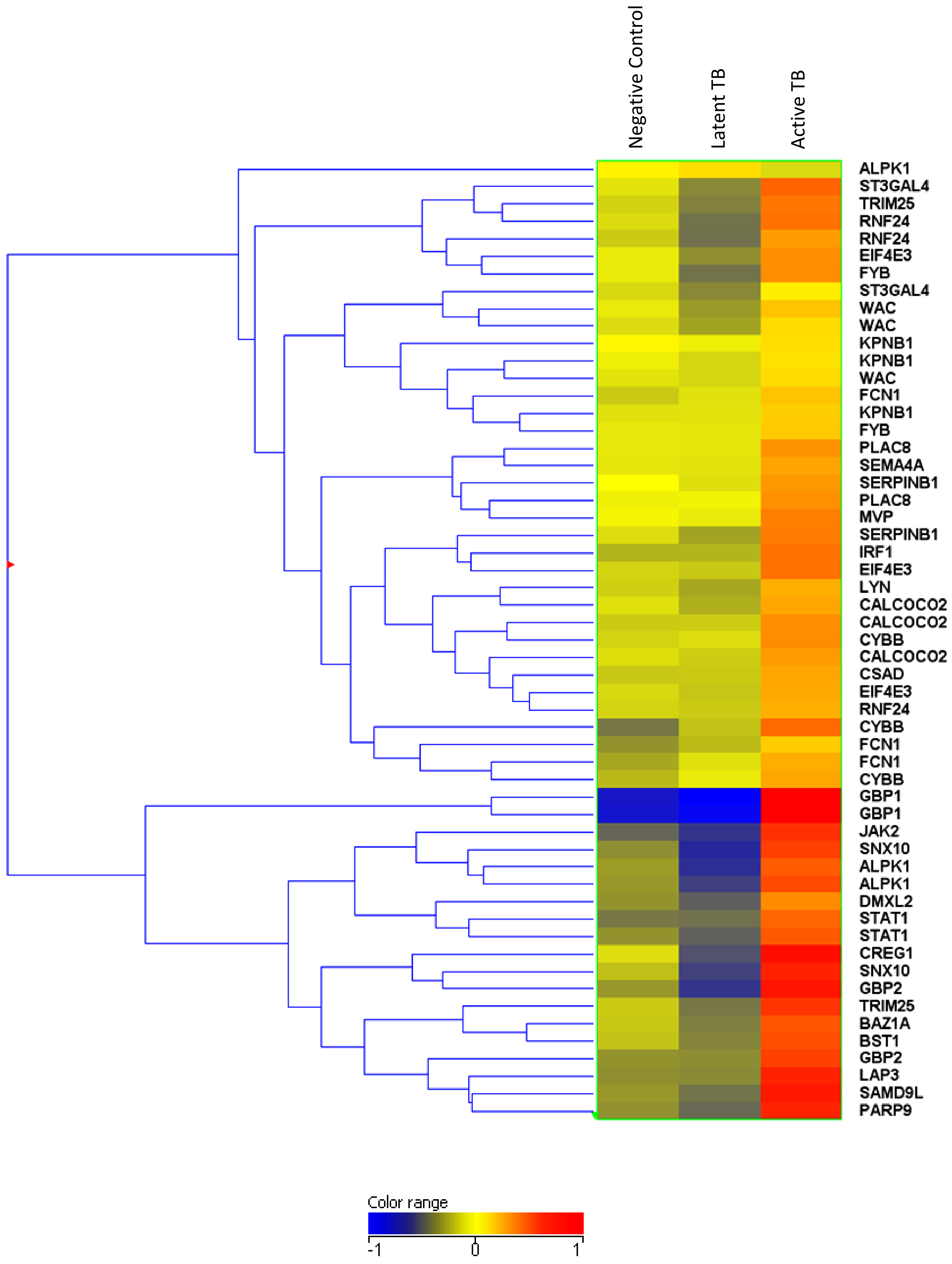
Negative Control
Latent TB
Active TB
ALPK1
ST3GAL4
TRIM25
RNF24
RNF24
EIF4E3
FYB
ST3GAL4
WAC
WAC
KPNB1
KPNB1
WAC
FCN1
KPNB1
FYB
PLAC8
SEMA4A
SERPINB1
PLAC8
MVP
SERPINB1
IRF1
EIF4E3
LYN
CALCOCO2
CALCOCO2
CYBB
CALCOCO2
CSAD
EIF4E3
RNF24
CYBB
FCN1
FCN1
CYBB
GBP1
GBP1
JAK2
SNX10
ALPK1
ALPK1
DMXL2
STAT1
STAT1
CREG1
SNX10
GBP2
TRIM25
BAZ1A
BST1
GBP2
LAP3
SAMD9L
PARP9
Color range
-1
0
1
