# Supplementary material for: Temporal Expression of Peripheral Blood Leukocyte Biomarkers in a Macaca fascicularis Infection Model of Tuberculosis; Comparison with Human Datasets and Analysis with Parametric/Non-parametric Tools for Improved Diagnostic Biomarker Identification
Source: PLoS One. 2016 May 26;11(5):e0154320. doi: 10.1371/journal.pone.0154320 (PMC4882019; doi:10.1371/journal.pone.0154320)
Supplement: S6 File — (PDF) [file pone.0154320.s006.pdf]

Supplementary Information S6 – Line graph depiction of qPCR analyses of key differentially expressed entities from Chinese and Mauritian NHP datasets. Average data values across all animals in the groups and slide replicates with standard deviation are given.

(A)

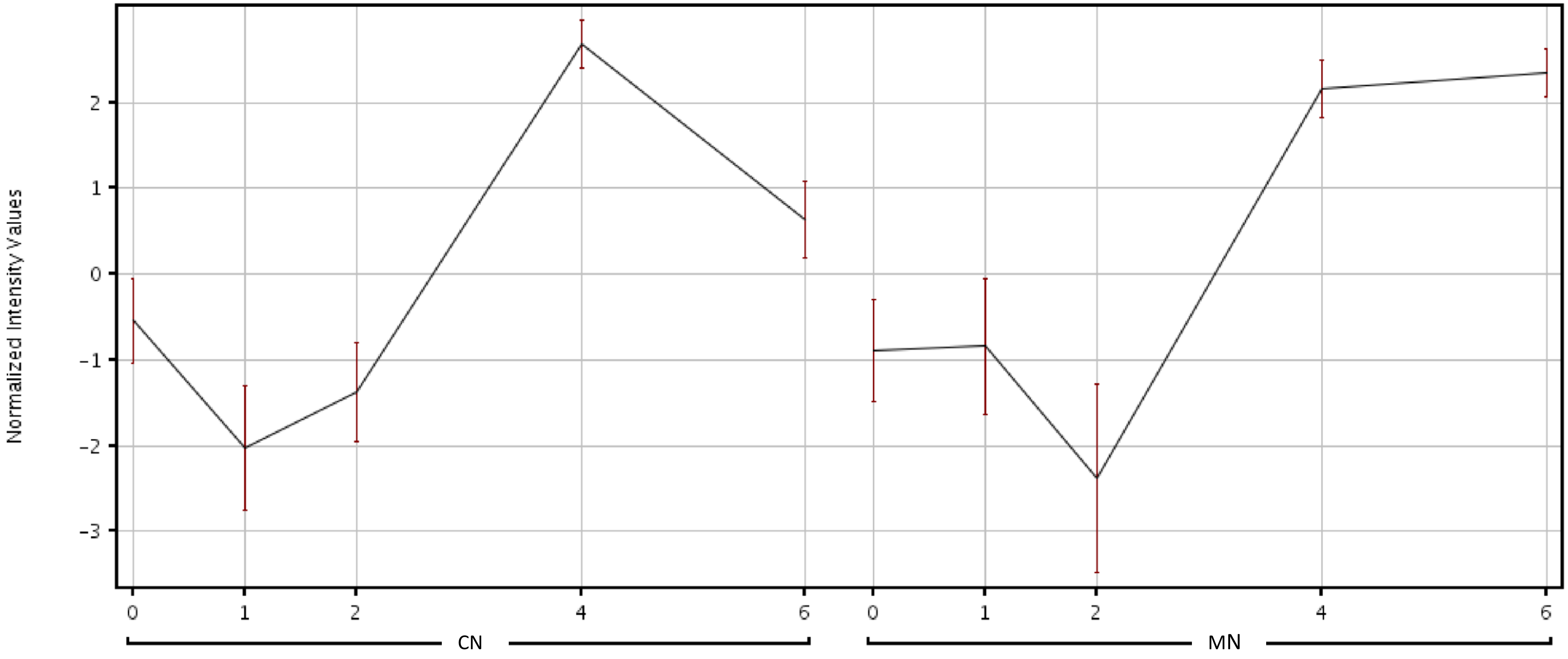

CD163

(B)

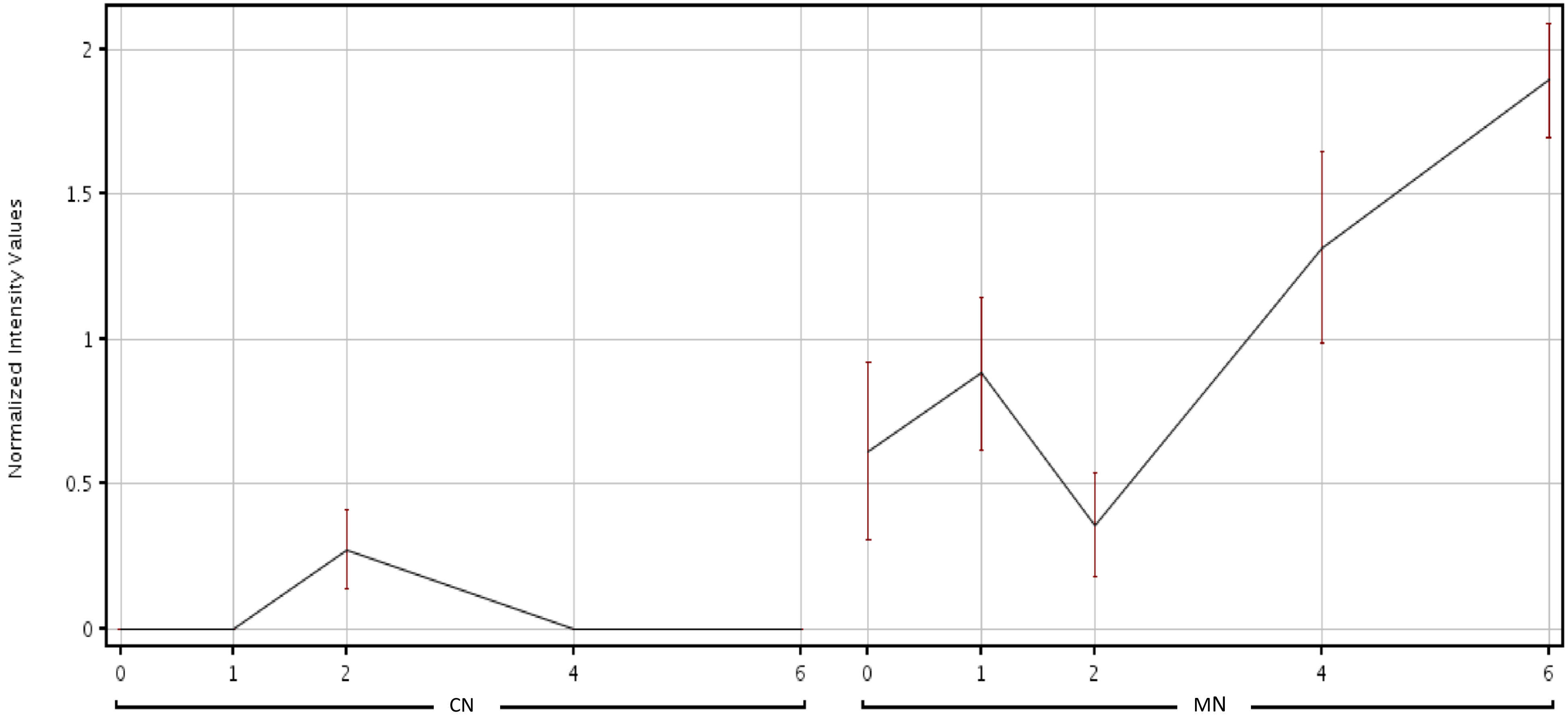

CD33

(C)

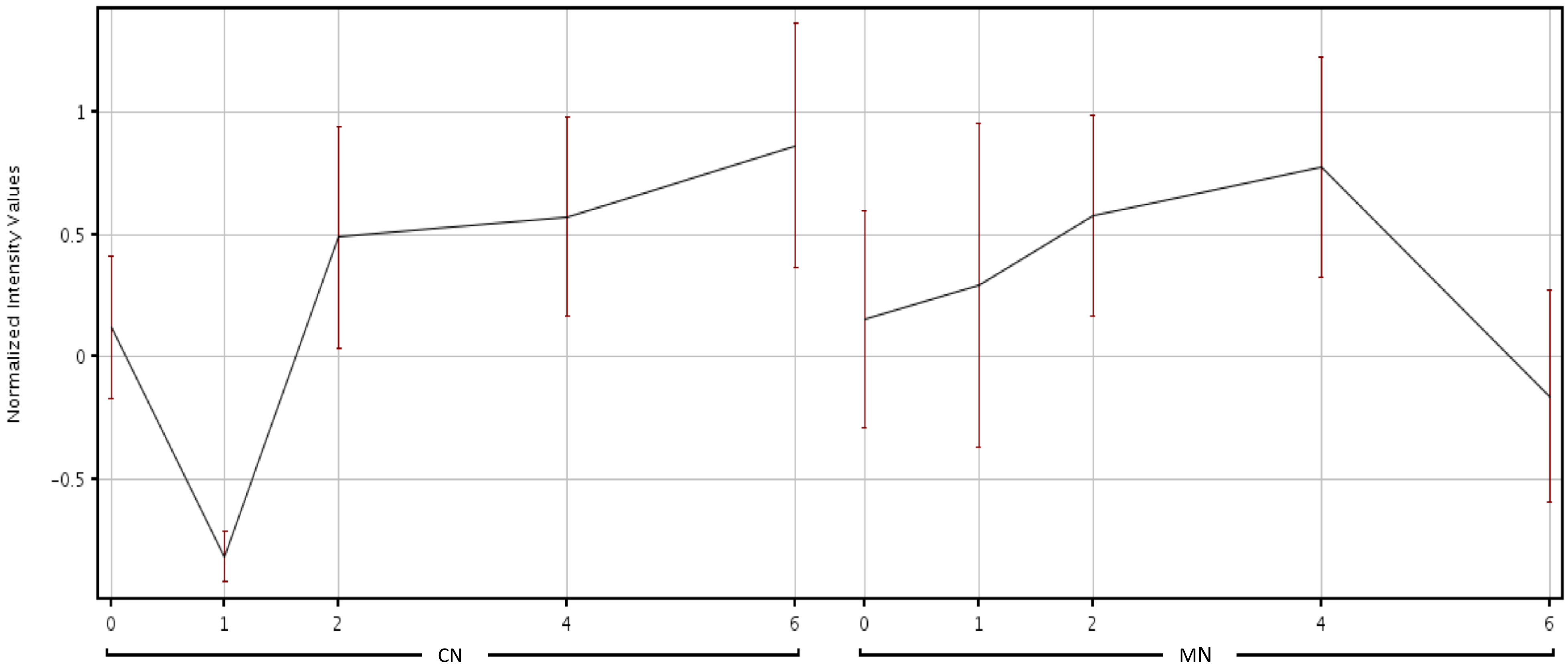

CD244

(D)

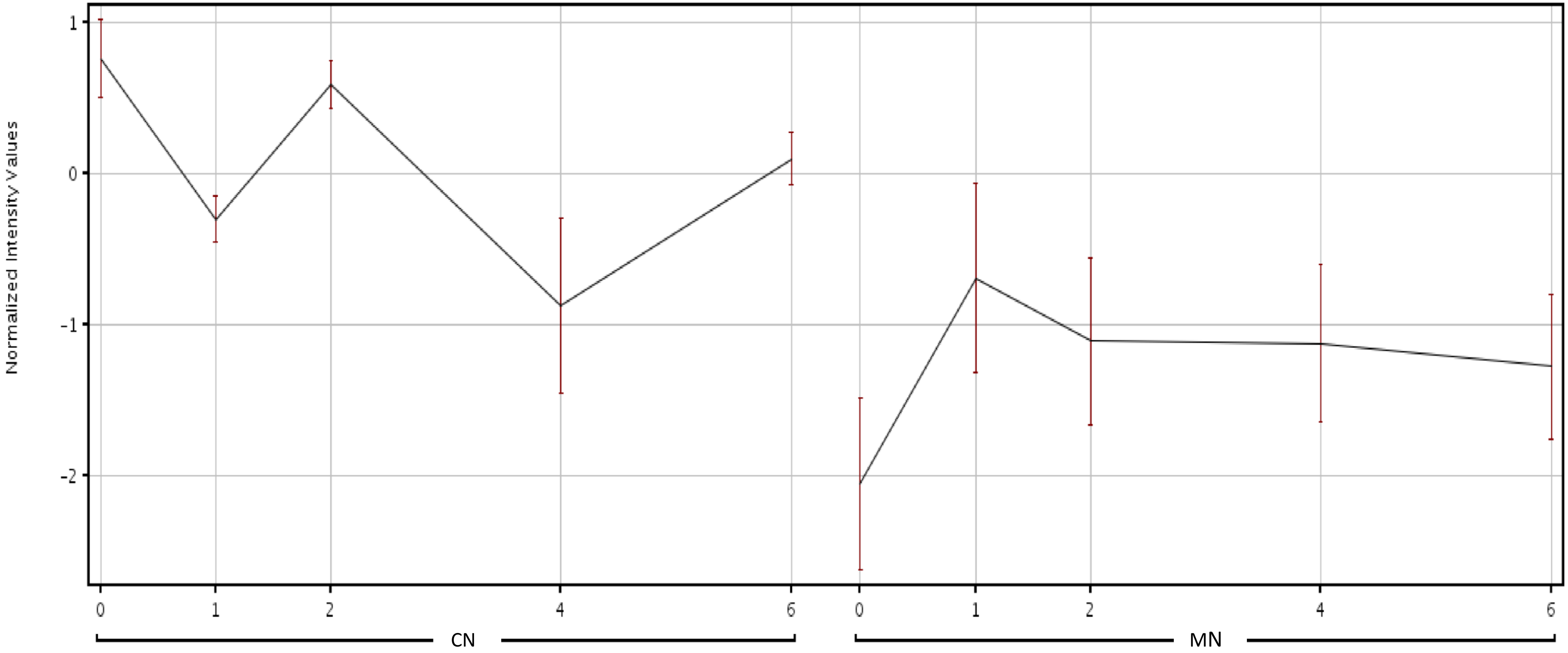

CD4

(E)

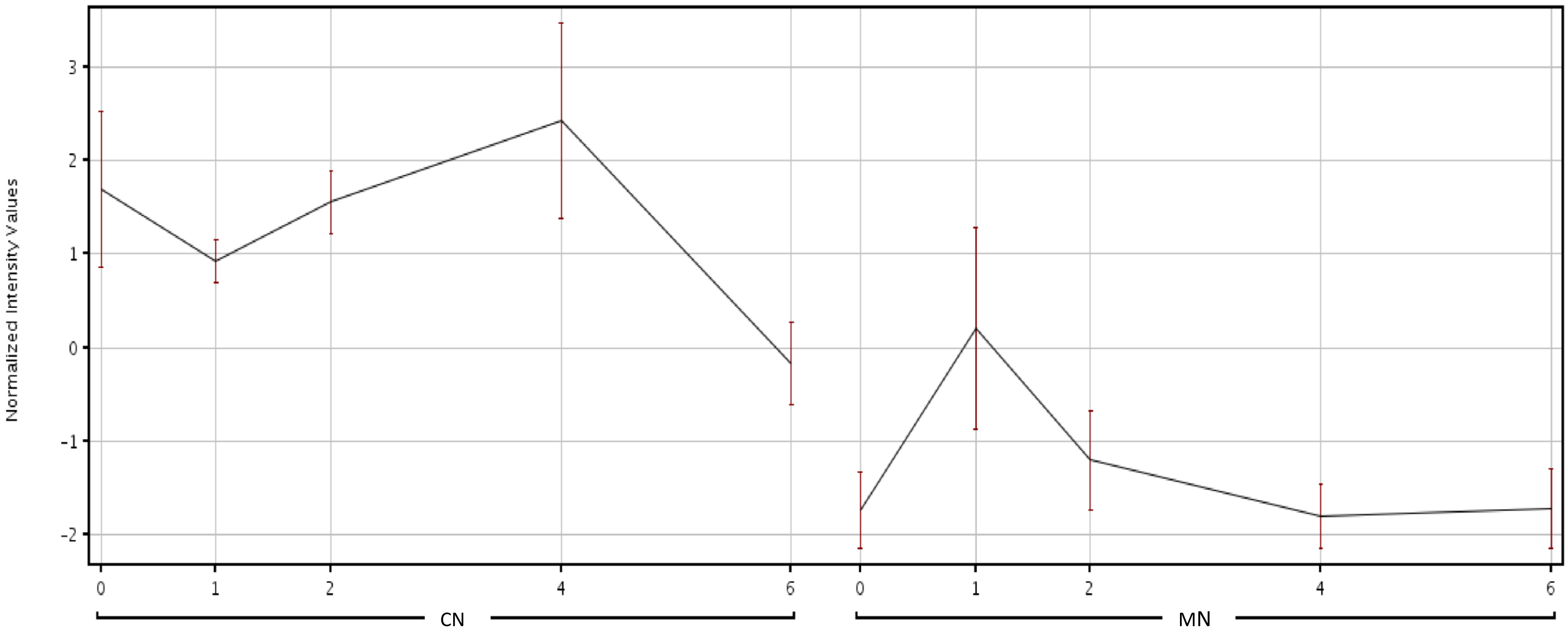

CD8A

(F)

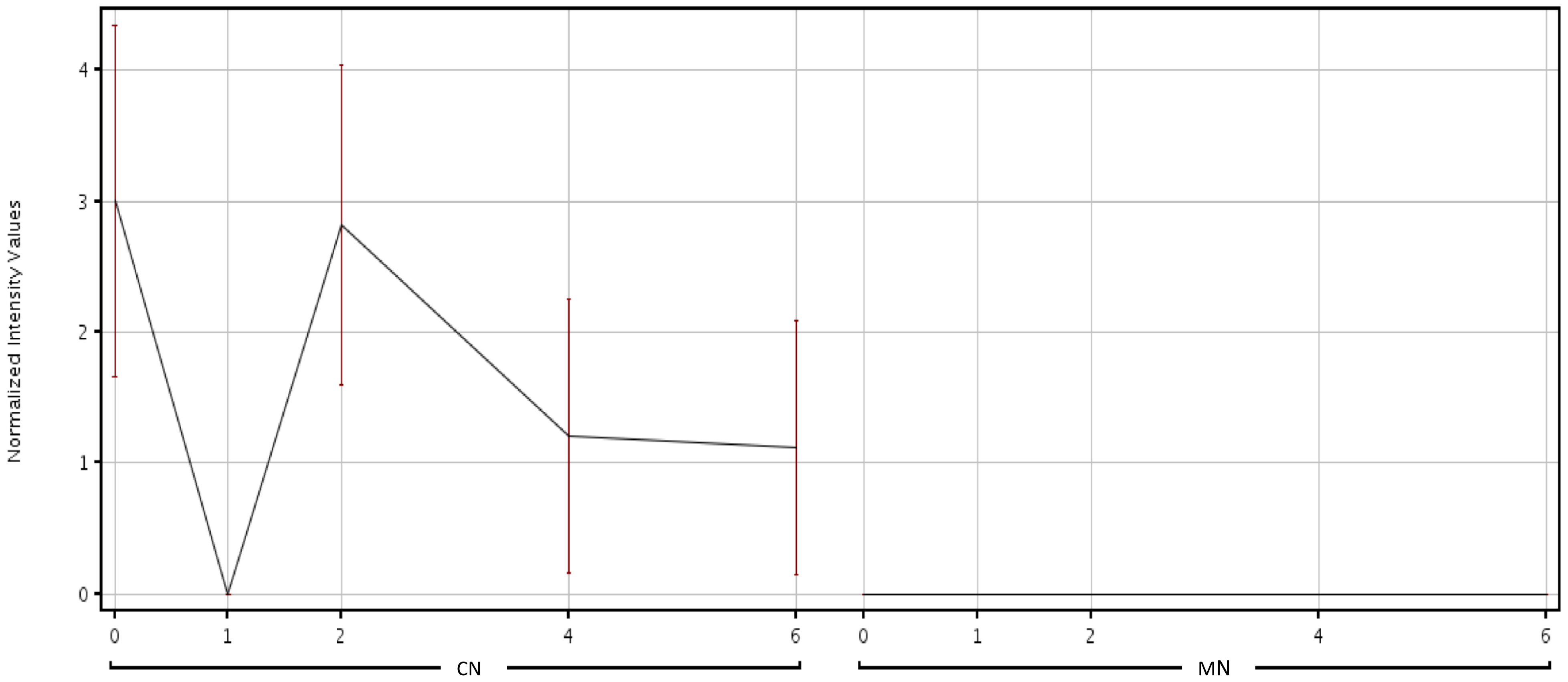

CD8B

(G)

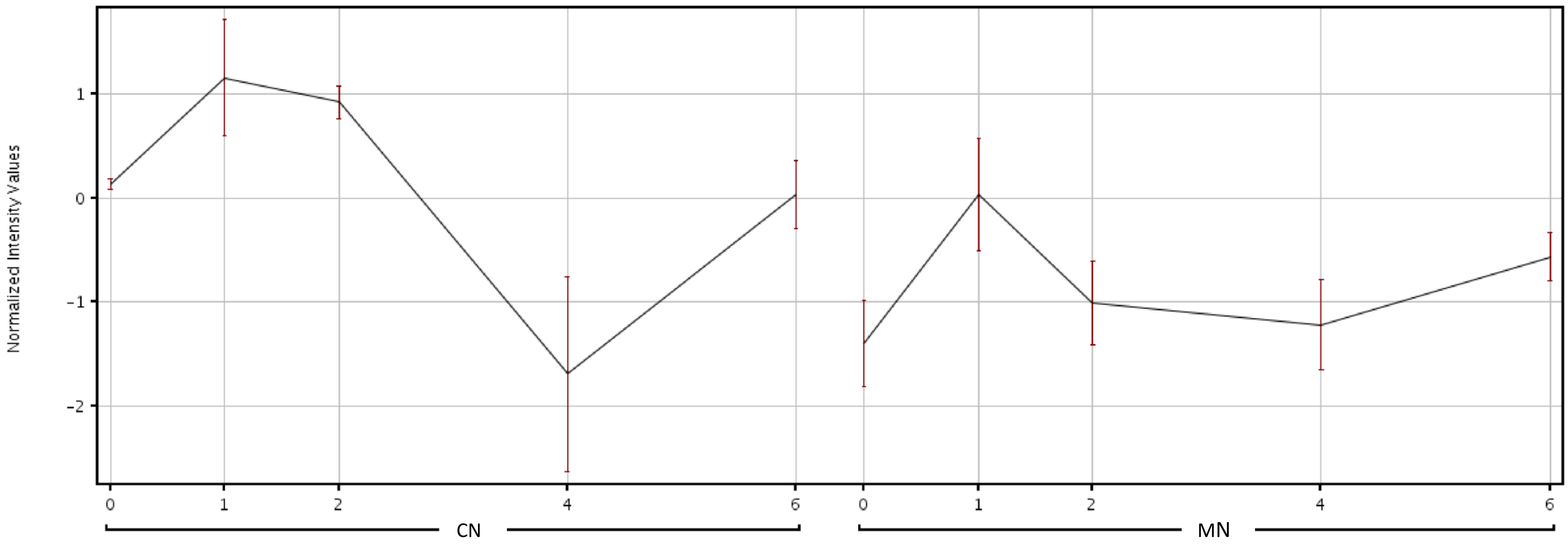

CD3D

(H)

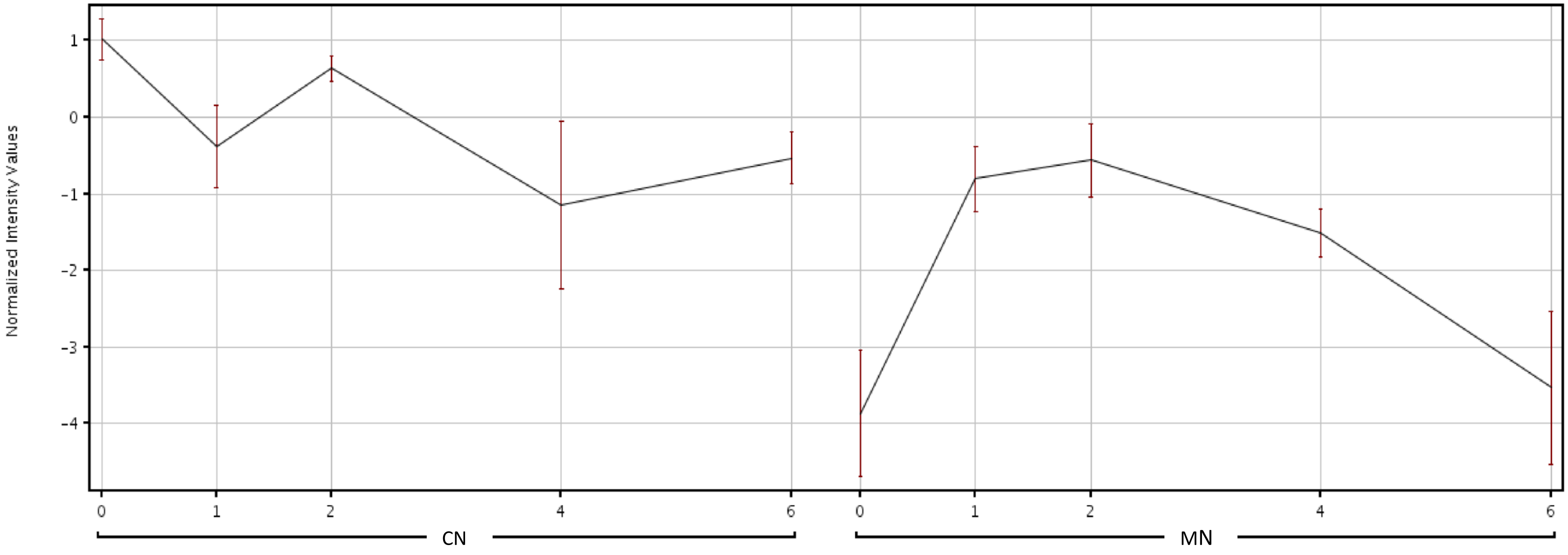

CD3E

(I)

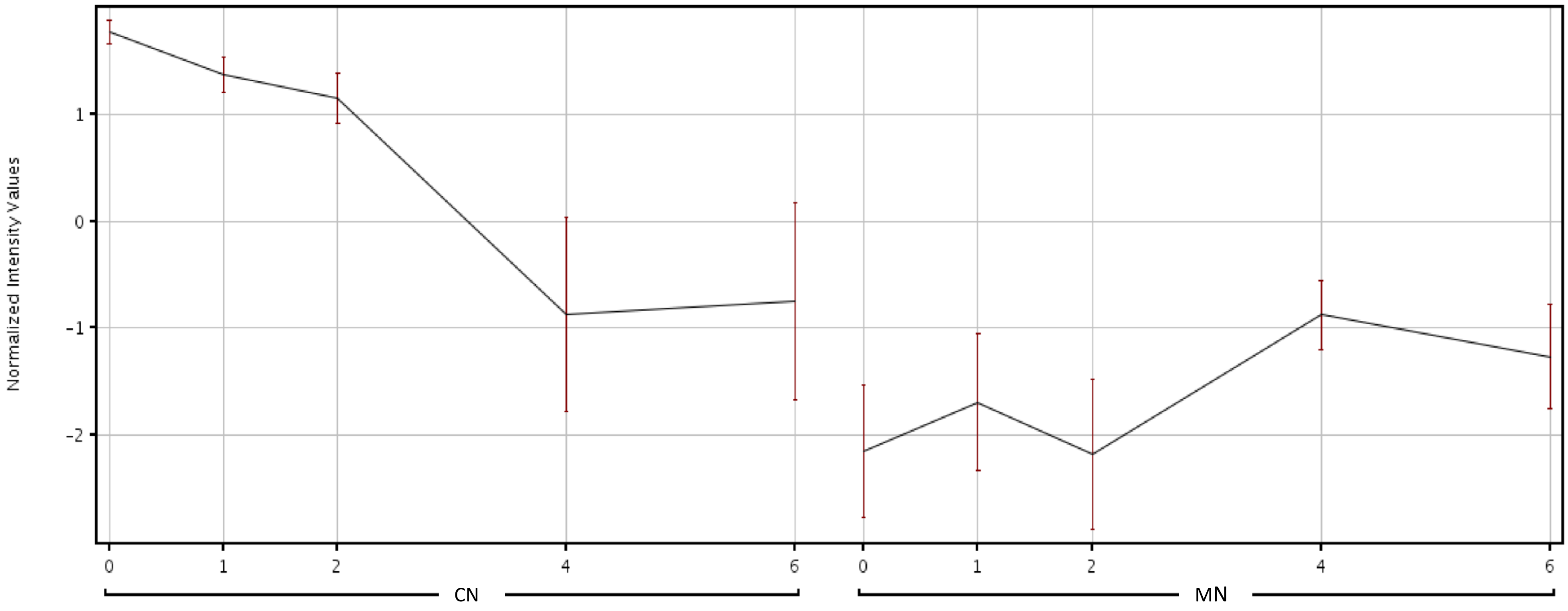

CD3Z

(J)

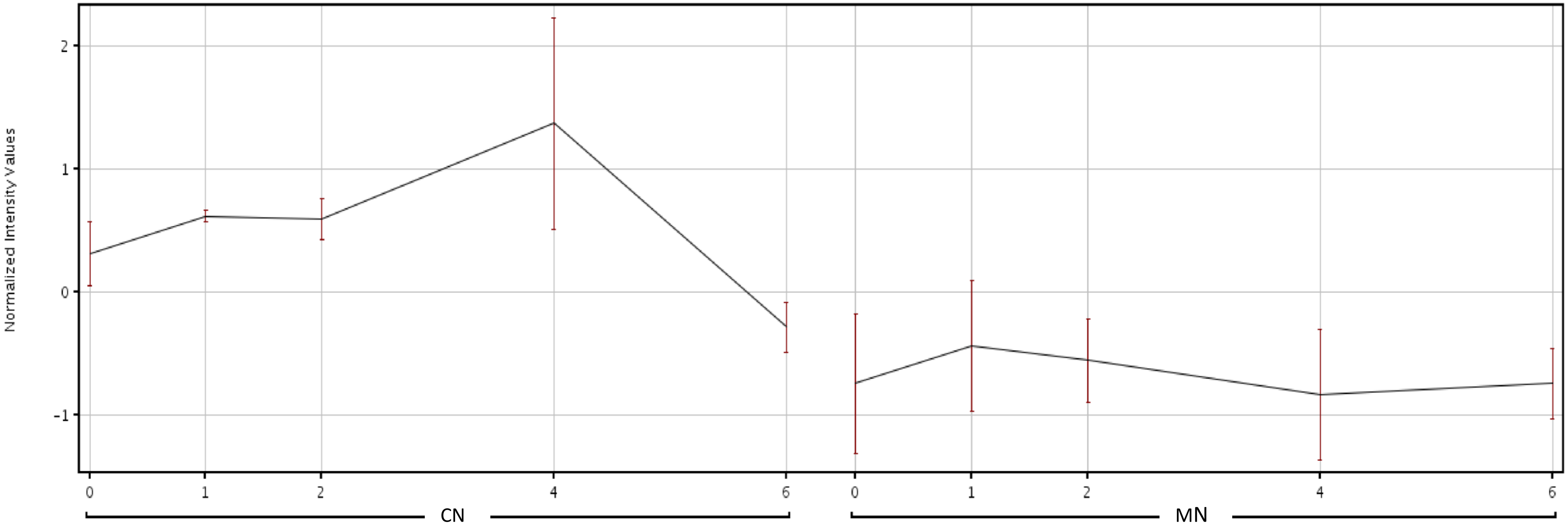

CD40

(K)

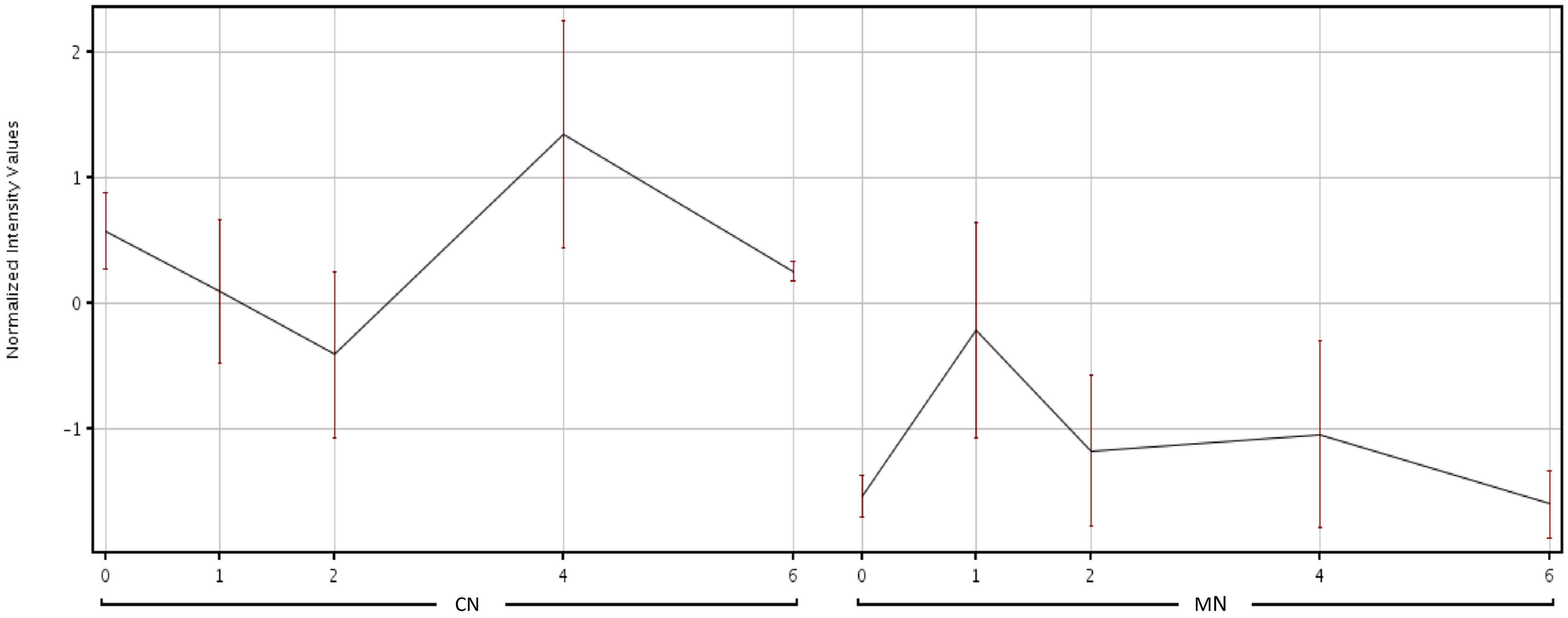

CD80

(L)

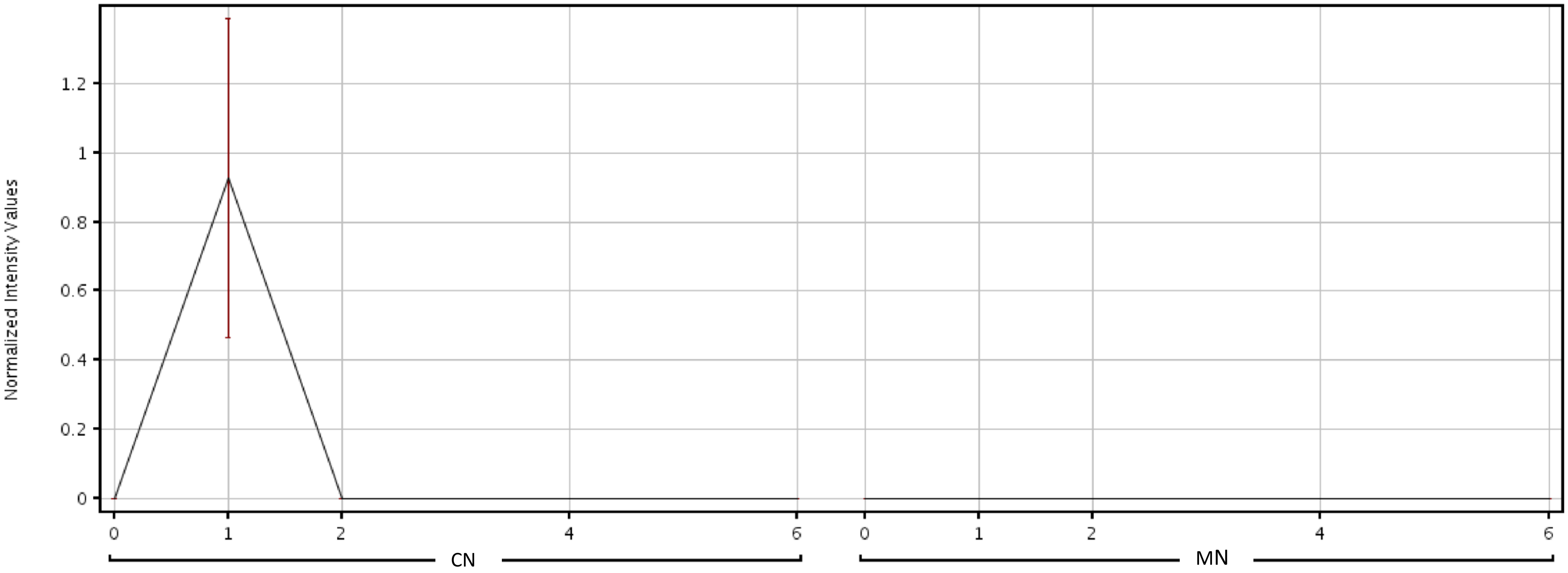

Normalized Intensity Values
1.2
1
0.8
0.6
0.4
0.2
0
0
1
2
4
6
CN
0
1
2
4
6
MN

CD86

(M)

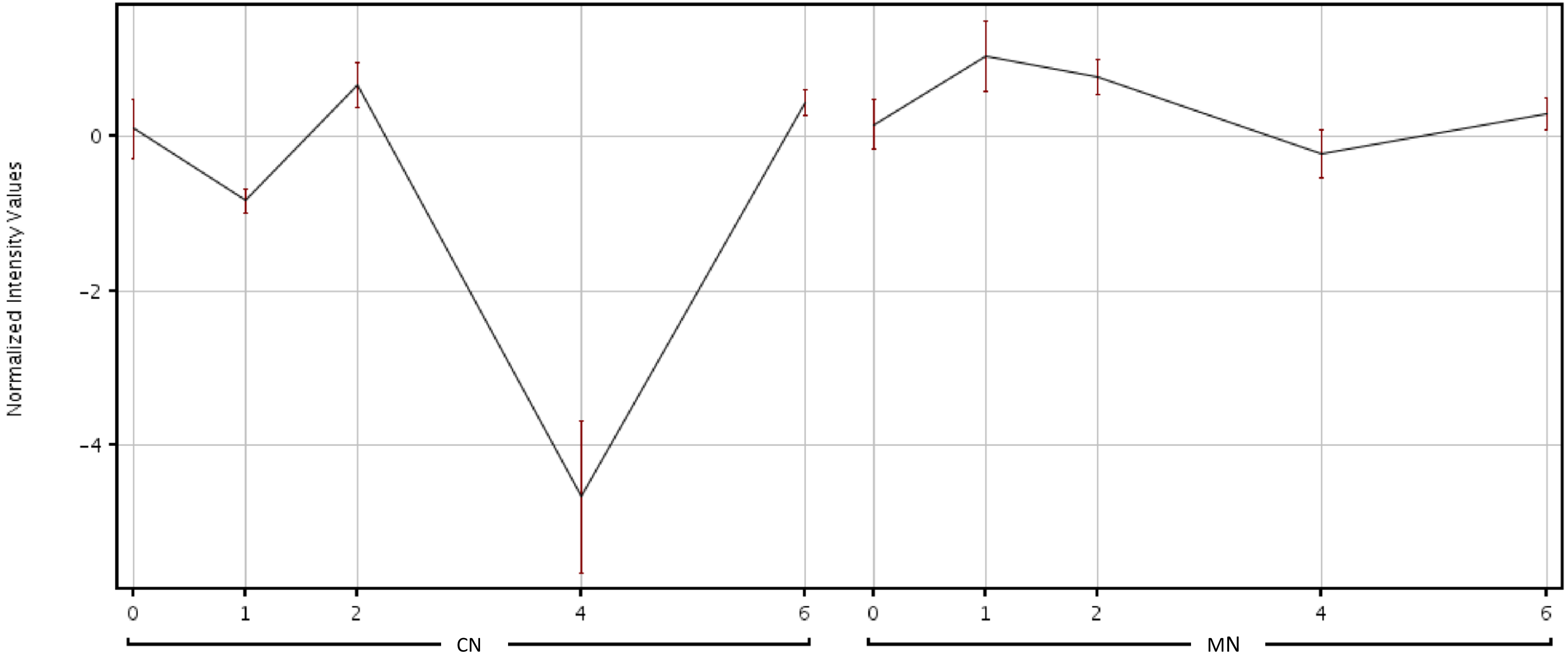

FOS (c-FOS)

(N)

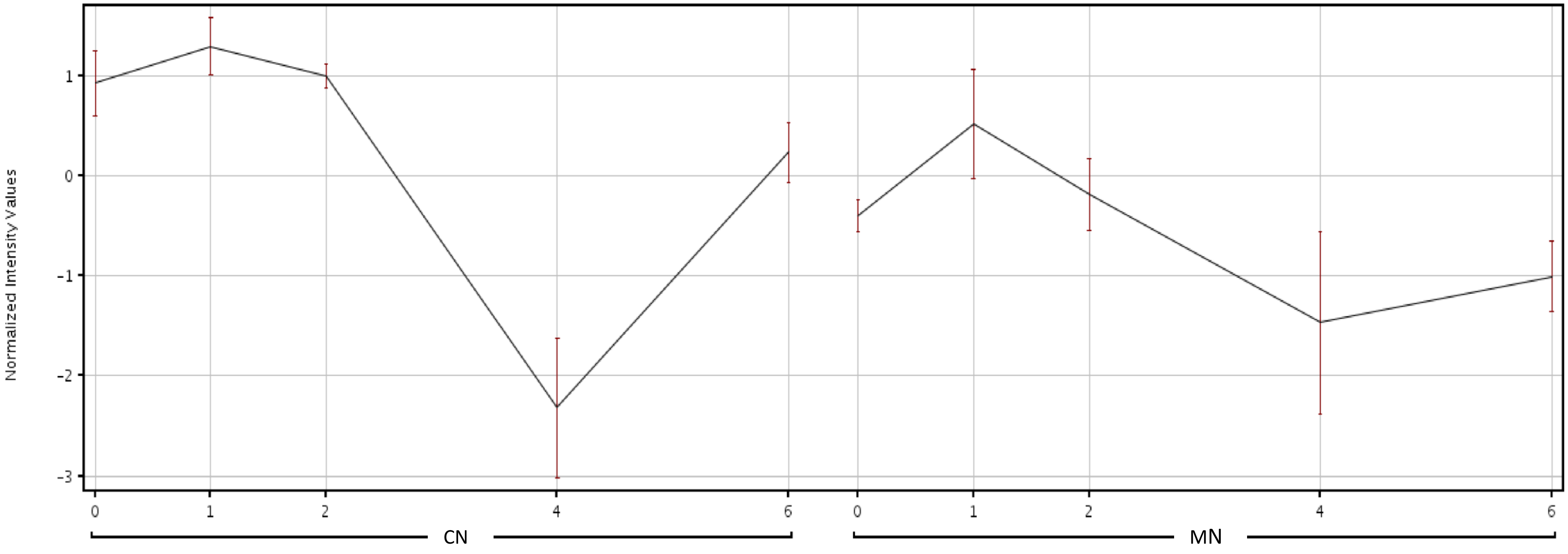

IL7R

(O)

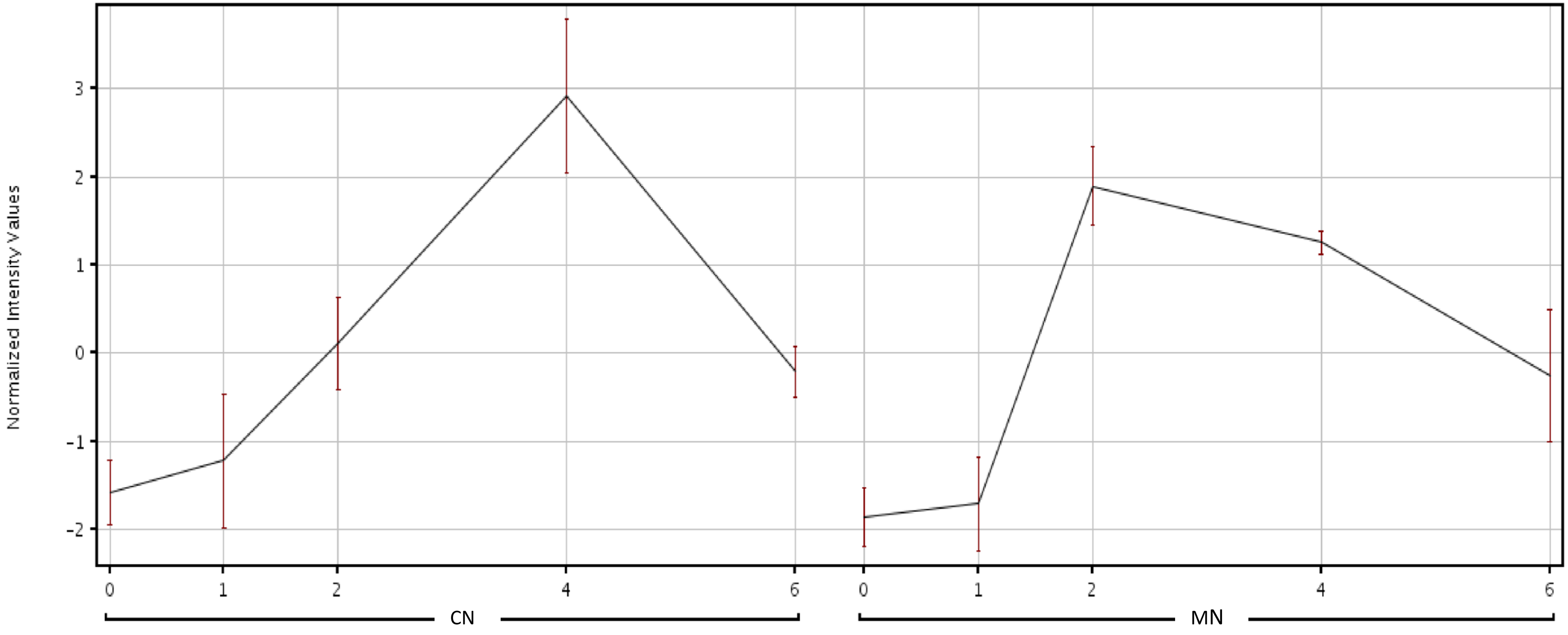

IFIT3

(P)

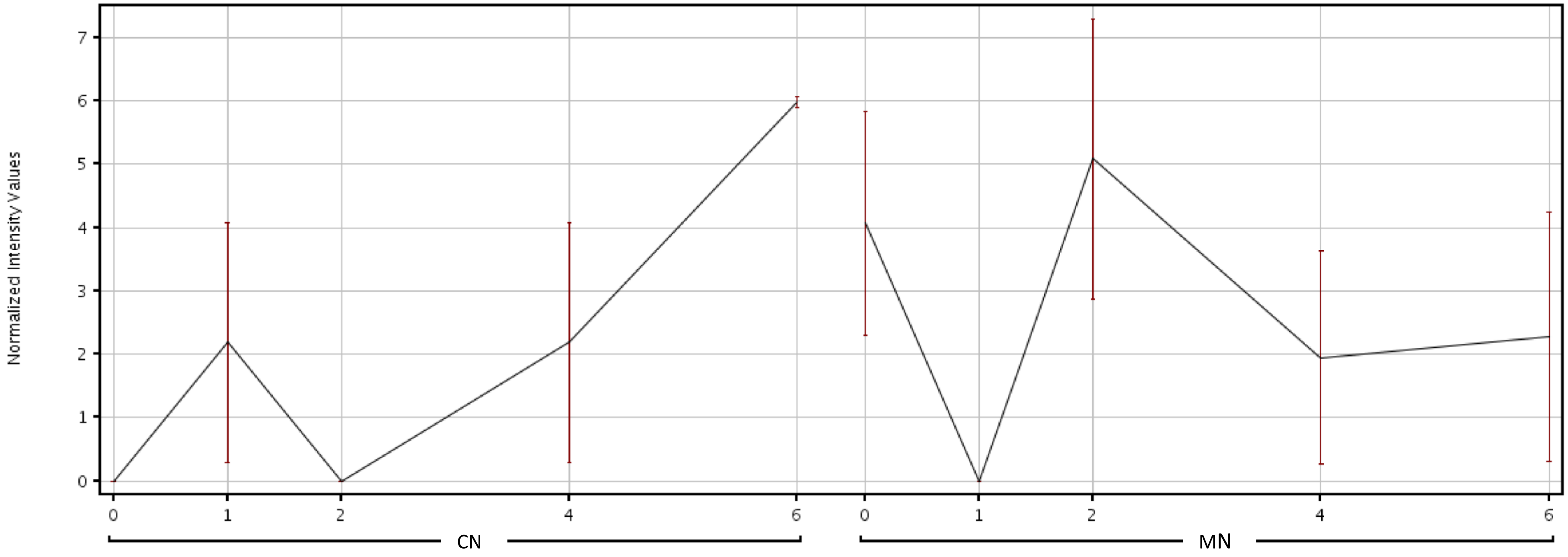

IFIT2

(Q)

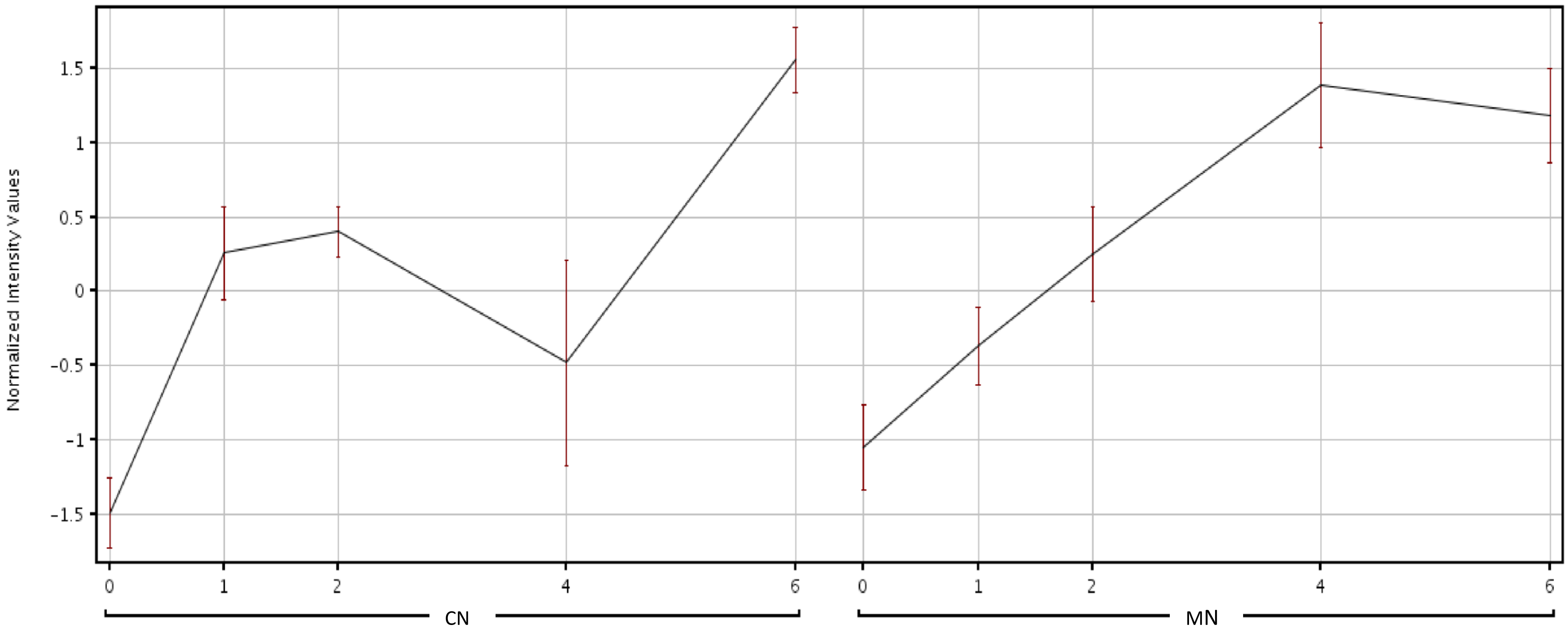
Normalized Intensity Values
1.5
1
0.5
0
-0.5
-1
-1.5
0
1
2
4
6
0
1
2
4
6
CN
MN

FAS

(R)

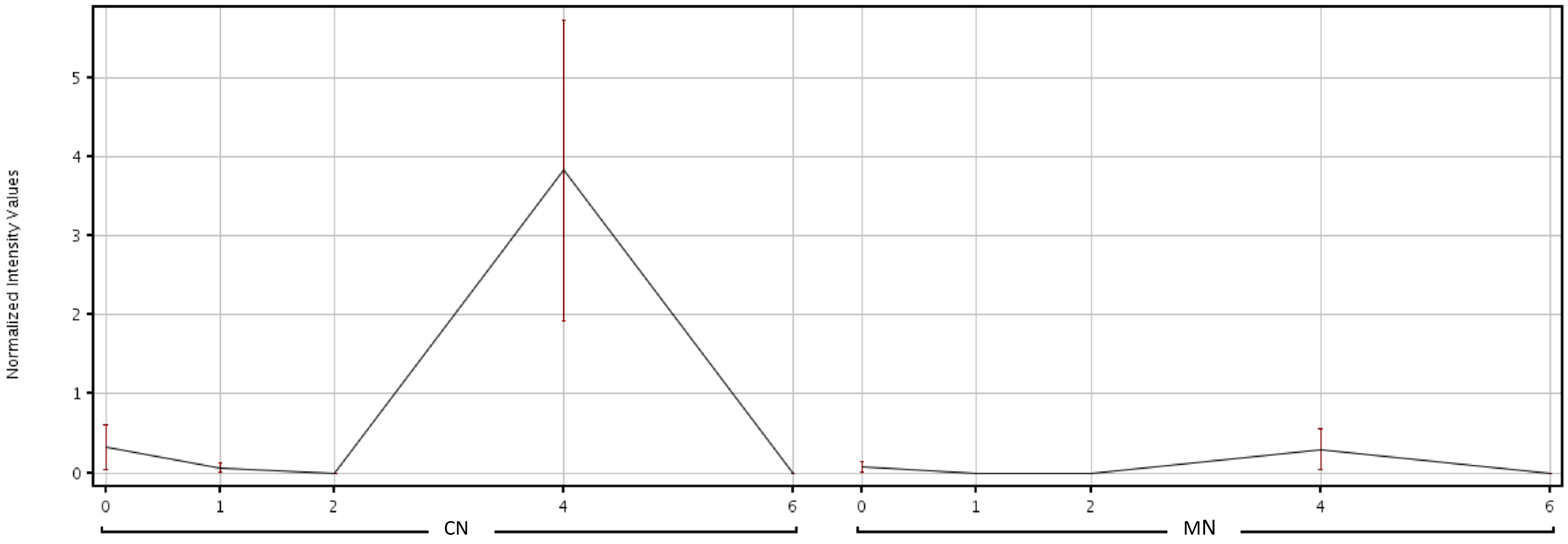

IL1O

(S)

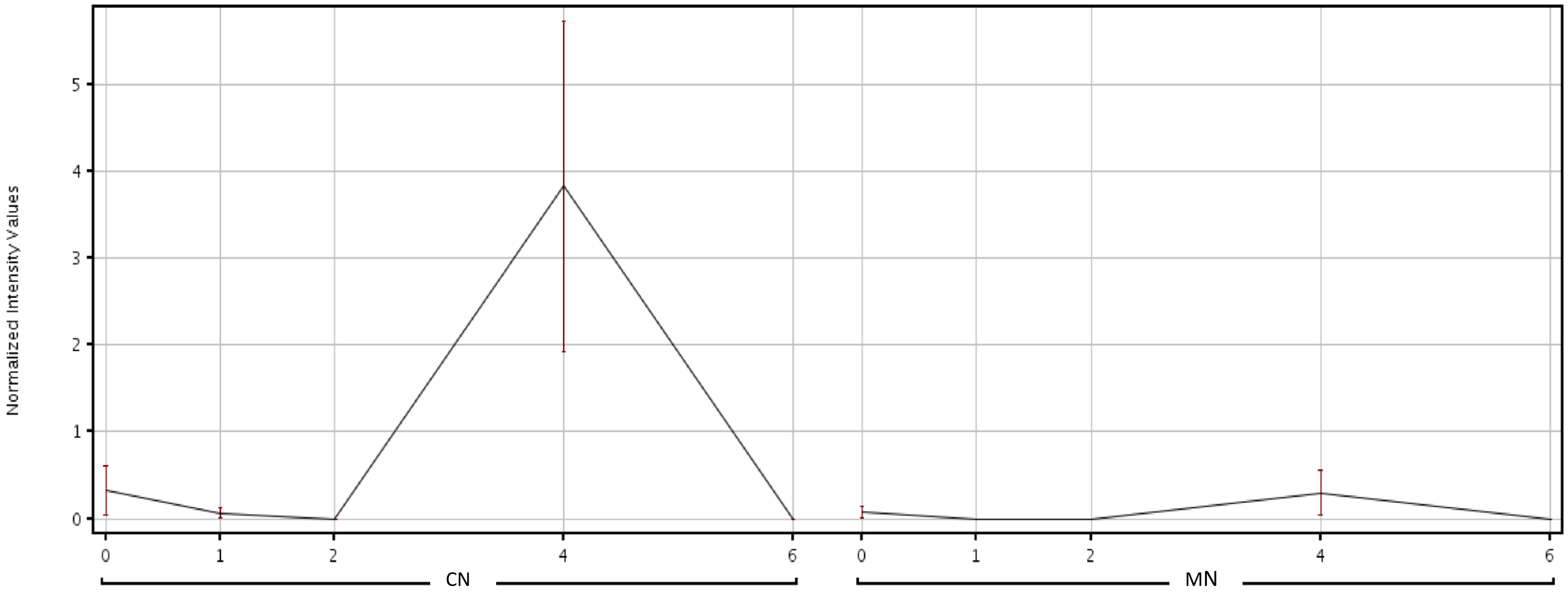

IL11

(T)

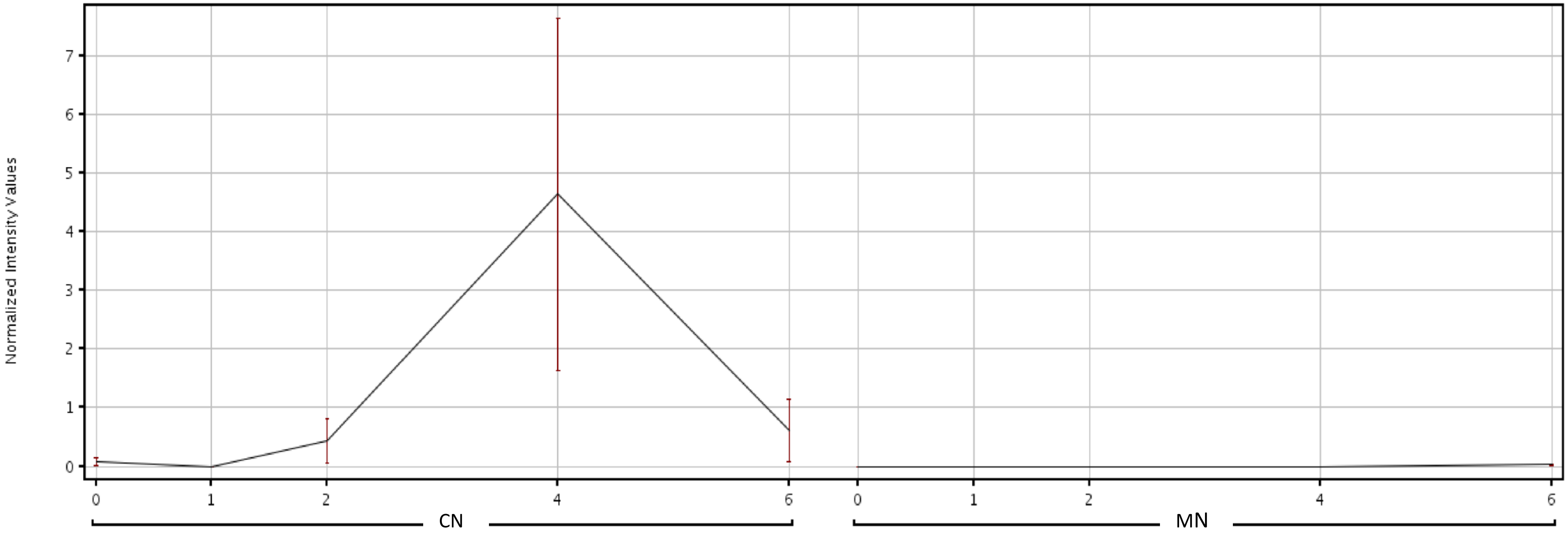

IRF3

(U)

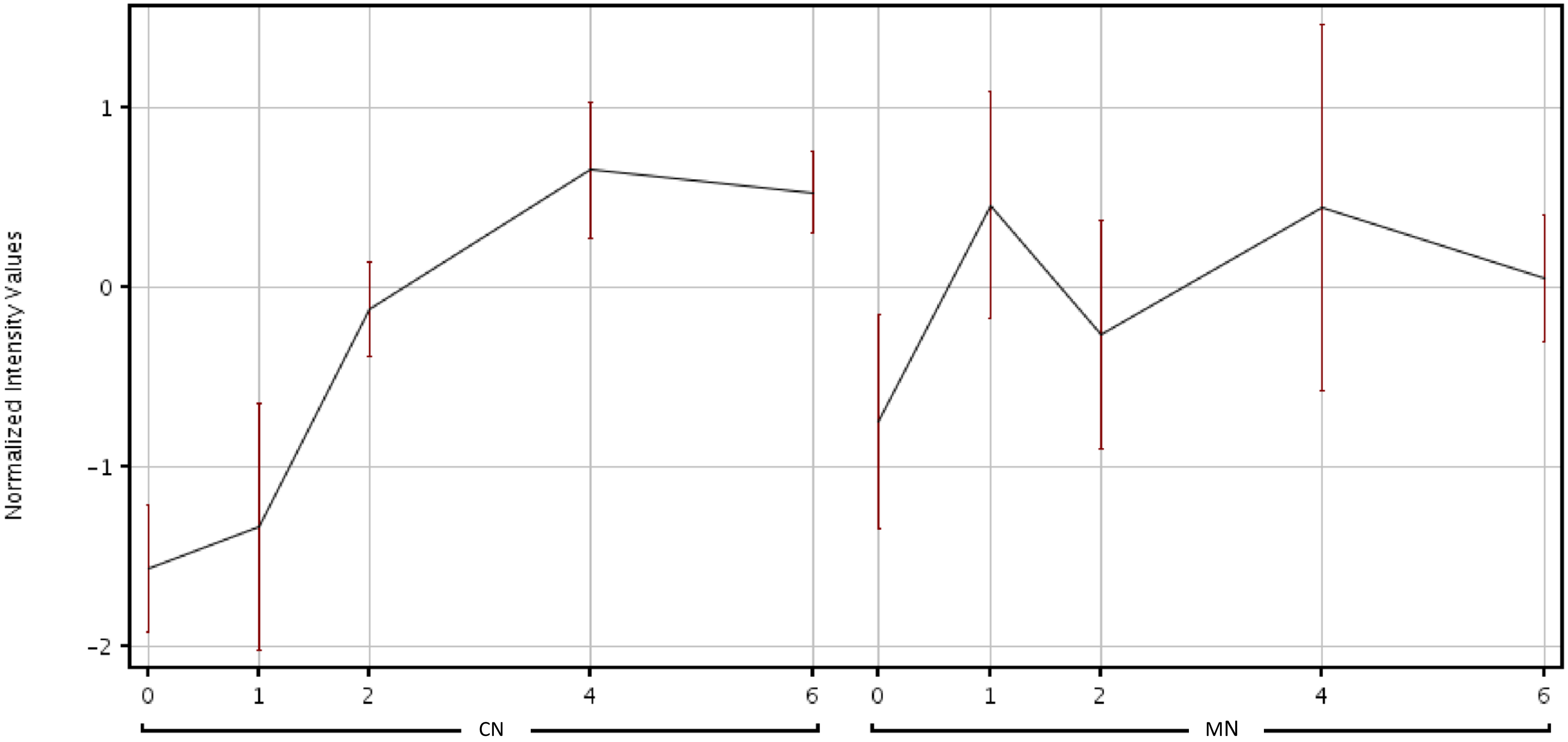

TLR4

(V)

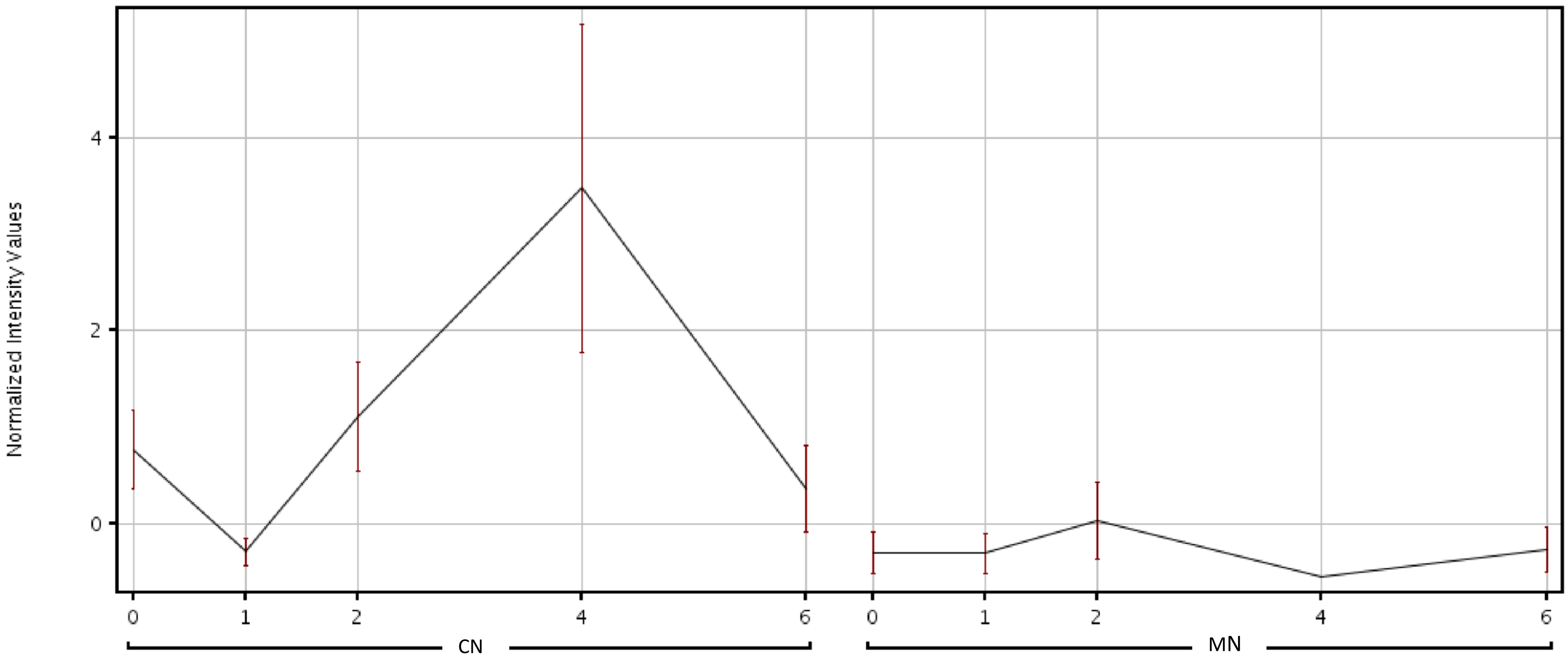

TNF
